# Visible-light-induced one-pot synthesis of sulfonic esters *via* multicomponent reaction of arylazo sulfones and alcohols†

**DOI:** 10.1039/d2ra02656b

**Published:** 2022-06-13

**Authors:** Truong Giang Luu, Tien Tan Bui, Hee-Kwon Kim

**Affiliations:** Department of Nuclear Medicine, Molecular Imaging & Therapeutic Medicine Research Center, Jeonbuk National University Medical School and Hospital Jeonju 54907 Republic of Korea hkkim717@jbnu.ac.kr; Department of Chemistry, Iowa State University Ames Iowa 50011 USA; Research Institute of Clinical Medicine of Jeonbuk National University-Biomedical Research Institute of Jeonbuk National University Hospital Jeonju 54907 Republic of Korea

## Abstract

Sulfonic ester is a chemical structure common to many organic molecules, including biologically active compounds. Herein, a visible-light-induced synthetic method to prepare aryl sulfonic ester from arylazo sulfones was developed. In the present study, a one-pot reaction was carried out using arylazo sulfones, DABSO (DABCO·(SO_2_)_2_), and alcohols in the presence of CuI as a coupling catalyst and HCl as an additive to yield sulfonic esters *via* multicomponent reaction. This synthetic method afforded a wide range of sulfonic esters with high yields under mild conditions.

## Abstract 2

Facile and efficient one-pot synthesis of sulfonic esters has been achieved *via* visible-light-induced multicomponent reaction of arylazo sulfones, and alcohols in the presence of DABSO, CuI, and HCI.

## Graphical abstract

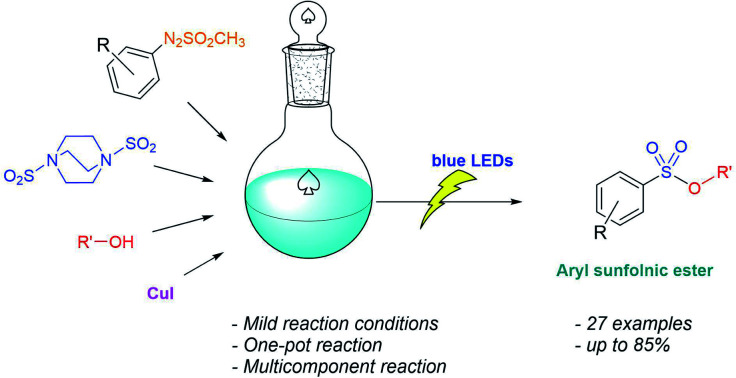

## Introduction

**Fig. 1:**
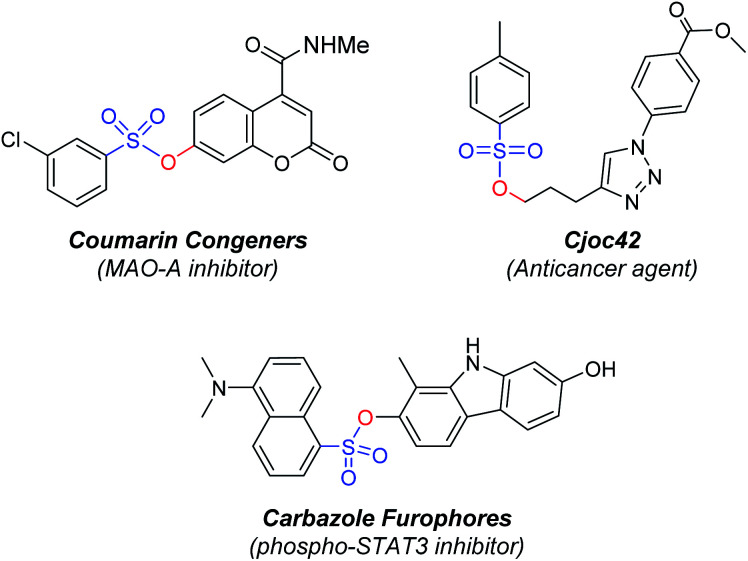
Bioactive compounds containing sulfonic esters.

Organic compounds containing sulfonyl groups such as sulfones, sulfonamide, sulfonic esters, and sulfonyl hydrazine have been important molecules for many organic chemistry and material sciences.^1^ The sulfonic ester motif in particular is featured as a key structure in many organic molecules.^2^ The sulfonic ester moieties, including mesylate, camyslate, tosylate, and besylate, are frequently observed in biologically active compounds for agrochemicals and pharmaceuticals.^3^ For example, compounds containing a sulfonic ester moiety have been reported as effective anticancer agents, antimicrotubule agents, MAO-A inhibitor, and phosphor-STAT3 inhibitor (Fig. 1).^4^

Several synthetic protocols have been developed over the years for the preparation of sulfonic esters due to their importance. One common synthetic strategy for sulfonyl esters relied on the esterification of sulfonyl chlorides in the presence of a base.^5^ However, this procedure has drawbacks such as hazardous application of sulfonyl chlorides, limited tolerance of functional groups, poor stability, and challenging storage and handling protocols.^6^

**Scheme 1:**
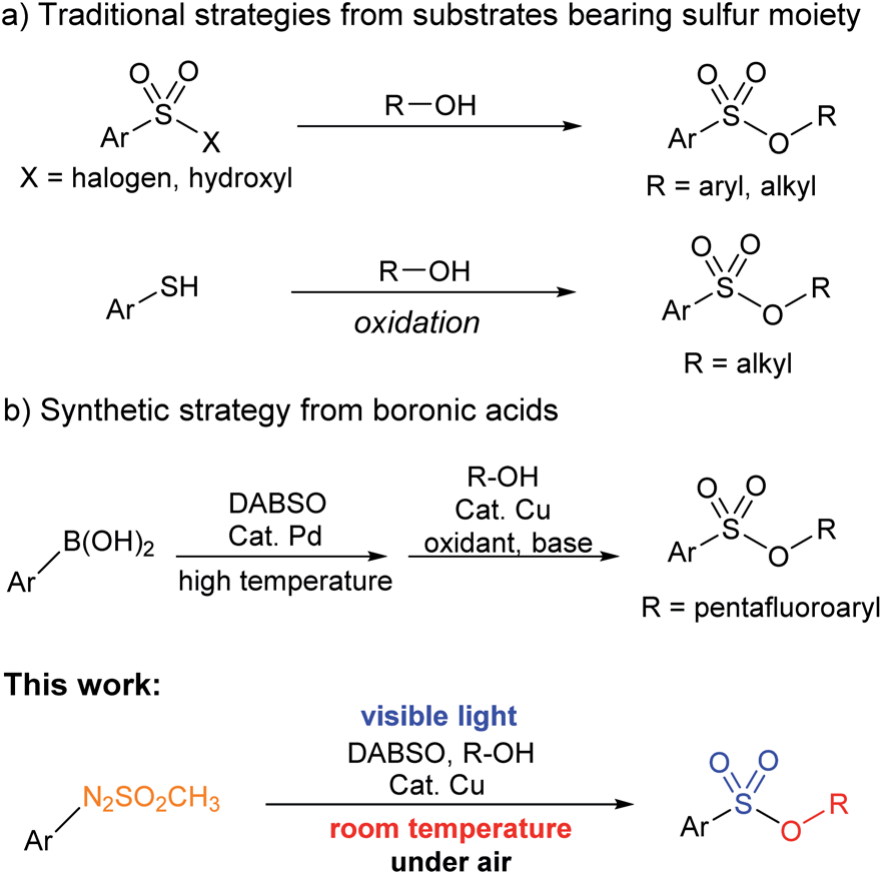
Synthetic procedures to give sulfonic esters.

Several other methods using different types of starting materials, including sulfonic acid,^7^ thiophenols,^8^ and dimethyl and diethyl sulfates,^9^ have also been reported in various studies for the synthesis of sulfonyl esters. In addition, a copper-catalyzed formation of pentafluoroaryl-containing sulfonyl esters using boronic acids was developed.^10^ However, the methods have some limitations including harsh reaction conditions and use of expensive catalysts or reagents. Recently, reaction of aryl diazonium salts for the synthesis of pentafluoroaryl-bearing sulfonyl esters was carried out.^11^ The reaction using aryl diazonium salts required harsh reaction conditions (Scheme 1).

Besides, in the synthesis of compounds containing sulfonic esters, several preparation procedures utilize sulfur dioxide (SO_2_).^12^ SO_2_ is a crucial intermediate in organic synthesis with advantages including its prevalence in nature, affordable cost, and ease of transformation to the sulfonyl structural moiety.^13^ Therefore, SO_2_ has been extensively used as a sulfonyl precursor in organic chemistry for several decades.^14^ However, less desirable attributes of SO_2_ such as its toxicity and gaseous nature prevent its wide usage for organic reactions. Thus, SO_2_ surrogates have been developed as alternative options.^15^ Particularly, DABCO·(SO_2_)_2_ (DABSO) has recently been employed as one of the more useful SO_2_ surrogates, allowing valuable advancement in the preparation of sulfonyl derivatives.^16^

The photochemical reaction strategy has recently been developed, which employs light power as a sustainable energy source because light enables activation of organic molecules. Visible-light-induced reactions could especially provide pathways to create free radical species, which are able to selectively generate C–C as well as C–X bonds.^17^ However, numerous visible-light-induced reactions have employed expensive and toxic photoredox catalysts such as ruthenium, iridium complexes, or organic dyes. Thus, it would be tremendously valuable to develop a photochemical reaction that does not require photoredox catalysts.

Arylazo sulfones have emerged as useful molecules for several organic synthetic processes. Arylazo sulfones were reported to be easily and efficiently produced, and readily divide to give aryl radical and sulfonyl methyl radical under light irradiation *via* homolytic cleavage of the N–S bond.^18^ So, they are employed for synthesis of valuable chemical structures such as aromatic amides,^19^ allylarenes,^20^ arylboronates,^21^ triarylethylenes,^22^ aryl stannanes,^23^ aryl selenides and aryl tellurides,^24^ aryl phosphonates,^25^ as well as for non-catalytic arylation of heterocycles and inactivated arenes.^26^

With the beneficial application of sulfonate ester moiety, discovering a novel and efficient synthetic procedure for sulfonyl esters is highly valuable. Herein, we report a novel photocatalyst-free visible-light-induced synthesis of sulfonate esters from arylazo sulfones under mild reaction conditions.

## Result and discussion

In this study, we assumed that arylazo sulfones could yield aryl radical intermediates under irradiation by visible light. Subsequent treatment with sulfur dioxide surrogate could then provide aryl sulfonyl radicals, which would be converted to sulfonic esters *via* reaction with alcohols.

To verify the feasibility of our concept, novel reaction conditions consisting of sulfonylating agents and alcohols were investigated for the synthesis of sulfonic esters. The initial reaction system using (*E*)-1-(4-methoxyphenyl)-2-(methylsulfonyl) diazene 1a as starting material, DABSO as sulfur dioxide surrogate, MeOH 2a, catalyst, and HCl as acid additive was designed, and the reaction was carried out under the radiation of blue LED light at room temperature for 4 hours.

First, various catalysts able to facilitate sulfonic ester generation were examined. When ZnCl_2_ or SnCl_2_ were used for the reaction, the target product was not observed (Table 1, entries 1 and 2). ZrCl_4_, FeCl_3_, and MnCl_2_ provided the desired sulfonic ester product with low yields (21–25%) (Table 1, entries 3–5). TiCl_4_ could facilitate the reaction for sulfonyl ester with an improved yield (62%) (Table 1, entry 6), while utilization of CuCl_2_ provided the target product at good yield (74%) (Table 1, entry 7). The results suggested that copper salts were effective for generating sulfonyl ester. Thus, investigations of several other copper salts were carried out. Reactions using CuBr_2_, CuCl_2_, Cu(OAc)_2_, and Cu(acac)_2_ provided the target product with good yields (71–75%) (Table 1, entries 9–12) while employment of CuBr did not yield the desired sulfonyl ester compound. When CuI was used as a catalyst for the reaction, the target sulfonyl ester was obtained with 85% yield. So, CuI was chosen as catalyst for generating sulfonyl ester in this reaction.

**Table 1:** Screening of reaction conditions for synthesis of sulfonic esters^a^

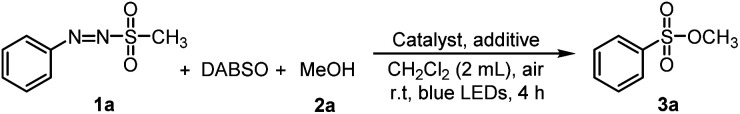				
Entry	Catalyst	SO_2_ source agent	Additive	Yield^b^ (%)
1	ZnCl_2_	DABSO	HCl	Trace
2	SnCl_2_	DABSO	HCl	Trace
3	ZrCl_4_	DABSO	HCl	21
4	FeCl_3_	DABSO	HCl	23
5	MnCl_2_	DABSO	HCl	25
6	TiCl_4_	DABSO	HCl	62
7	CuCl_2_	DABSO	HCl	74
8	CuBr	DABSO	HCl	Trace
9	CuBr_2_	DABSO	HCl	71
10	Cu(OAc)_2_	DABSO	HCl	75
11	Cu(acac)_2_	DABSO	HCl	72
12	CuI	DABSO	HCl	85
13	CuI	Na_2_S_2_O_5_	HCl	Trace
14	CuI	K_2_S_2_O_5_	HCl	Trace
15	CuI	DABSO	CF_3_COOH	70
16	CuI	DABSO	NaHCO_3_	Trace
17	CuI	DABSO	K_2_CO_3_	Trace
18	CuI	DABSO	DIEA	35
19	CuI	DABSO	DBU	37
20	CuI	DABSO	AcOH	45
21	CuI	DABSO	CH_2_BrCOOH	47
22	CuI	DABSO	CF_3_SO_2_H	79
23^c^	CuI	DABSO	HCl	82
24^d^	CuI	DABSO	HCl	37
25^e^	CuI	DABSO	HCl	75

a Reaction conditions: compound 1 (1.0 mmol), SO_2_ source agent (1.0 mmol), catalyst (0.1 mmol), MeOH (20 mmol), additive (1.0 mmol), CH_2_Cl_2_ (2 mL), 4 h, irradiation by 5 W blue LEDs for 4 h.

b Isolated yield after purification by flash column chromatography.

c Irradiation by 5 W white LEDs.

d Irradiation by 5 W green LEDs.

e Irradiation by 20 W white CFL.

Next, several sulfur dioxide surrogates as SO_2_ source agents were examined to optimize the synthesis of sulfonyl ester (Table 1, entries 12–14). Employment of Na_2_S_2_O_5_ or K_2_S_2_O_5_ did not provide the target sulfonyl esters (Table 1, entries 13 and 14). However, DABSO, a DABCO·(SO_2_)_2_ complex, was successfully used for the reaction, and the target sulfonyl ester was synthesized with high yield (Table 1, entry 12).

Additives were an important factor affecting the reaction yield. Thus, different additives were tested. In reactions using inorganic bases such as NaHCO_3_ and K_2_CO_3_, no desired sulfonyl ester product was prepared. Utilization of organic bases such as DIEA and DBU afforded the corresponding products in low yield (35–37%) (Table 1, entries 18 and 19). In contrast, when acids such as AcOH and CH_2_BrCOOH were used as additives, sulfonyl ester was synthesized with improved conversion yields (45–47%) (Table 1, entries 20–21). Additionally, in reactions employing CF_3_SO_2_H and HCl as additives, the target products were prepared with high yields (79 and 85%) (Table 1, entries 22 and 12).

The influence of light source on reaction efficiency was examined using several types of light sources. The replacement of blue LEDs by white LEDs, green LEDs, or white CFLs did not give an enhanced yield (Table 1, entries 23–25). The reaction of arylazo sulfone 1a, DABSO, and methanol 2a under 5 W white LEDs affords the desired product 3a in 82% yield which was lower than that of the reaction under 5 W blue LEDs with 85%. Therefore, using blue LEDs is suitable for this reaction. Thus, blue LEDs were indicated as a suitable light source for this reaction.

Solvent effect on the reaction was evaluated to find an optimal reaction protocol (Table 2). Employment of DMF, 1,4-dioxane did not yield the target sulfonic ester product. In the reaction in THF, toluene, and MeCN, the desired products were prepared in less than 40% yield. When DCE was used as reaction solvent, the sulfonic ester product was generated in increased yield. Especially, CH_2_Cl_2_ solvent showed a better reaction performance with high yield, suggesting that CH_2_Cl_2_ was a suitable solvent for sulfonic ester synthesis.

**Table 2:** Screening of solvents for the preparation of sulfonic esters^a^

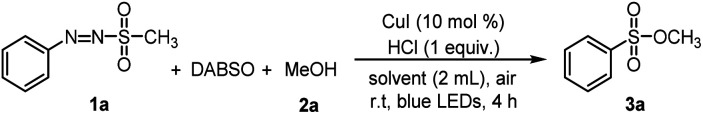		
Entry	Solvent	Yield^b^ (%)
1	1,4-Dioxane	Trace
2	DMF	Trace
3	THF	22
4	Toluene	35
5	MeCN	39
6	DCE	73
7	CH_2_Cl_2_	85

a Reaction conditions: compound 1 (1.0 mmol), MeOH (20 mmol), DABSO (1.0 mmol), CuI (0.1 mmol), HCl (1.0 mmol), irradiation by 5 W blue LEDs for 4 h.

b Isolated yield after purification of flash column chromatography.

Next, various amounts of reagents were examined to evaluate their efficiency for synthesis of sulfonic esters (Table S1†). A series of different amounts of CuI were tested for the reaction to discover optimal amount of CuI (Table S1†). Addition of more than 0.1 equiv. of CuI did not provide any significant enhancement of synthetic yield (85% for 0.2, 0.5, 1.0 equiv. of CuI). The finding suggested that 0.1 equiv. of CuI was the proper amount to generate sulfonic ester in this method.

Investigation of the optimal amount of DABSO was also carried out for the reaction (Table S2†). Reactions using less than 0.1 equiv. of DABSO did not provide a satisfactory synthetic yield (43% for 0.8 equiv. of DABSO), while reactions in the presence of 1.0 equiv. of DABSO or higher successfully afforded the corresponding product in high yield. However, there was no significant increase in synthetic yield of the reaction when using higher than 1.0 equiv. of DABSO.

**Scheme 2:**
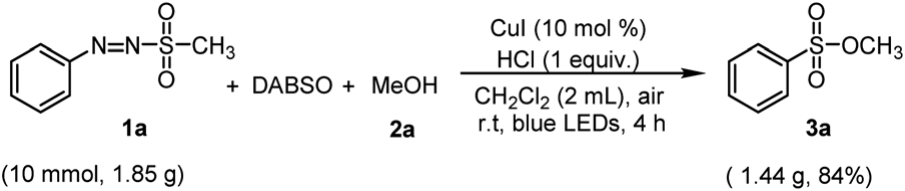
Gram-scale reaction of arylazo sulfone (1a).

With the optimized reaction conditions in hand, the scope for visible-light-induced synthesis of sulfonic ester was investigated. First, reactions of a variety of arylazo sulfones were carried out with methanol and DABSO in the presence of CuI and HCl (Table 3). Arylazo sulfones bearing different substituents were well tolerated for this synthetic method. Synthesis of sulfonic esters from arylazo sulfones with electron withdrawing substituents (chloro- and nitro-) at the *para* position was readily achieved (3b and 3c). Similarly, reactions using arylazo sulfones with electron-donating groups (methyl, *tert*-butyl, and methoxy) at *para* position also afforded the target sulfonic esters in 78–82% yields (3e–3g). In addition, sulfonic esters with various functional groups including acetyl and benzyl protected hydroxyl groups were easily synthesized *via* visible-light-induced reaction of arylazo sulfone substrates (Scheme 2, compounds 3d and 3h). Arylazo sulfone substrates with substituents at *meta* position also reacted well to afford the desired sulfonic esters 3i–3j in the yield range from 51 to 73%. Reactions of arylazo sulfones bearing di-substituted groups were investigated under the same reaction conditions, and the desired products such as 3,5-dichloro-substituted sulfonic ester 3k and 3,5-dimethyl-substituted sulfonic ester 3l were successfully synthesized in good yield. Moreover, substrates containing naphthalene and heterocyclic substituted rings were employed for novel visible-light-induced reaction, and they worked well with this reaction: methyl naphthalene-1-sulfonate and methyl benzo[*d*][1,3]dioxole-4-sulfonate were readily prepared in 75% and 83% yield, respectively (3m and 3n). To investigate the applicability of this method in pharmaceuticals, substrate obtained from pharmaceutical compound was employed for this procedure. Reaction of the arylazo sulfone prepared from sulfamethazine, an antibacterial medicine, smoothly yielded the target sulfonic ester 3o.

**Table 3:** Substrate scope of arylazo sulfone^a^^,^^b^

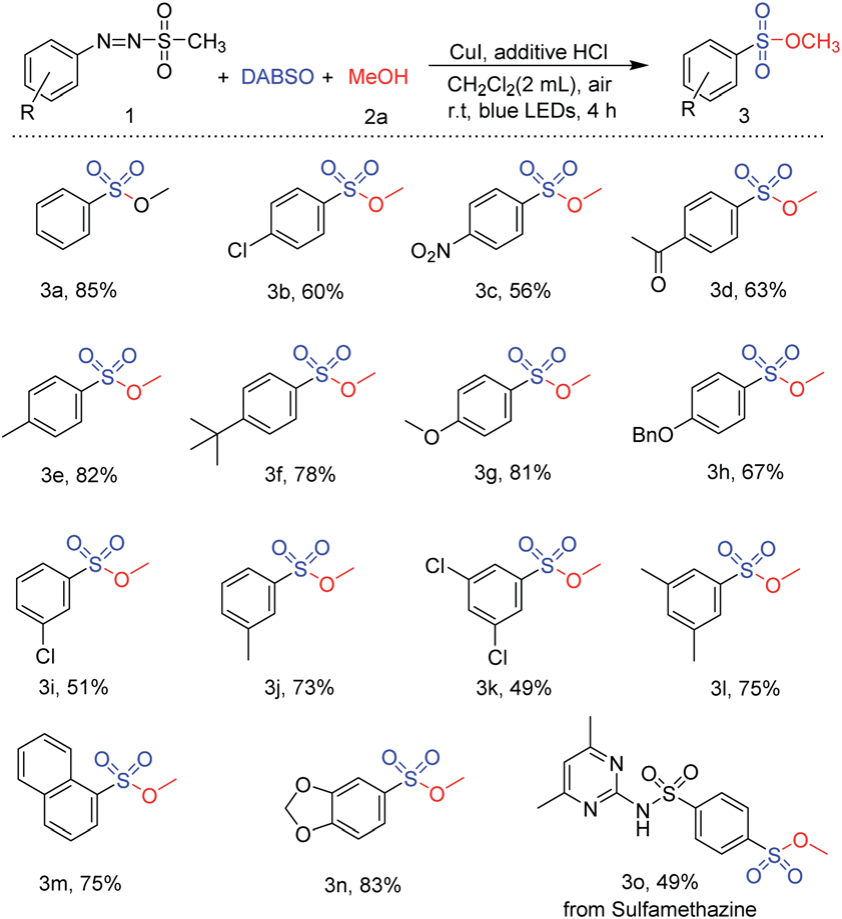

a Reaction conditions: compound 1 (1.0 mmol), MeOH (20 mmol), DABSO (1.0 mmol), CuI (0.1 mmol), HCl (1.0 mmol), CH_2_Cl_2_ (2 mL), irradiation by 5 W blue LEDs for 4 h.

b Isolated yield after purification of flash column chromatography.

Next, the reaction was explored using various alcohols instead of methanol (Table 4). Synthesis of aryl sulfonyl ester was readily achieved *via* treatment of primary alcohol such as ethanol, *n*-butanol, *n*-heptanol, and iso-butyl alcohol with 1-(methylsulfonyl)-2-aryldiazene (61–79%) (4a–4d). Benzyl alcohol was also tested for the reaction, providing the desired product in 73% yield. A reaction with cyclohexanol, a secondary alcohol, was additionally evaluated, and cyclohexyl benzenesulfonate was readily synthesized in a good yield (4f). Moreover, *tert*-butanol, a bulky tertiary alcohol, was employed to react with several arylazo sulfones. Reactions of substrates bearing electron-withdrawing substituents (nitro-) and electron-donating substituents (methyl-, ethyl-, methoxyl-, and benzyloxy) with *tert*-butanol successfully gave the corresponding sulfonic esters in 45–76% yield under the same conditions of reaction.

**Table 4:** Substrate scope of alcohols^a^^,^^b^

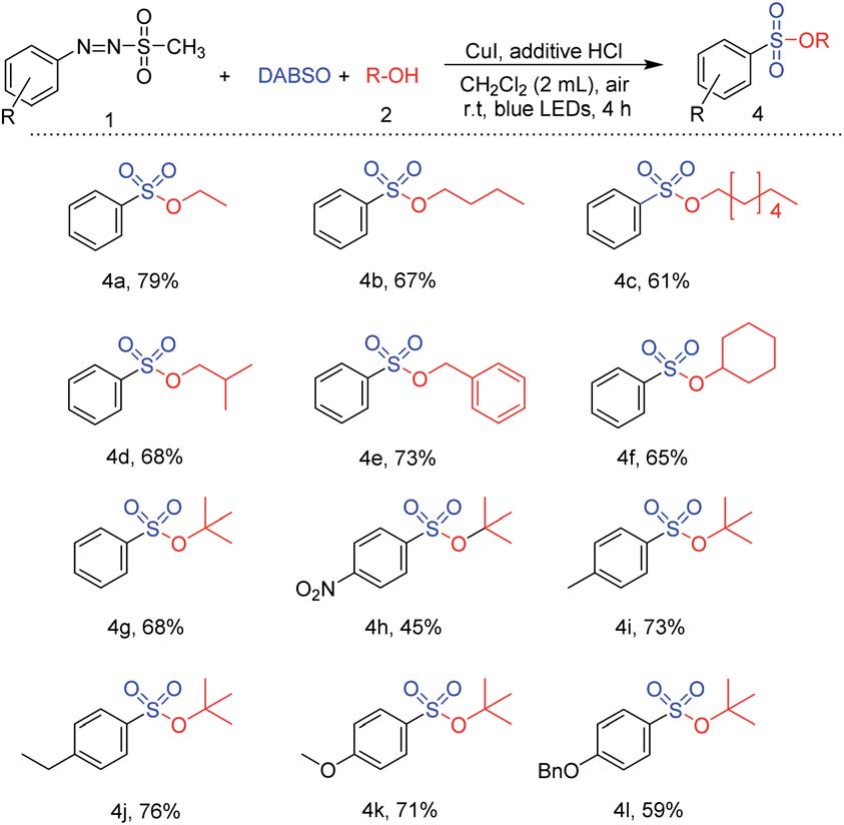

a Reaction conditions: compound 1 (1.0 mmol), alcohol (20 mmol), DABSO (1.0 mmol), CuI (0.1 mmol), HCl (1.0 mmol), CH_2_Cl_2_ (2 mL), irradiation by 5 W blue LEDs for 4 h.

b Isolated yield after purification of flash column chromatography.

It is note that novel synthetic method was better than the previous procedure reported by Han and co-workers (*J. Org. Chem.*, 2018, **83**, 4674–4680).^31^ The reaction time (4 h) of this study was shorter than 12 h of the previous method. In addition, these reaction yields of this study were better than those of the previous method (for example, compound 3a in this study was generated with 85% yield *vs.* 50% yield in the previous method. Similarly, 82% *vs.* 64% for compound 3e; 60% *vs.* 41% for compound 3b; 73% *vs.* 29% for compound 4i). Thus, the new visible-light-induced reaction of arylazo sulfones provided a more efficient synthetic protocol to prepare sulfonic esters rather than that of the previous method.

Next, a large-scale synthesis of sulfonic esters from arylazo sulfones was performed to see the utility of the procedure (Scheme 2). In reaction of arylazo sulfone 1a (10.0 mmol, 1.85 g) with MeOH 4a, the target sulfonic ester was successfully synthesized in 84% yield *via* the same reaction condition, suggesting that this reaction method can be effective and scalable.

**Scheme 3:**
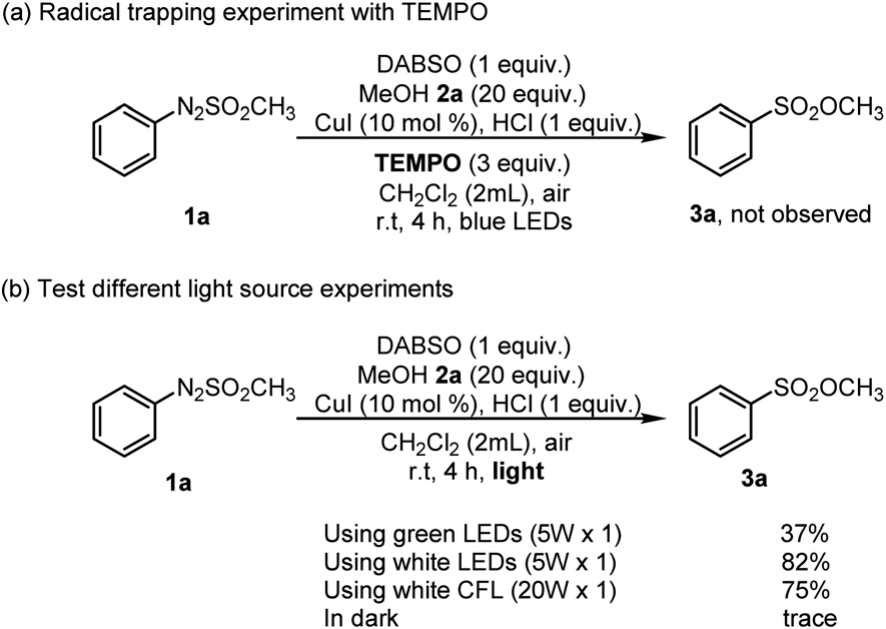
Control experiments.

To get an idea for the mechanism of this reaction, control reactions were performed (Scheme 3). First, 3.0 equiv. of TEMPO (2,2,6,6-tetramethyl-1-piperidinyloxy), a radical scavenger, was employed for a reaction system consisting of arylazo sulfone 1a, DABSO, and methanol 2a, and the conversion of arylazo sulfones 1a into the corresponding sulfonic ester 3a was fully inhibited by TEMPO (Scheme 3a). The fact that no sulfonic ester product 3a was formed during reaction in the presence of TEMPO suggested that a radical pathway should be involved in this reaction. Next, the reaction was carried out in the dark, and it was found that no desired product 3a was observed (Scheme 3b).

The UV-vis spectra of some arylazo sulfone compounds in reaction solvent CH_2_Cl_2_, as well as in the presence of DABSO and CuI, have been quantified (Fig. S2–S5†). The results show that arylazo sulfone compounds exhibit absorption maxima located at 410–460 nm wavelength. This wavelength range belongs to the blue region in the visible light. Therefore, using blue LEDs is suitable for this reaction.

Moreover, we investigated the role of visible light for this transformation by performing light on/off experiments (Fig. S1†). The reaction was almost suppressed when carried out in the dark, while the desired product 3a was smoothly prepared with increasing yield when starting substrate 2a was irradiated by blue LEDs. The light on/off experiment of the model reaction indicated that continuous irradiation of visible light is important for the progress of this reaction.

**Scheme 4:**
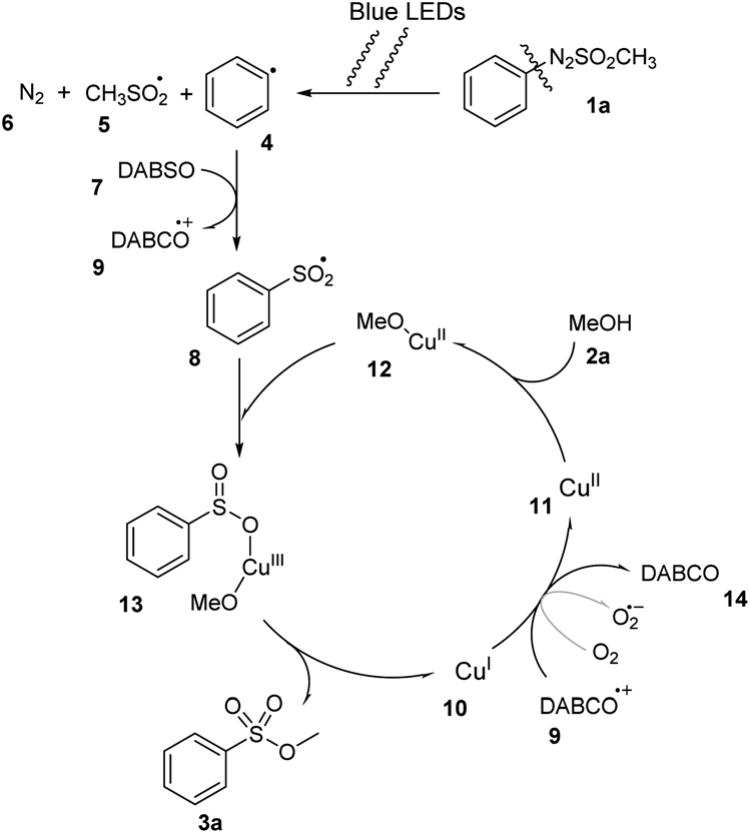
Proposed reaction mechanism.

With the information from the control experiments and previously published literature,^19^ we proposed a plausible mechanism for this reaction (Scheme 4). Initially, under irradiation of blue LED light, arylazo sulfone compounds 1a were homolytically divided at N–S bond to generate aryl radical 4.^27–30^ The radical 4 captured an SO_2_ group of DABSO 7 to form arylsulfonyl radical 8 and the DABCO cation 9. The catalyst Cu^1+^10 was oxidized by DABCO cation 9 or other oxidizing agents (O_2_) to give Cu^2+^11, which reacted with MeOH 2a to form complex Cu^2+^–OMe 12. The complex 12 then reacted with the arylsulfonyl radical 8 to give intermediate 13. A reductive elimination process of 13 generated the desired compound 3a, and recovered Cu^1+^10.

## Conclusions

In summary, a new methodology for visible-light-induced synthesis of sulfonic esters from arylazo sulfones was described. In the present study, multi-component reaction using arylazo sulfones, DABSO, alcohols, and CuI was successfully achieved by the irradiation of blue LED light. A wide range of arylazo sulfone substrates with different substituents and several alcohols were well tolerated in this synthetic method. This novel visible-light-induced reaction produced various sulfonic esters under mild reaction conditions. The results suggested that this efficient synthetic approach using visible light can serve as a useful tool to afford a variety of the highly valuable arylsulfonic esters.

## Experimental

### General procedure of the synthesis of sulfonic esters (3a–3o, 4a–4l)

In a typical synthetic procedure, arylazo sulfone compound 1a (185 mg, 1.0 mmol), DABSO (240 mg, 1.0 mmol), and CuI (19 mg, 0.1 mmol) were added to a mixed solution of dichloromethane (2 mL) and methanol (640 mg, 20 mmol). Hydrochloric acid 37% (100 mg, 1.0 mmol) was dropped into the reaction mixture. The mixture was stirred at room temperature under irradiation by blue LEDs. After 4 hours, the mixture was extracted with 50 mL of CH_2_Cl_2_ and washed with 50 mL of brine solution. The organic layer was dried by sodium sulfate and concentrated under reduced pressure. The residue was purified using flash column chromatography on silica gel with hexane-EtOAc as the eluent to get the intended product 3a (146 mg, 85%).

## Author contributions

H.-K. Kim conceived and designed this work. T. G. Luu and T. T. Bui performed the experiments and collected data. All authors analysed the data, discussed the results, and wrote the manuscript.

## Conflicts of interest

There are no conflicts to declare.

## Supplementary Material

RA-012-D2RA02656B-s001
