# Supplementary material for: Visible-light-induced one-pot synthesis of sulfonic esters via multicomponent reaction of arylazo sulfones and alcohols
Source: RSC Adv. 2022 Jun 13;12(27):17499–504. doi: 10.1039/d2ra02656b (PMC9190201; doi:10.1039/d2ra02656b)
Supplement: RA-012-D2RA02656B-s001 [file RA-012-D2RA02656B-s001.pdf]

Electronic Supplementary Material (ESI) for RSC Advances.
This journal is © The Royal Society of Chemistry 2022

Supplementary Information

Visible-Light-Induced One-Pot Synthesis of Sulfonic Esters via Multicomponent Reaction of Arylazo Sulfones and Alcohols

Truong Giang Luu,[a] Tien Tan Bui,[a,b] and Hee-Kwon Kim*[a,c]

[a] Department of Nuclear Medicine, Molecular Imaging & Therapeutic Medicine Research Center, Jeonbuk National University Medical School and Hospital, Jeonju, 54907, Republic of Korea

[b] Department of Chemistry, Iowa State University, Ames, Iowa 50011, United States

[c] Research Institute of Clinical Medicine of Jeonbuk National University-Biomedical Research Institute of Jeonbuk National University Hospital, Jeonju, 54907, Republic of Korea

* Corresponding author.

Tel: +82 63 250 2768; Fax: +82 63 255 1172.

E-mail address: hkkim717@jbnu.ac.kr (H. Kim).

Table of Contents

Table of Contents S1
General Information S2
Screening of reaction conditions for the synthesis of sulfonic ester compounds..........................S3
Light on/off experiment...S5
UV-Vis Spectroscopic Studies S6
General procedure of the synthesis of sulfonic ester compounds S8
Quantum yield measurement...S8
Chracterization of sulfonic ester compounds S10
^{1}H and ^{13}C NMR Spectra...S21

1. General Information

Commercial chemicals and solvents were used without any purification. Niteo Blue LED lamp (5W, 400-500nm, λ_{max} = 450nm) was used for irradiation reaction. Reaction progress was analyzed by thin-layer chromatography (TLC) using silica gel 60 F_{254} pre-coated aluminum plate from Merck and TLC spots were observed under UV light (254nm) exposure. Flash chromatography was carried out using 230–400 mesh silica gel and analytical grade solvents. Stuart SMP10 Melting Point Apparatus was used to record melting points of products. The UV2600-SHIMADZU spectrophotometer was used to carried out UV-Vis spectroscopic studies. Structure elucidation by NMR (^{1}H and ^{13}C NMR) was performed on Bruker Avance 400 MHz spectrometer. The chemical shifts were reported in δ units (ppm) relative to the residual protonated solvent resonance, the coupling constants (J) quoted in Hz, and multiplicity of signals was abbreviated as follows: singlet (s); doublet (d); doublet of doublet (dd); triplet (t); multiplet (m).

2. Screening of reaction conditions for the synthesis of sulfonic ester compounds

Table S1. Screening of amount of CuI for the preparation of sulfonic esters[a]

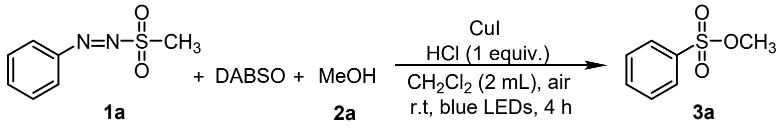

Entry	CuI	Yield[b] (%)
1	CuI (1.0 equiv.)	85
2	CuI (0.5 equiv.)	85
3	CuI (0.2 equiv.)	85
4	CuI (0.1 equiv.)	85
5	CuI (0.05 equiv.)	51
6	CuI (0.02 equiv.)	34
7	CuI (0.01 equiv.)	trace

[a] Reaction conditions: compound **1** (1.0 mmol), MeOH (20 mmol), DABSO (1.0 mmol), HCl (1.0 mmol.), CH_2Cl_2 (2 mL), irradiation by 5 W blue LEDs for 4h. [b] Isolated yield after purification of flash column chromatography.

Table S2. Screening of of different sulfonyl sources for synthesis of sulfonic esters[a]

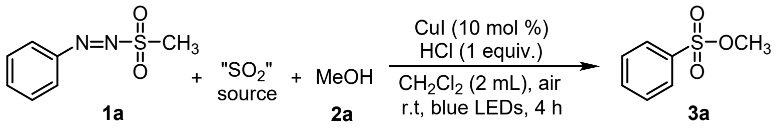

Entry	SO_2 source	Yield[b] (%)
1	DABSO (0.8 equiv.)	43
2	DABSO (1.0 equiv.)	85
3	DABSO (1.2 equiv.)	85
4	DABSO (1.5 equiv.)	85
5	DABSO (2.0 equiv.)	85
6	DABSO (3.0 equiv.)	85
7	$Na_2S_2O_5$ (1.0 equiv.)	Trace
8	$Na_2S_2O_5$ (2.0 equiv.)	Trace
9	$Na_2S_2O_5$ (3.0 equiv.)	Trace
10	$K_2S_2O_5$ (1.0 equiv.)	Trace
11	$K_2S_2O_5$ (2.0 equiv.)	Trace
12	$K_2S_2O_5$ (3.0 equiv.)	Trace

[a] Reaction conditions: compound **1** (1.0 mmol), MeOH (20 mmol), CuI (0.1 mmol), HCl (1.0 mmol), CH_2Cl_2 (2 mL), irradiation by 5 W blue LEDs for 4h. [b] Isolated yield after purification of flash column chromatography.

3. Light on/off experiments

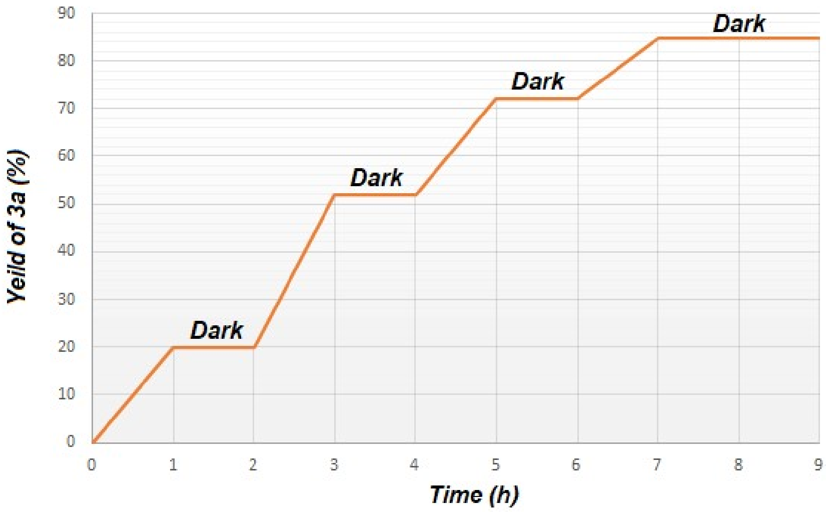

Fig. S1 Light on/off experiments of compound 3a.

4. UV-Vis Spectroscopic Studies

The UV-Vis spectra of some arylazo sulfone compounds in reaction solvent CH_2Cl_2, as well as in the presence of DABSO and CuI, have been quantified. Basically, arylazo sulfone compounds exhibit absorption maxima located in the blue light range.

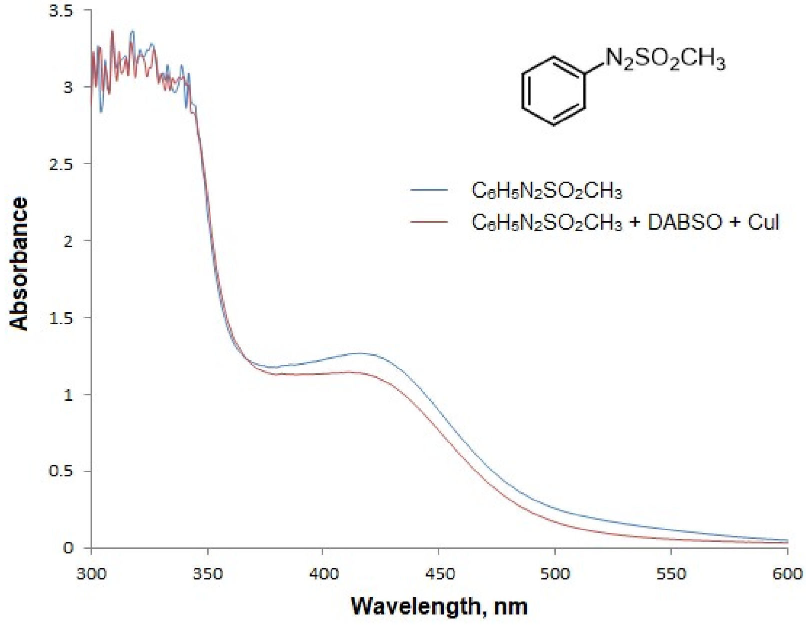

Fig. S2 UV-Vis absorption spectrum of compound **1a** in CH_2Cl_2 and compound **1a** in the presence of DABSO and CuI in CH_2Cl_2

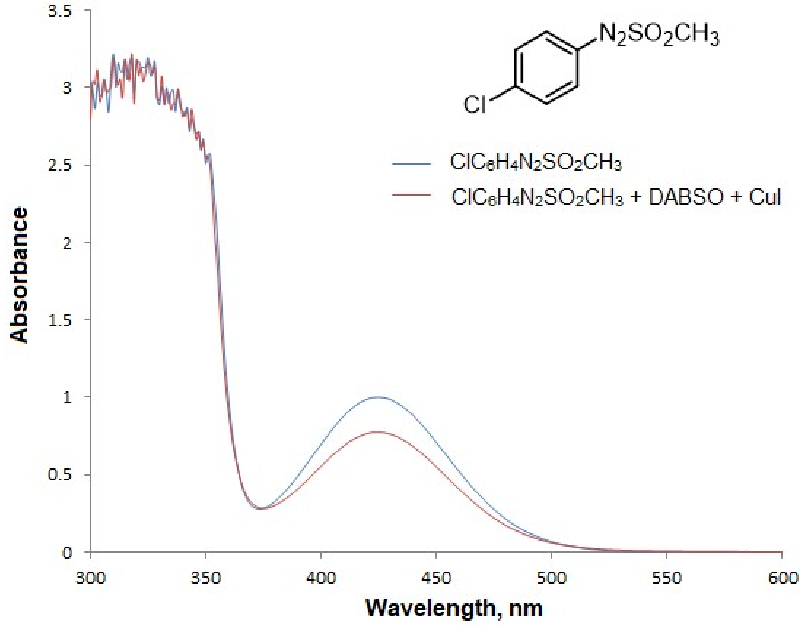

Fig. S3 UV-Vis absorption spectrum of compound **1b** in CH_2Cl_2 and compound **1b** in the presence of DABSO and CuI in CH_2Cl_2

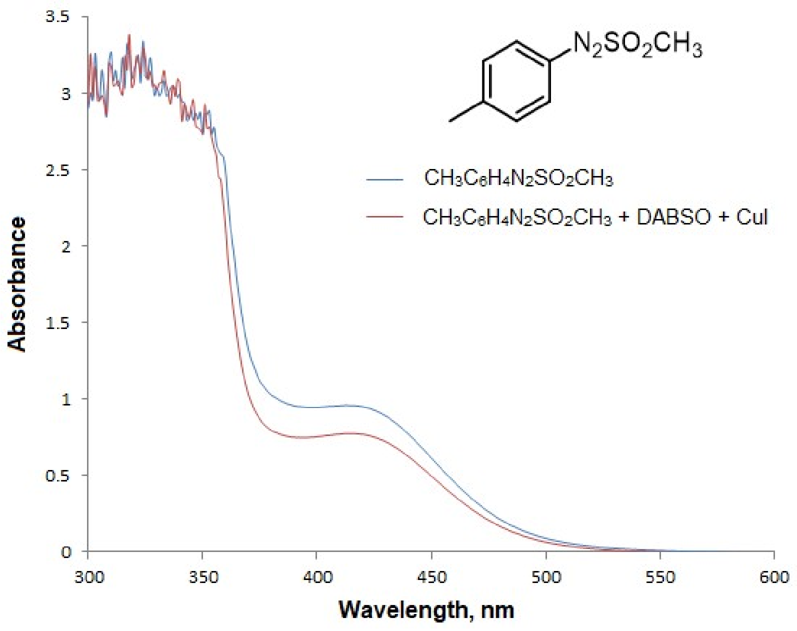

Fig. S4 UV-Vis absorption spectrum of compound **1e** in CH_2Cl_2 and compound **1e** in the presence of DABSO and CuI in CH_2Cl_2

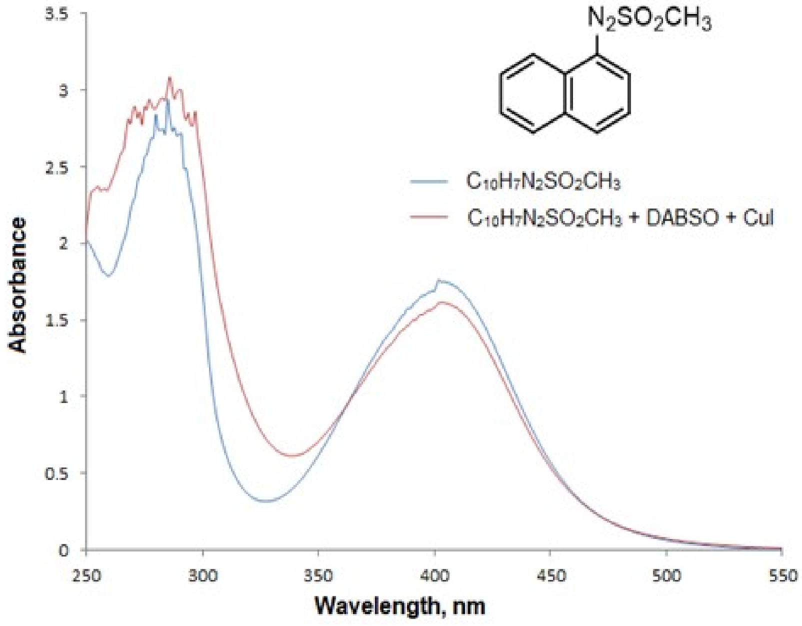

Fig. S5 UV-Vis absorption spectrum of compound **1m** in CH_2Cl_2 and compound **1m** in the presence of DABSO and CuI in CH_2Cl_2

5. General procedure of the synthesis of sulfonic ester compounds

In a typical synthetic procedure, arylazo sulfone compound **1a** (185 mg, 1.0 mmol), DABSO (240 mg, 1.0 mmol), and CuI (19 mg, 0.1 mmol) were added to a mixed solution of dichloromethane (2 mL) and methanol (640 mg, 20 mmol). Hydrochloric acid 37% (100 mg, 1.0 mmol) was dropped into the reaction mixture. The mixture was stirred at room temperature under irradiation by blue LEDs. After 4 hours, the mixture was extracted with 50 mL of CH_2Cl_2 and washed with 50 mL of brine solution. The organic layer was dried by sodium sulfate and concentrated under reduced pressure. The residue was purified using flash column chromatography on silica gel with hexane-EtOAc as the eluent to get the intended product **3a** (146 mg, 85%).

6. Quantum yield measurement

The quantum yield was calculated according to established protocols with minor adjustments.[S7-S10]

The standard ferrioxalate actinometry was used to measure the photon flux. Preparing two following solutions and storing them in the dark:

The 0.15M solution of $K_3Fe(C_2O_4)_3$ (solution A): Dissolving 736.9 mg of $K_3Fe(C_2O_4)_3.3H_2O$ in 10mL of 0.05M H_2SO_4.

The buffered solution of 1,10-phenanthroline (solution B): Dissolving 547 mg 1,10-phenanthroline and 6.6 g of sodium acetate in 60ml 0.5M H_2SO_4.

2.0 mL of solution A was added to reaction tube and stirred. The reaction tube was irradiated by Blue LEDs system at room temperature for 30 seconds. Then 0,35 mL of solution B was added to the reaction tube. The resulting mixture was kept in the dark for 1 hour. 50 µL of mixture solution was diluted to 2.50 mL and then measured absorbance (A) at 510 nm with the

non-irradiation sample as the blank sample. Conversion was calculated according to the Beer-Lambert law:

$$mol\,Fe^{2+} = \frac{V_1 * V_3 * A}{V_2 * l * \varepsilon} = \frac{0.00235 * 0.0025 * 0.734}{0.00005 * 1 * 11100} = 7.77 * 10^{-6}\ (mol)$$

V_1 is total volume of reaction solution (0.002 L solution A + 0.00035 L solution B); V_2 is the volume of the reaction solution carried out to be diluted to determine the ferrous ions (5.10^{-5} L); V_3 is the volume of diluted solution (0.0025 L); *l* is thickness of cuvette (1.0 cm); Molar extinction coefficient (ε) of $Fe(phen)_3^{2+}$ is 11100 L/mol.cm at 510 nm; the measured absorbance value (A) is 0.734.

The photon flux could be calculated according to equation:

$$photon\,flux = \frac{mol\,Fe^{2+}}{\phi * t * f} = \frac{7.77 * 10^{-6}}{1.1 * 30 * 0.97} = 2.43 * 10^{-7}\,einstain.s^{-1}$$

Φ is the quantum yield for standard ferrioxalate actinometry (Φ_{450nm} ~ *1.1*[S11]); t is irradiation time (30s); f is the fraction of light absorbed by the 0.15M ferrioxalate solution (f ~ 0.97 at 450 nm)[S12].

The reaction of sulfonic ester synthesis process was conduct for 4 hours. Therefore, the quantum yield of products was calculated according to the following equation:

$$Quantum\,yeild\,\phi = \frac{mol\,of\,product}{photon\,flux * \ t * f} = \frac{mol\,of\,product}{2.43 * 10^{-7} * 4 * 3600 * 0.97}$$

$$= \frac{mol\,of\,product}{3.4 * 10^{-3}}$$

Quantum yield values are presented in the section on characterization of sulfonic ester compounds below.

7. Characterization of sulfonic ester compounds

Methyl benzenesulfonate (3a)[S1]

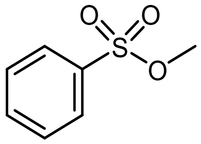

3a was obtained in 85% yield (146.1 mg) according to the general procedure (Hexan/EtOAc, 10:1): yellow oil; ^{1}H NMR (400 MHz, $CDCl_3$) δ 7.73-7.70 (m, 2H), 7.57-7.55 (m, 3H), 3.49 (s, 3H); ^{13}C NMR (100 MHz, $CDCl_3$) δ 144.0, 132.2, 129.1 (2C), 125.4 (2C), 49.7; HRMS (ESI) m/z $(M+H)^+$ calcd for $C_7H_9O_3S$ = 173.0272; found 173.0173; quantum yield $\phi = 0.25$

Methyl 4-chlorobenzenesulfonate (3b)[S1]

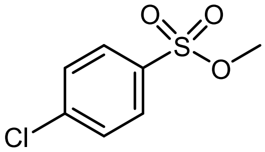

3b was obtained in 60% yield (123.3 mg) according to the general procedure (Hexan/EtOAc, 15:1): colorless oil; ^{1}H NMR (400 MHz, $CDCl_3$) δ 7.66 (d, J = 8.8 Hz, 2H), 7.54 (d, J = 8.8 Hz, 2H), 3.49 (s, 3H); ^{13}C NMR (100 MHz, $CDCl_3$) δ 140.7, 133.7, 129.7 (2C), 129.5 (2C), 56.5; HRMS (ESI) m/z $(M+H)^+$ calcd for $C_7H_8ClO_3S$ = 206.9883; found 206.9885; quantum yield $\phi = 0.17$

Methyl 4-nitrobenzenesulfonate (3c)[S1]

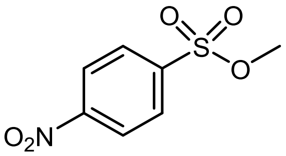

3c was obtained in 56% yield (121.5 mg) according to the general procedure procedure (Hexan/EtOAc, 5:1): orange solid; ^{1}H NMR (400 MHz, $CDCl_3$) δ 8.42 (d, J = 8.8 Hz, 2H), 7.92 (d, J = 8.8 Hz, 2H), 3.56 (s, 3H); ^{13}C NMR (100 MHz, $CDCl_3$) δ 141.1, 129.4 (2C), 126.9, 124.5 (2C), 57.0; HRMS (ESI) m/z $(M+H)^+$ calcd for $C_7H_8NO_5S$ = 218.0123; found 218.0121; quantum yield $\phi = 0.16$

Methyl 4-acetylbenzenesulfonate (3d)[S2]

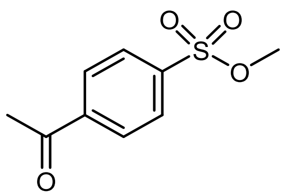

3d was obtained in 63% yield (134.8 mg) according to the general procedure (Hexan/EtOAc, 5:1): light yellow oil; ^1H NMR (400 MHz, $CDCl_3$) δ 8.13 (d, J = 8.4 Hz, 2H), 7.83 (d, J = 8.4 Hz, 2H), 3.52 (s, 3H), 2.66 (s, 3H); ^{13}C NMR (100 MHz, $CDCl_3$) δ 197.1, 148.4, 139.9, 128.9 (2C), 125.9 (2C), 50.2, 26.9; HRMS (ESI) m/z $(M+H)^+$ calcd for $C_9H_{11}O_4S$ = 215.0378; found 215.0375; quantum yield $\phi = 0.18$

Methyl 4-methylbenzenesulfonate (3e)[S2]

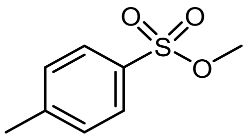

3e was obtained in 82% yield (152.4 mg) according to the general procedure (Hexan/EtOAc, 15:1): yellow oil; ^1H NMR (400 MHz, $CDCl_3$) δ 7.60 (d, J = 8.8 Hz, 2H), 7.35 (d, J = 8.8 Hz, 2H), 3.47 (s, 3H), 2.43 (s, 3H); ^{13}C NMR (100 MHz, $CDCl_3$) δ 142.8, 141.1, 129.7 (2C), 125.4 (2C), 49.4, 21.5; HRMS (ESI) m/z $(M+H)^+$ calcd for $C_8H_{11}O_3S$ = 187.0429; found 187.0428; quantum yield $\phi = 0.24$

Methyl 4-(tert-butyl)benzenesulfonate (3f)[S2]

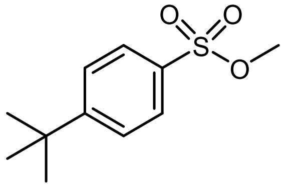

3f was obtained in 78% yield (177.7 mg) according to the general procedure (Hexan/EtOAc, 15:1): light yellow oil; ^1H NMR (400 MHz, $CDCl_3$) δ 7.64 (d, J = 8.8 Hz, 2H), 7.57 (d, J = 8.8 Hz, 2H), 3.50 (s, 3H), 1.35 (s, 9H); ^{13}C NMR (100 MHz, $CDCl_3$) δ 155.9, 140.9, 126.1 (2C), 125.2 (2C), 49.7, 35.1, 31.2 (3C); HRMS (ESI) m/z $(M+H)^+$ calcd for $C_{11}H_{17}O_3S$ = 229.0898; found 229.0899; quantum yield $\phi = 0.23$

Methyl 4-methoxybenzenesulfonate (3g)[S1]

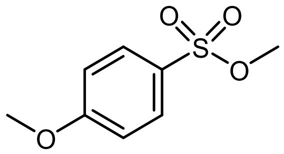

3g was obtained in 81% yield (164.4 mg) according to the general procedure (Hexan/EtOAc, 10:1): light yellow oil; ^{1}H NMR (400 MHz, $CDCl_3$) δ 7.85 (d, J = 8.8 Hz, 2H), 7.03-7.00 (dd, J = 8.8 Hz, 2H), 3.89 (s, 3H), 3.72 (s, 3H); ^{13}C NMR (100 MHz, $CDCl_3$) δ 163.8, 130.3 (2C), 126.6, 114.5 (2C), 56.0, 55.7; HRMS (ESI) m/z $(M+H)^+$ calcd for $C_8H_{11}O_4S$ = 203.0378; found 203.0377; quantum yield ϕ = *0.24*

Methyl 4-(benzyloxy)benzenesulfonate (3h):

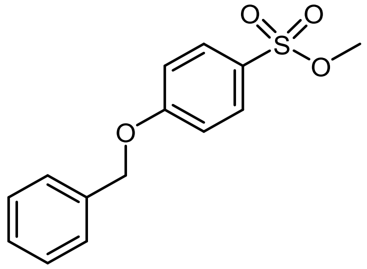

3h was obtained in 67% yield (186.1 mg) according to the general procedure (Hexan/EtOAc, 10:1): yellow oil; ^{1}H NMR (400 MHz, $CDCl_3$) δ 7.64 (d, J = 6.8, 2H), 7.44-7.35 (m, 5H), 7.11-7.09 (d, J = 7.2, 2H), 5.13 (s, 2H), 3.47 (s, 3H); ^{13}C NMR (100 MHz, $CDCl_3$) δ 161.9, 136.1, 135.9, 128.7 (2C), 128.3, 127.5 (2C), 127.3 (2C), 115.3 (2C), 70.3, 49.3; HRMS (ESI) m/z $(M+H)^+$ calcd for $C_{14}H_{15}O_4S$ = 279.0691; found 279.0693; quantum yield ϕ = *0.20*

Methyl 3-chlorobenzenesulfonate (3i):

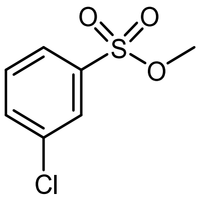

3i was obtained in 51% yield (105.0 mg) according to the general procedure (Hexan/EtOAc, 15:1): colorless oil; ^{1}H NMR (400 MHz, $CDCl_3$) δ 7.71 (s, 1H), 7.59 (d, J = 7.2 Hz, 1H), 7.55-7.47 (m, 2H), 3.51 (s, 3H); ^{13}C NMR (100 MHz, $CDCl_3$) δ 145.9, 135.6, 132.4, 130.4, 125.6, 123.7; HRMS (ESI) m/z $(M+H)^+$ calcd for $C_7H_8ClO_3S$ = 206.9883; found 206.9885; quantum yield ϕ = *0.15*

Methyl 3-methylbenzenesulfonate (3j)[S2]:

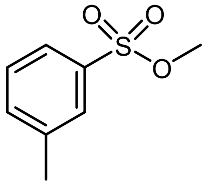

3j was obtained in 73% yield (135.6 mg) according to the general procedure (Hexan/EtOAc, 10:1): light orange oil; ^{1}H NMR (400 MHz, $CDCl_3$) δ 7.51 (t, J = 6.4 Hz, 2H), 7.43 (t, J = 6.4 Hz, 1H), 7.37 (d, J = 7.2 Hz 1H), 3.49 (s, 3H), 2.44 (s, 3H); ^{13}C NMR (100 MHz, $CDCl_3$) δ 143.9, 139.3, 133.0, 128.9, 125.7, 122.5, 49.7, 21.4; HRMS (ESI) m/z $(M+H)^+$ calcd for $C_8H_{11}O_3S$ = 187.0429; found 187.0428; quantum yield ϕ = *0.21*

Methyl 3,5-dichlorobenzenesulfonate (3k)

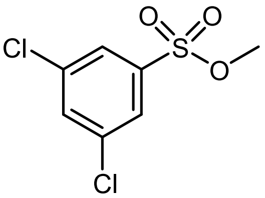

3k was obtained in 49% yield (117.5 mg) according to the general procedure (Hexan/EtOAc, 15:1): yellow oil; ^{1}H NMR (400 MHz, $CDCl_3$) δ 7.58 (d, J = 1.6 Hz, 2H), 7.54 (d, J = 1.6 Hz, 1H), 3.53 (s, 3H); ^{13}C NMR (100 MHz, $CDCl_3$) δ 147.3, 136.3, 132.3 (2C), 124.0 (2C), 50.3; HRMS (ESI) m/z $(M+H)^+$ calcd for $C_7H_7Cl_2O_3S$ = 240.9493; found 240.9495; quantum yield ϕ = *0.14*

Methyl 3,5-dimethylbenzenesulfonate (3l)[S2]:

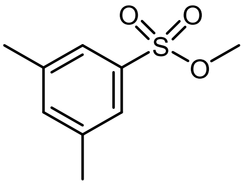

3l was obtained in 75% yield (150.0 mg) according to the general procedure (Hexan/EtOAc, 10:1): yellow oil; ^{1}H NMR (400 MHz, $CDCl_3$) δ 7.31 (s, 2H), 7.17 (s, *1*H), 3.49 (s, 3H), 2.39 (s, 6H); ^{13}C NMR (100 MHz, $CDCl_3$) δ 143.8, 139.1, 133.9 (2C), 122.8 (2C), 49.9, 21.3 (2C); HRMS (ESI) m/z $(M+H)^+$ calcd for $C_9H_{13}O_3S$ = 201.0585; found 201.0586; quantum yield ϕ = *0.22*

Methyl 4-naphthalene-1-sulfonate (3m)[S1]:

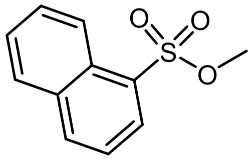

3m was obtained in 75% yield (166.5 mg) according to the general procedure (Hexan/EtOAc, 15:1): violet oil; ^{1}H NMR (400 MHz, $CDCl_3$) δ 8.31 (d, J = 7.6 Hz, 1H), 8.17 (d, J = 7.2 Hz, 1H), 8.06 (d, J = 8.0 Hz, 1H), 7.97 (d, J = 7.6 Hz, 1H), 7.67-7.58 (m, 3H), 3.45 (s, 3H); ^{13}C NMR (100 MHz, $CDCl_3$) δ 138.4, 133.8, 132.9, 129.5, 128.8, 127.6, 126.8, 124.8 (2C), 122.4, 49.6; HRMS (ESI) m/z $(M+H)^+$ calcd for $C_{11}H_{11}O_3S$ = 223.0429; found 223.0426; quantum yield ϕ *= 0.22*

Methyl benzo[*d*][1,3]dioxole-5-sulfonate (3n):

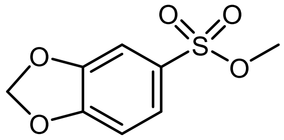

3n was obtained in 83% yield (179.0 mg) according to the general procedure (Hexan/EtOAc, 5:1): light yellow oil; ^{1}H NMR (400 MHz, $CDCl_3$) δ 7.23 (d, J = 7.6 Hz, 1H), 7.16 (s, *1*H), 6.95 (d, J = 7.6 Hz, 1H), 6.07 (s, 2H), 3.48 (s, 3H); ^{13}C NMR (100 MHz, $CDCl_3$) δ 151.1, 148.5, 137.7, 120.6, 108.6, 105.4, 102.0, 42.3; HRMS (ESI) m/z $(M+H)^+$ calcd for $C_8H_9O_5S$ = 217.0171; found 217.0172; quantum yield ϕ *= 0.24*

Methyl 4-(N-(4,6-dimethylpyrimidin-2-yl)sulfamoyl)benzenesulfonate (3o):

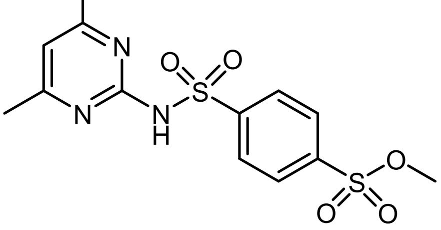

3o was obtained in 49% yield (174.8 mg) according to the general procedure (Hexan/EtOAc, 1:2): light yellow oil; ^{1}H NMR (400 MHz, $CDCl_3$) δ 8.37 (d, J = 8.8 Hz, 2H), 7.88 (d, J = 8.8 Hz, 2H), 7.09 (d, J = 8.4 Hz, 2H), 6.80 (s, 1H), 3.55 (s, 3H), 2.51 (s, 6H); ^{13}C NMR (100 MHz,

$CDCl_3$) δ 168.4, 155.5, 148.6, 143.4, 129.6 (2C), 125.7 (2C), 115.1, 50.5, 23.5; HRMS (ESI) m/z $(M+H)^+$ calcd for $C_{13}H_{16}N_3O_5S_2$ = 358.0531; found 358.0533; quantum yield ϕ = *0.14*

Ethyl benzenesulfonate (4a)[S3]

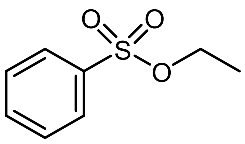

4a was obtained in 79% yield (146.9 mg) according to the general procedure (Hexan/EtOAc, 10:1): light yellow oil; ^{1}H NMR (400 MHz, $CDCl_3$) δ 7.93-7.91 (m, 2H), 7.67-7.63 (m, 1H), 7.58-7.54 (m, 2H), 4.14 (q, J = 8.8 Hz, 2H), 1.31 (t, J = 8.8 Hz, 3H); ^{13}C NMR (100 MHz, $CDCl_3$) δ 136.4, 133.7, 129.2 (2C), 127.8 (2C), 67.0, 14.8; HRMS (ESI) m/z $(M+H)^+$ calcd for $C_8H_{11}O_3S$ = 187.0429; found 187.0426; quantum yield ϕ = *0.23*

Butyl benzenesulfonate (4b)[S4]

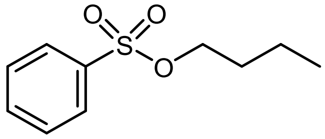

4b was obtained in 67% yield (143.3 mg) according to the general procedure (Hexan/EtOAc, 10:1): light yellow oil; ^{1}H NMR (400 MHz, $CDCl_3$) δ 7.92 (d, J = 7.2 Hz, 2H), 7.65 (t, J = 7.2 Hz, 1H), 7.56 (t, J = 7.6 Hz, 2H), 4.06 (t, J = 6.4 Hz, 2H), 1.67-1.59 (m, 2H), 1.39-1.31 (m, 2H), 0.86 (t, J = 7.2 Hz); ^{13}C NMR (100 MHz, $CDCl_3$) δ 136.3, 133.6, 129.2 (2C), 127.8 (2C), 70.6, 30.8, 18.6, 13.4; HRMS (ESI) m/z $(M+H)^+$ calcd for $C_{10}H_{15}O_3S$ = 215.0742; found 215.0743; quantum yield ϕ = *0.20*

Heptyl benzenesulfonate (4c):

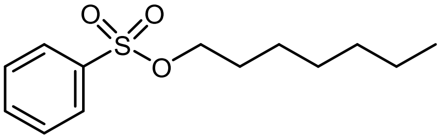

4c was obtained in 61% yield (156.1 mg) according to the general procedure (Hexan/EtOAc, 15:1): colorless oil; ^{1}H NMR (400 MHz, $CDCl_3$) δ 7.92 (d, J = 7.2 Hz, 2H), 7.65 (t, J = 7.2 Hz, 1H), 7.55 (t, J = 7.6 Hz, 2H), 4.05 (t, J = 6.8 Hz, 2H), 1.68-1.61 (m, 2H), 1.30-1.22 (m, 8H),

0.86 (t, J = 6.4 Hz, 3H); ^{13}C NMR (100 MHz, $CDCl_3$) δ 136.3, 133.6, 129.2 (2C), 127.8 (2C), 71.0, 31.6, 28.8, 28.6, 25.3, 22.5, 14.0; HRMS (ESI) m/z $(M+H)^+$ calcd for $C_{13}H_{21}O_3S$ = 257.1211; found 257.1214; quantum yield ϕ = *0.18*

Isobutyl benzenesulfonate (4d)[S6]

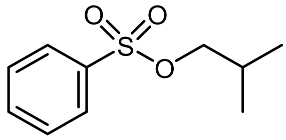

4d was obtained in 68% yield (145.5 mg) according to the general procedure (Hexan/EtOAc, 15:1): colorless oil; ^{1}H NMR (400 MHz, $CDCl_3$) δ 7.92 (d, J = 7.6 Hz, 2H), 7.65 (t, J = 7.2 Hz, 1H), 7.55 (t, J = 8.0 Hz, 2H), 3.83 (d, J = 6.8 Hz, 2H), 1.95 (m, 1H), 0.90 (d, J = 6.8, 6H) ; ^{13}C NMR (100 MHz, $CDCl_3$) δ 136.3, 133.6, 129.2 (2C), 127.8 (2C), 76.5, 28.1, 18.6 (2C); HRMS (ESI) m/z $(M+H)^+$ calcd for $C_{10}H_{15}O_3S$ = 215.0742; found 215.0743; quantum yield ϕ = *0.20*

Benzyl benzenesulfonate (4e):

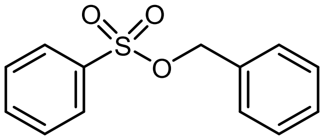

4e was obtained in 73% yield (106.6 mg) according to the general procedure (Hexan/EtOAc, 15:1): colorless oil; ^{1}H NMR (400 MHz, $CDCl_3$) δ 7.79-7.77 (m, 2H), 7.59-7.57 (m, 3H), 7.37-7.34 (m, 3H), 7.31-7.29 (m, 2H), 5.09 (d, 1H), 4.62 (d, 1H); ^{13}C NMR (100 MHz, $CDCl_3$) δ 144.6, 135.5, 132.2, 129.1 (2C), 128.6 (2C), 128.5 (2C), 127.0, 125.4 (2C), 66.0; HRMS (ESI) m/z $(M+H)^+$ calcd for $C_{13}H_{13}O_3S$ = 249.0585; found 249.0587; quantum yield ϕ = *0.21*

Cyclohexyl benzenesulfonate (4f)[S5]

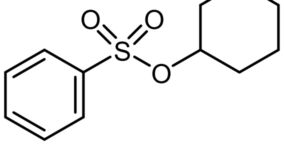

4f was obtained in 65% yield (155.8 mg) according to the general procedure (Hexan/EtOAc, 10:1): light yellow solid; ^{1}H NMR (400 MHz, $CDCl_3$) δ 7.75-7.73 (m, 2H), 7.56-7.54 (m, 3H),

4.37 (m, 1H), 1.81-1.27 (m, 10H); ^{13}C NMR (100 MHz, $CDCl_3$) δ 145.9, 131.9, 128.9 (2C), 125.1 (2C), 78.1, 33.7 (2C), 25.2, 23.9 (2C); HRMS (ESI) m/z $(M+H)^+$ calcd for $C_{12}H_{17}O_3S$ = 241.0898; found 241.0896; quantum yield $\phi = 0.19$

Tert-butyl benzenesulfonate (4g):

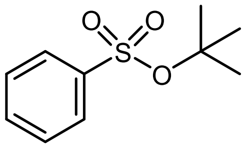

4g was obtained in 68% yield (145.5 mg) according to the general procedure (Hexan/EtOAc, 10:1): orange oil; ^1H NMR (400 MHz, $CDCl_3$) δ 7.68 (d, J = 6.4 Hz, 2H), 7.52-7.50 (m, 3H), 1.56 (s, 9H); ^{13}C NMR (100 MHz, $CDCl_3$) δ 134.0, 131.6, 128.9 (2C), 124.9 (2C), 82.9, 29.9 (3C); HRMS (ESI) m/z $(M+H)^+$ calcd for $C_{10}H_{15}O_3S$ = 215.0742; found 215.0741; quantum yield $\phi = 0.20$

Tert-butyl 4-nitrobenzensulfonate (4h):

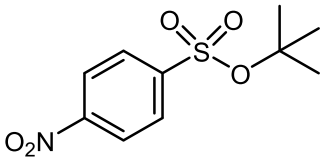

4h was obtained in 45% yield (116.4 mg) according to the general procedure (Hexan/EtOAc, 10:1): light yellow solid, ^1H NMR (400 MHz, $CDCl_3$) δ 8.38 (d, J = 8.8 Hz, 2H), 7.87 (d, J = 8.8 Hz, 2H), 1.58 (s, 9H); ^{13}C NMR (100 MHz, $CDCl_3$) δ 152.9, 149.8, 126.3 (2C), 124.2 (2C), 84.4, 29.9 (3C); HRMS (ESI) m/z $(M+H)^+$ calcd for $C_{10}H_{14}NO_5S$ = 260.0593; found 260.0594; quantum yield $\phi = 0.13$

Tert-butyl 4-methylbenzenesulfonate (4i)[S2]

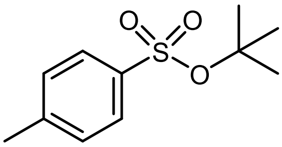

4i was obtained in 73% yield (166.3 mg) according to the general procedure (Hexan/EtOAc, 10:1): light yellow oil; ^1H NMR (400 MHz, $CDCl_3$) δ 7.57 (d, J = 8.0 Hz, 2H), 7.32 (d, J = 8.0 Hz, 2H), 2.41 (s, 3H), 1.55 (s, 9H; ^{13}C NMR (100 MHz, $CDCl_3$) δ 143.7, 142.1, 129.6 (2C),

124.9 (2C), 82.6, 29.9 (3C), 21.5; HRMS (ESI) m/z $(M+H)^+$ calcd for $C_{11}H_{17}O_3S$ = 229.0898; found 229.0895; quantum yield $\phi = 0.21$

Tert-butyl 4-ethylbenzenesulfonate (4j):

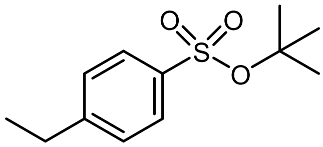

4j was obtained in 76% yield (183.8 mg) according to the general procedure (Hexan/EtOAc, 10:1): light yellow oil; ^{1}H NMR (400 MHz, $CDCl_3$) δ 7.59 (d, J = 8.4 Hz, 2H), 7.34 (d, J = 8.4 Hz 2H), 2.71 (q, J = 7.6 Hz, 2H), 1.55 (s, 9H), 1.25 (t, J = 7.6 Hz, 3H); ^{13}C NMR (100 MHz, $CDCl_3$) δ 148.3, 143.9, 128.5 (2C), 125.0 (2C), 82.6, 29.9 (3C), 28.8, 15.4; HRMS (ESI) m/z $(M+H)^+$ calcd for $C_{12}H_{19}O_3S$ = 243.1055; found 243.1056; quantum yield $\phi = 0.22$

Tert-butyl 4-methoxybenzenesulfonate (4k):

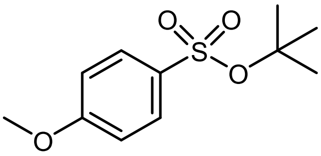

4k was obtained in 71% yield (173.2 mg) according to the general procedure (Hexan/EtOAc, 10:1): light yellow oil; ^{1}H NMR (400 MHz, $CDCl_3$) δ 7.61 (d, J = 8.8 Hz, 2H), 7.01 (d, J = 8.8 Hz, 2H), 3.85 (s, 3H), 1.54 (s, 9H); ^{13}C NMR (100 MHz, $CDCl_3$) δ 162.2, 138.3, 126.7 (2C), 114.3 (2C), 82.4, 55.5, 29.9 (3C); HRMS (ESI) m/z $(M+H)^+$ calcd for $C_{11}H_{17}O_4S$ = 245.0848; found 245.0847; quantum yield $\phi = 0.21$

Tert-butyl 4-phenethylbenzenesulfonate (4l):

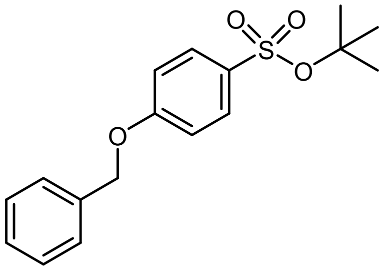

4l was obtained in 59% yield (188.7 mg) according to the general procedure (Hexan/EtOAc,

10:1): yellow oil; ^{1}H NMR (400 MHz, $CDCl_3$) δ 7.61 (d, J = 8.8 Hz, 2H), 7.43-7.34 (m, 5H), 7.09 (d, J = 8.8 Hz, 2H), 5.11 (s, 2H), 1.55 (s, 9H); ^{13}C NMR (100 MHz, $CDCl_3$) δ 161.4, 138.6, 136.3, 128.7 (2C), 128.2, 127.5 (2C), 126.7 (2C), 82.5, 70.3, 29.9 (3C); HRMS (ESI) m/z $(M+H)^+$ calcd for $C_{17}H_{21}O_4S$ = 321.1161; found 321.1164; quantum yield ϕ = *0.17*

References

S1 B. K. G. Bhatthula, J. R. Kanchani, V. R. Arava and S. M. C. Subbarao, *Synth. Commun.*, 2020, **50**, 3133-3148.

S2 Y. Wang, L. Deng, Y. Deng and J. Han, *J. Org. Chem.*, **83**, 4674-4680.

S3 L. Jing, X. Yu, M. Guan, X. Wu, Q. Wang and Y. Wu, *Chem. Res. Chin. Univ.*, 2018, **34**, 191-196.

S4 J. Chandra, R. Chaudhuri, S. R. Manne, S. Mondal and B. Mandal, *ChemistrySelect*, 2017, **2**, 8471-8477.

S5 F. Tamaddon, A. Nasiri and S. Farokhi, *Catal. Commun.*, 2011, **12**, 1477-1482.

S6 T. Guo, Y. Shi, L. Zheng, F. Feng, F. Zheng and W. Liu, *J. Chromatogr. A*, 2014, **1355**, 73-79.

S7 M. A. Cismesia and T. P. Yoon, *Chem. Sci.*, 2015, **6**, 5426-5434.

S8 X. Huang, T. R. Quinn, K. Harms, R. D. Webster, L. Zhang, O. Wiest and E. Meggers, *J. Am. Chem. Soc.*, 2017, **139**, 9120-9123.

S9 D. Wang, F. Loose, P. J. Chirik and R. R. Knowles, *J. Am. Chem. Soc.*, 2019, **141**, 4795-4799.

S10 X. Huang, X. Li, X. Xie, K. Harms, R. Riedel and E. Meggers, *Nat. Commun.*, 2017, **8**, 1-8.

S11 Hatchard, C. G.; Parker, C. A. A new sensitive chemical actinometer II. Potassium ferrioxalate as a standard chemical actinometer. *Proceedings of the Royal Society of London. Series A, Mathematical and Physical Sciences.* **1956**, *235*, 518-536

S12 Cismesia, M. A.; Yoon, T. P. Characterizing chain processes in visible light photoredox catalysis. *Chem. Sci*. **2015**, *6*, 5426-5434

Methyl benzenesulfonate (3a)

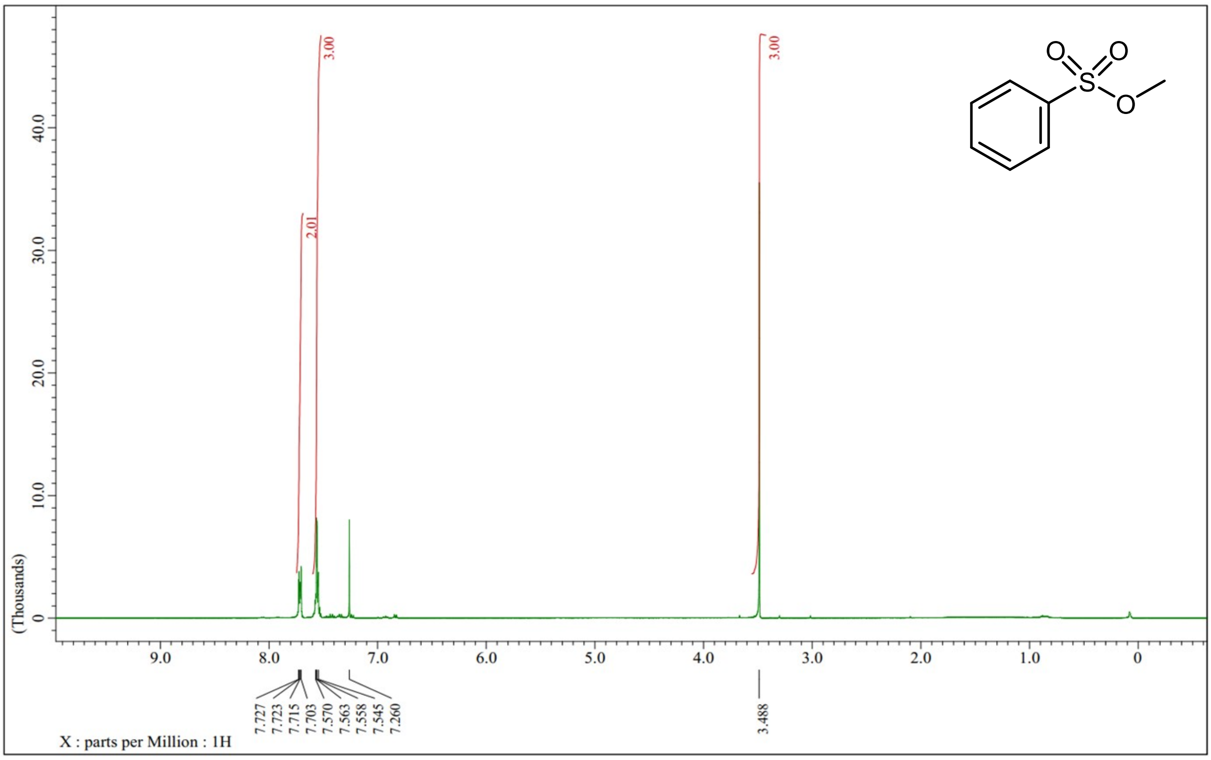

^{1}H NMR spectrum of methyl benzenesulfonate (3a)

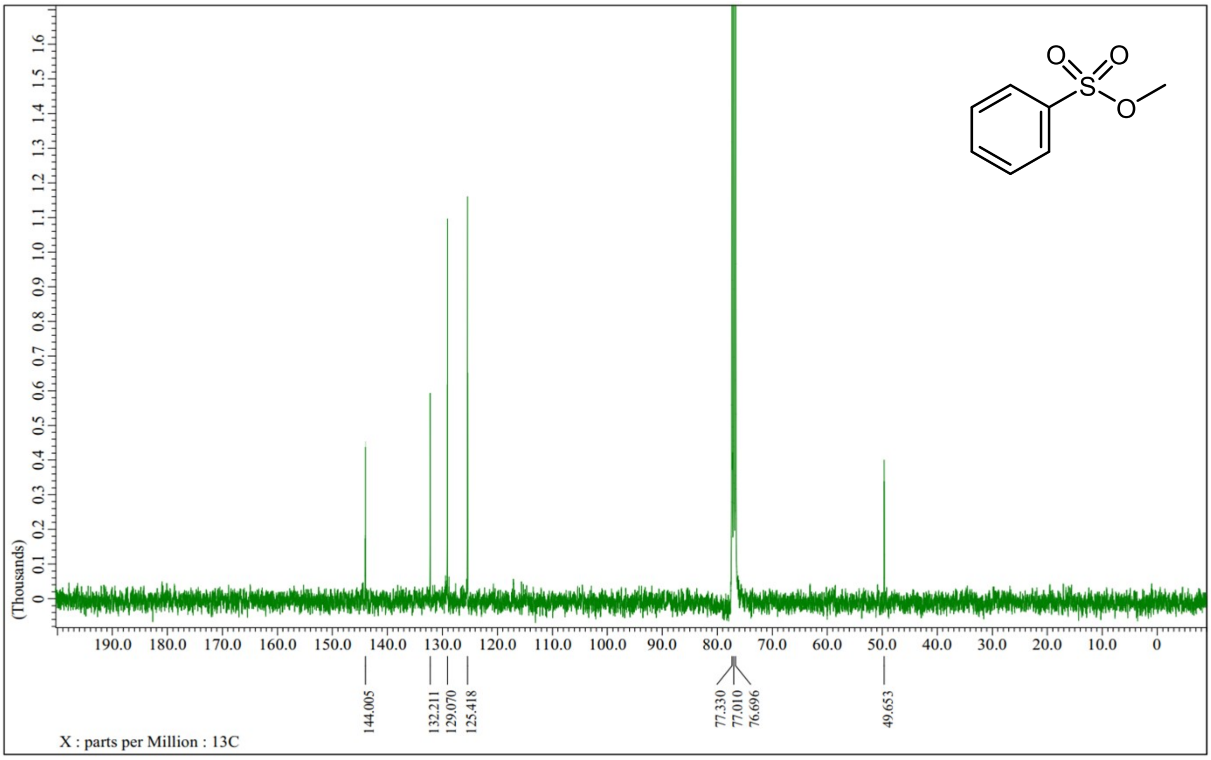

^{13}C NMR spectrum of methyl benzenesulfonate (3a)

Methyl 4-chlorobenzenesulfonate (3b)

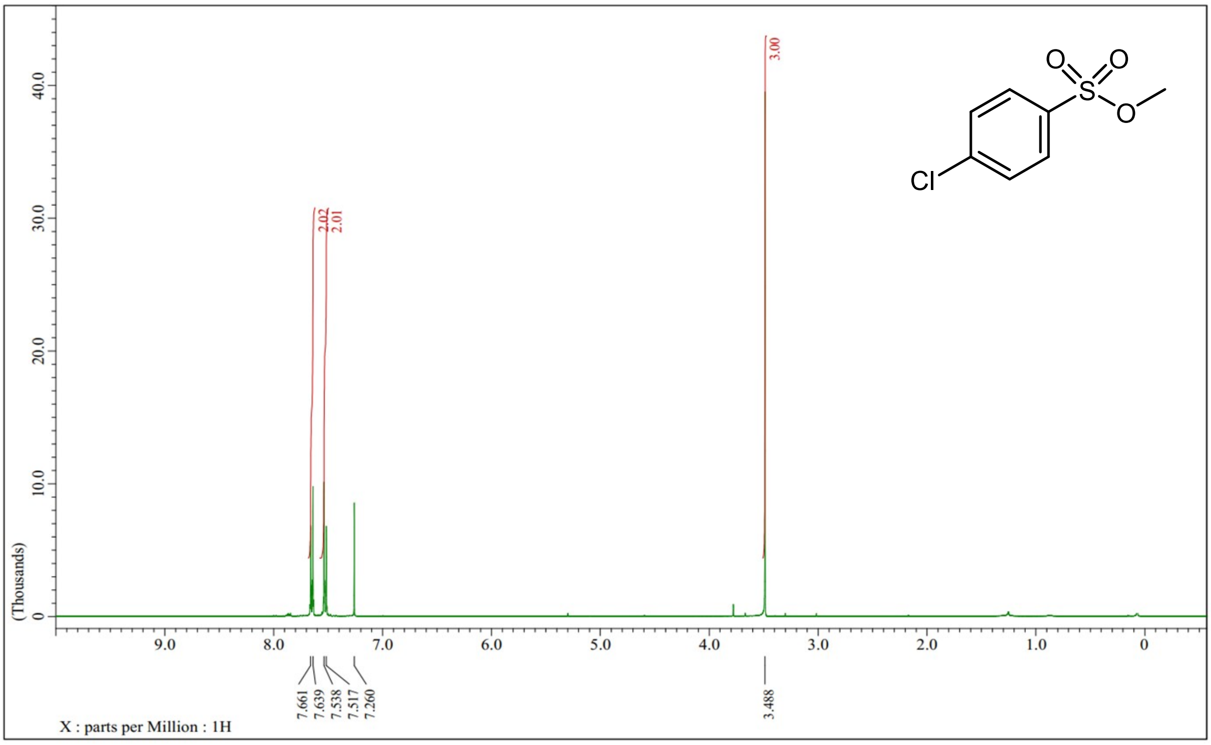

^{1}H NMR spectrum of methyl 4-chlorobenzenesulfonate (3b)

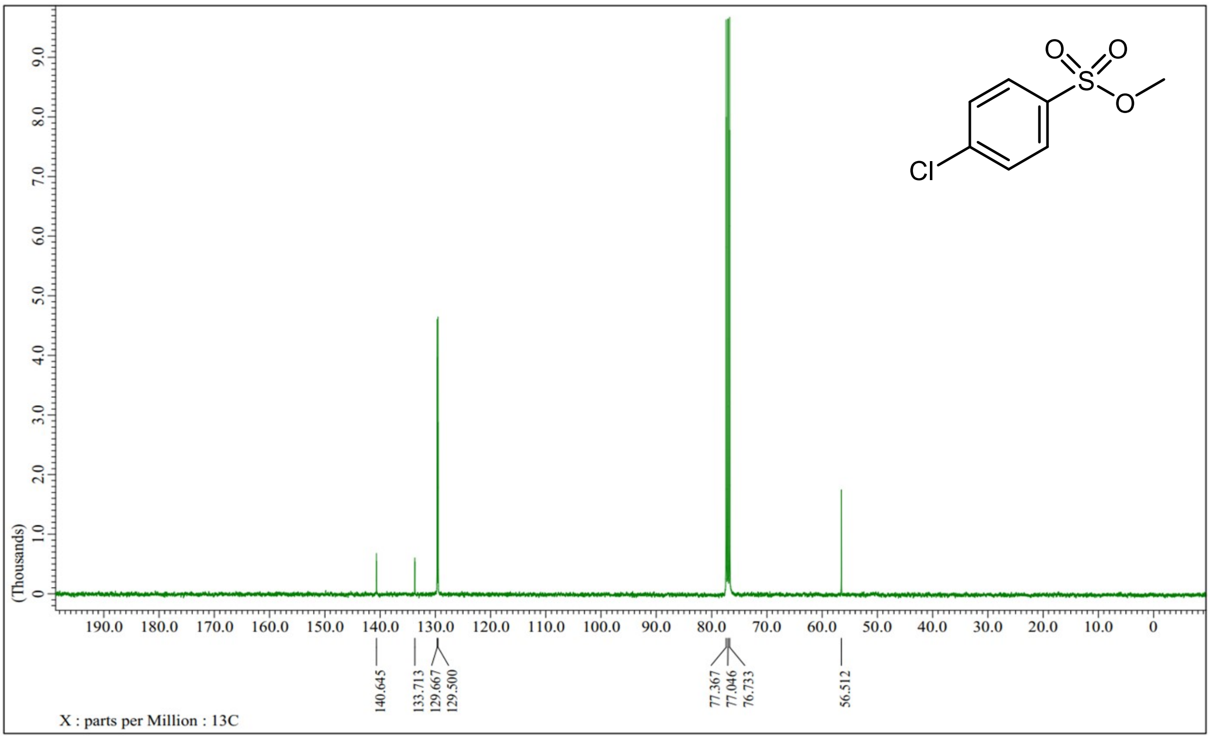

^{13}C NMR spectrum of methyl 4-chlorobenzenesulfonate (3b)

Methyl 4-nitrobenzenesulfonate (3c)

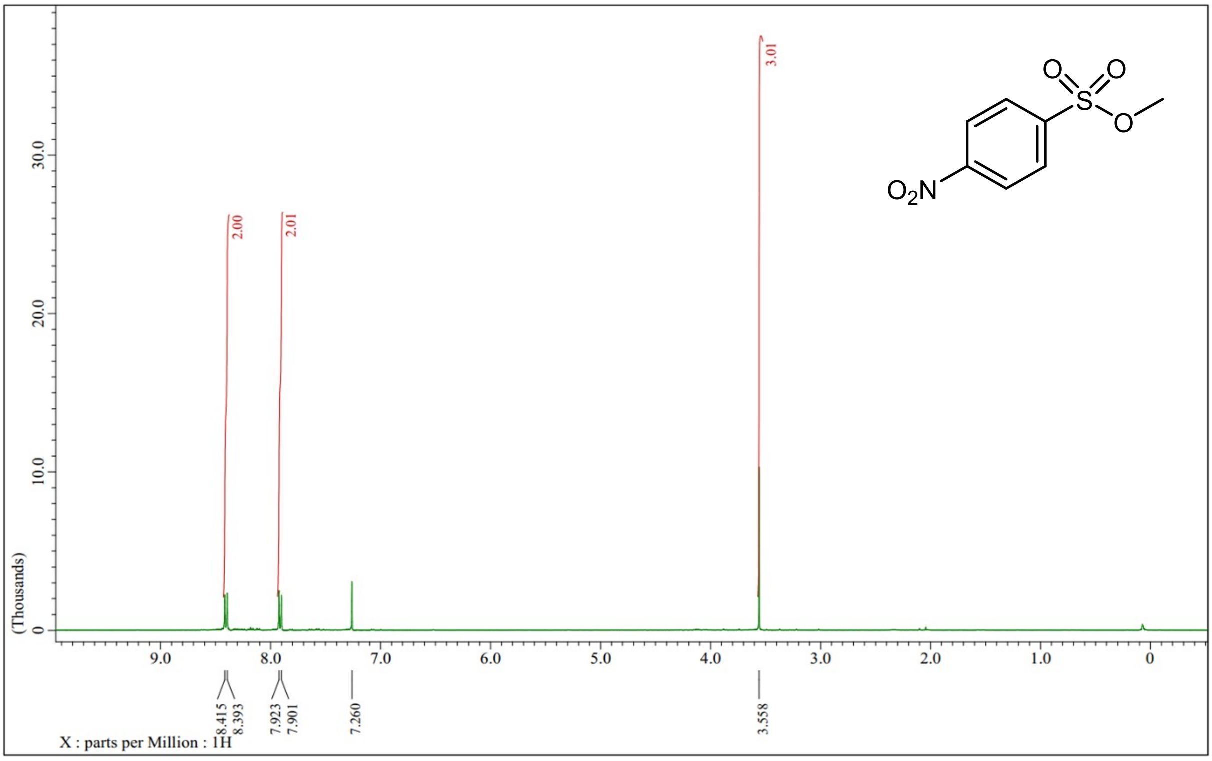

^{1}H NMR spectrum of methyl 4-nitrobenzenesulfonate (3c)

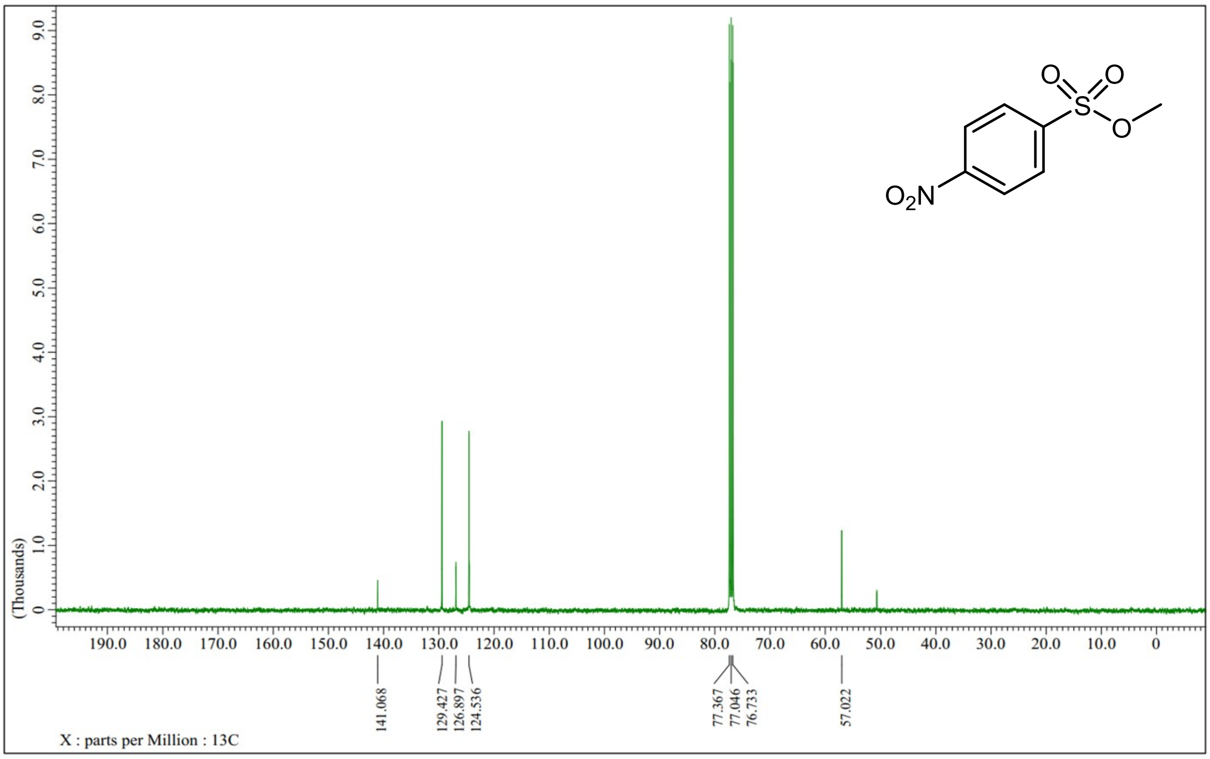

^{13}C NMR spectrum of methyl 4-nitrobenzenesulfonate (3c)

Methyl 4-acetylbenzenesulfonate (3d)

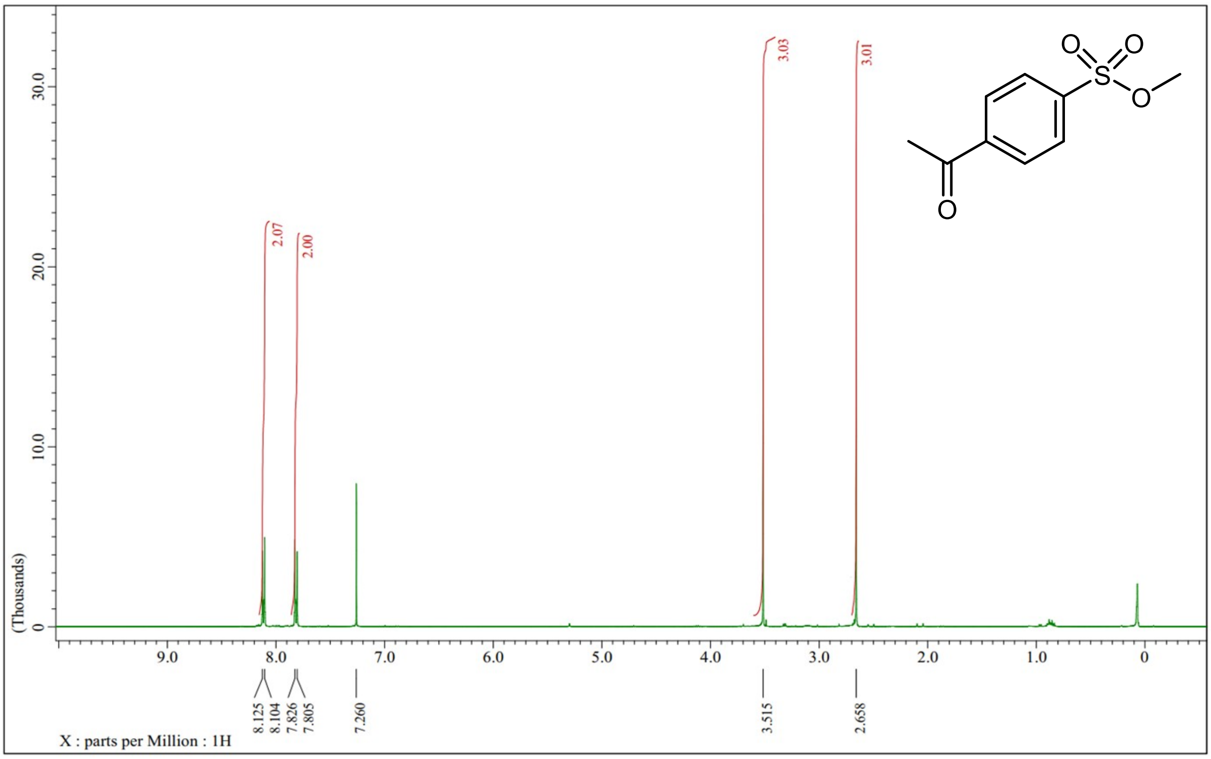

^{1}H NMR spectrum of methyl 4-acetylbenzenesulfonate (3d)

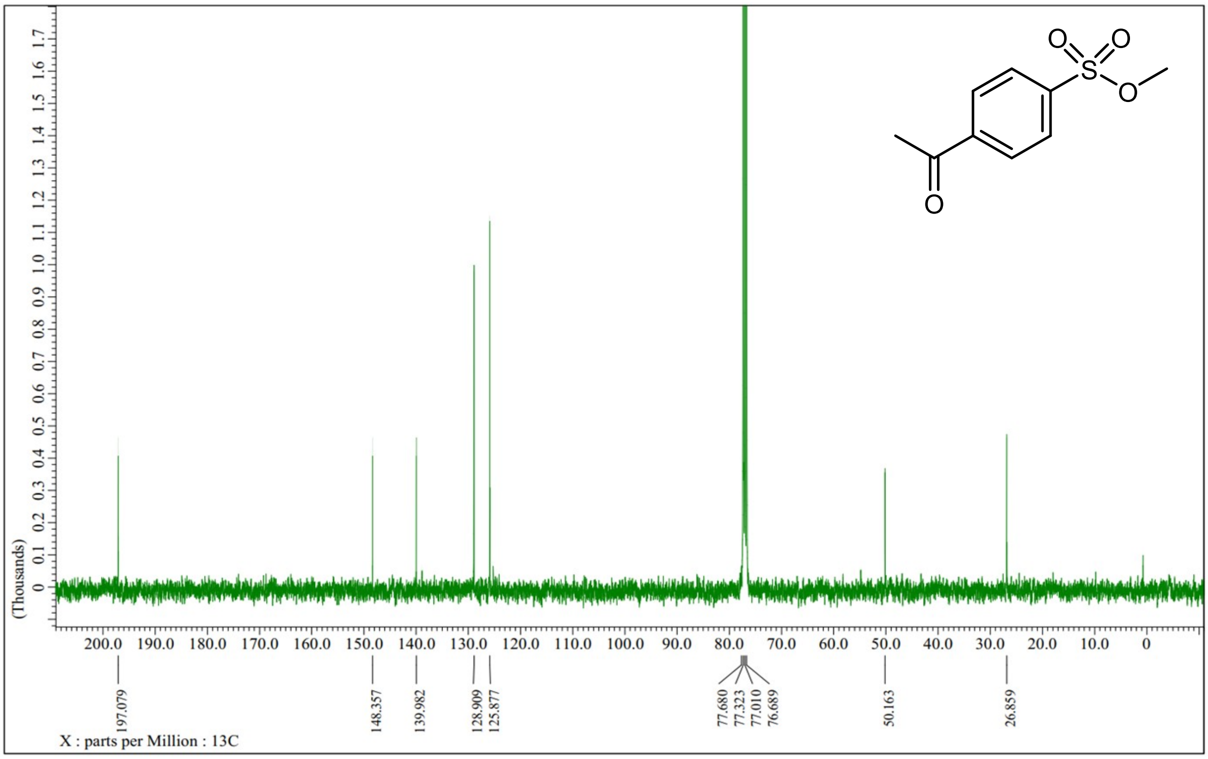

^{13}C NMR spectrum of methyl 4-acetylbenzenesulfonate (3d)

Methyl 4-methylbenzenesulfonate (3e)

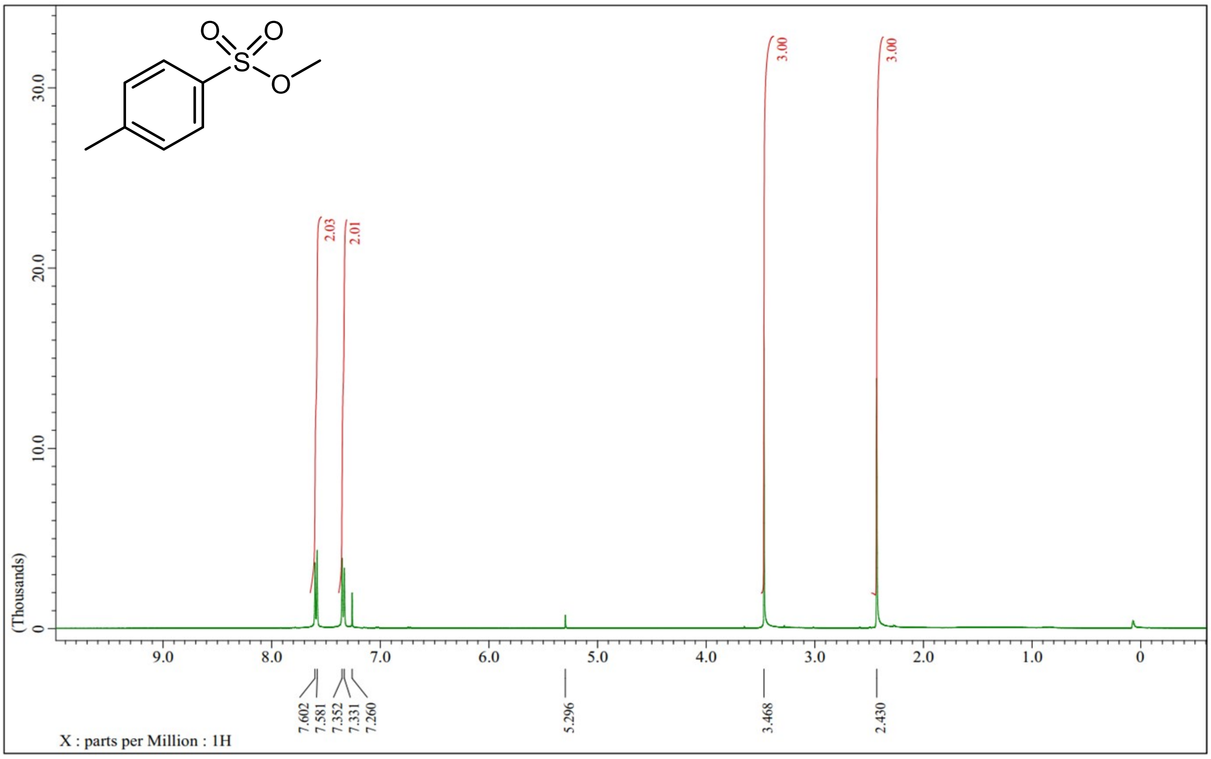

^{1}H NMR spectrum of methyl 4-methylbenzenesulfonate (3e)

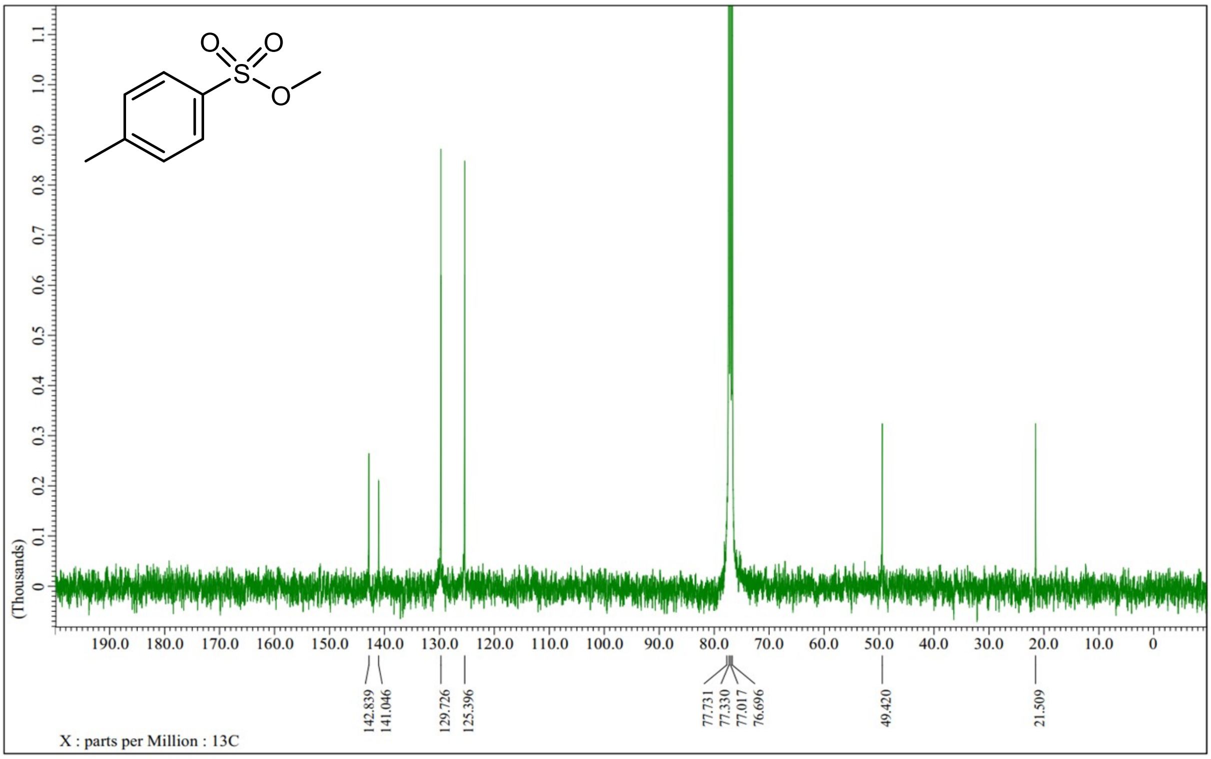

^{13}C NMR spectrum of methyl 4-methylbenzenesulfonate (3e)

Methyl 4-(tert-butyl)benzenesulfonate (3f)

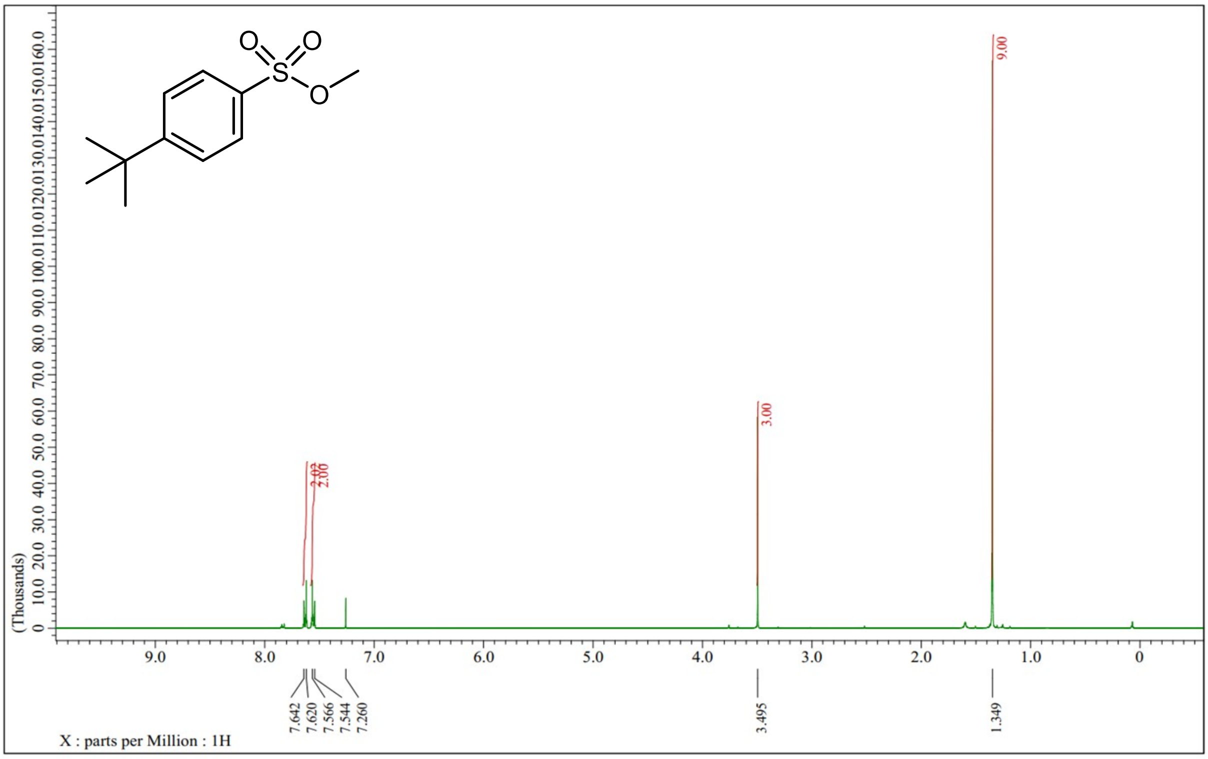

^{1}H NMR spectrum of methyl 4-(tert-butyl)benzenesulfonate (3f)

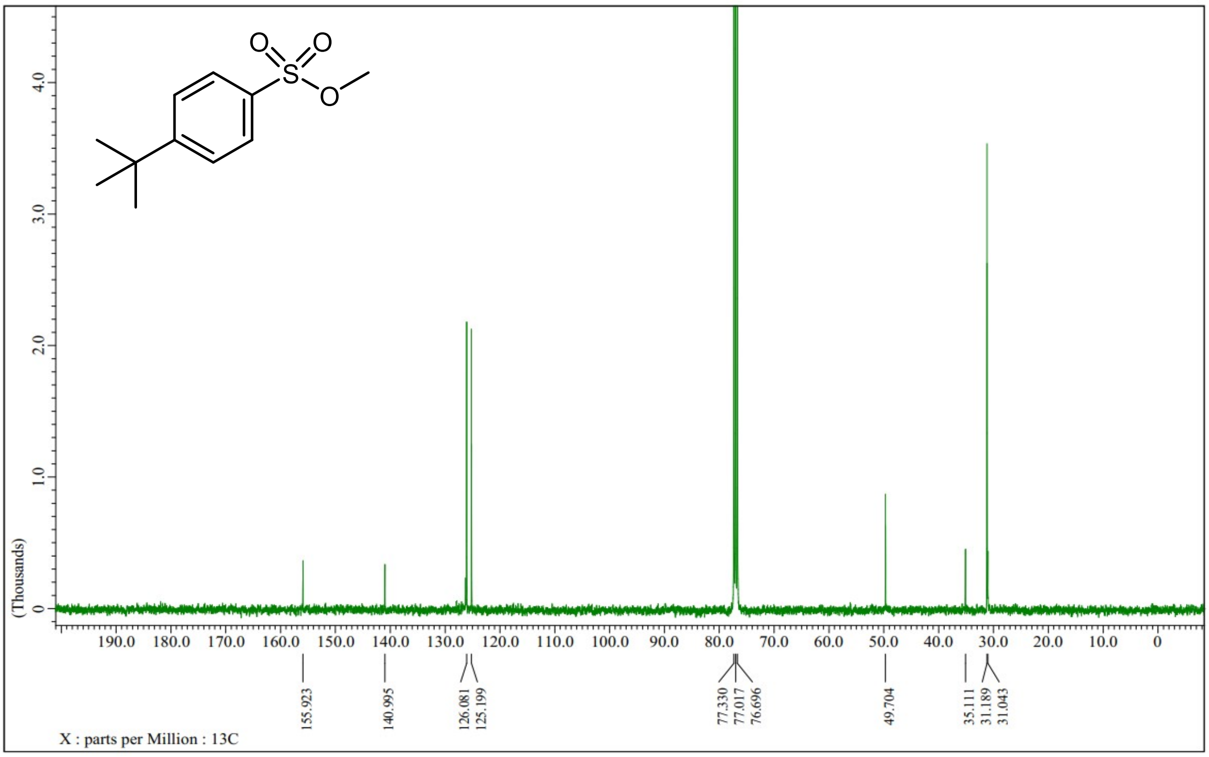

^{13}C NMR spectrum of methyl 4-(tert-butyl)benzenesulfonate (3f)

Methyl 4-methoxybenzenesulfonate (3g)

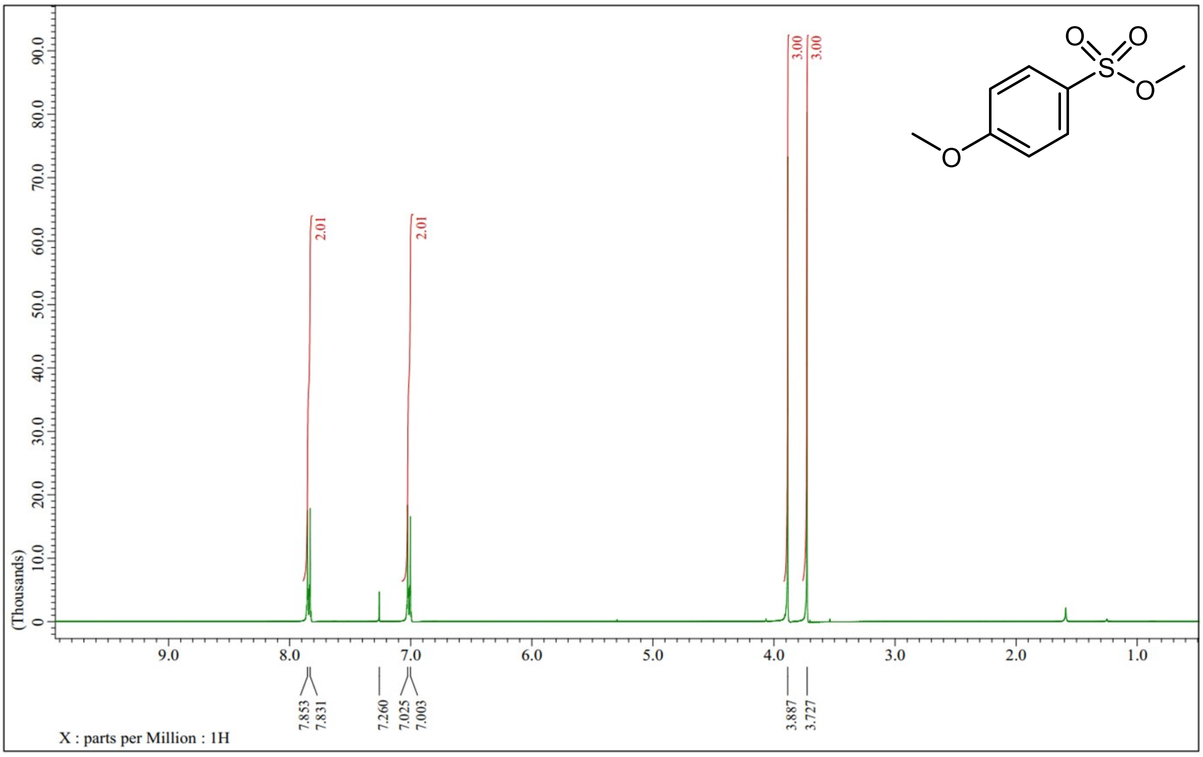

^{1}H NMR spectrum of methyl 4-methoxybenzenesulfonate (3g)

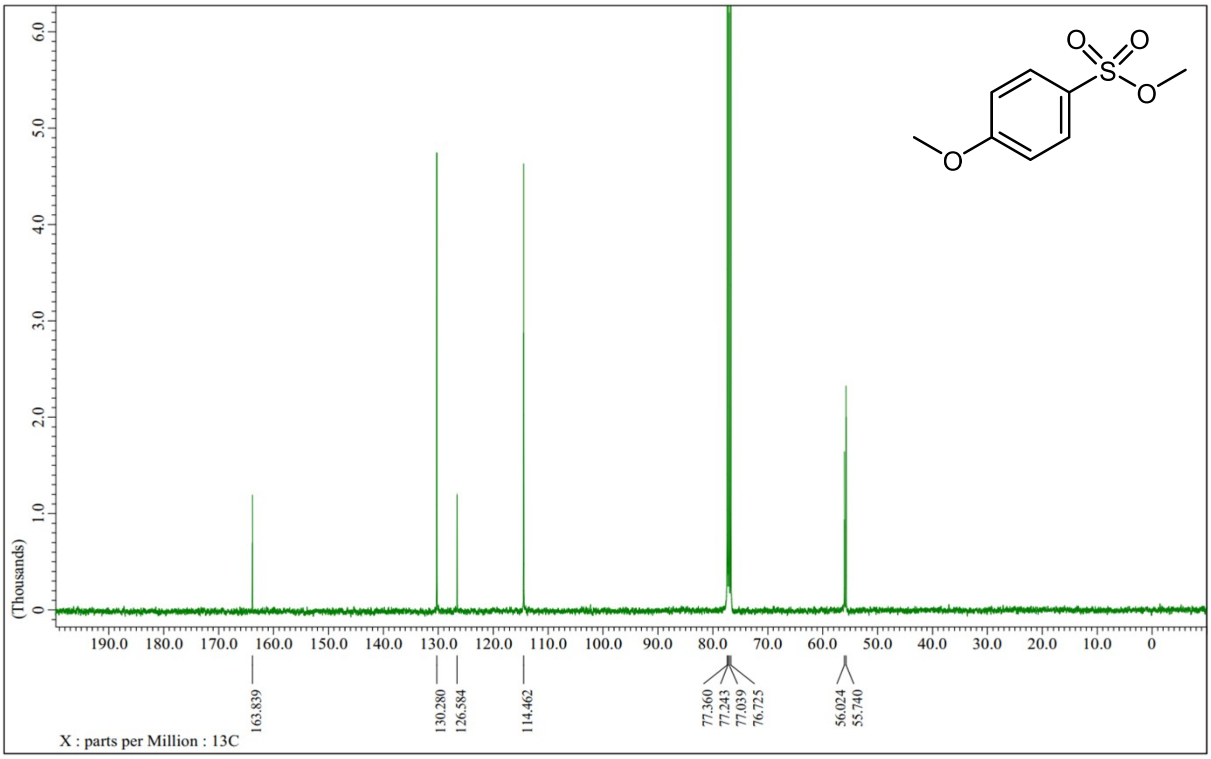

^{13}C NMR spectrum of of methyl 4-methoxybenzenesulfonate (3g)

Methyl 4-(benzyloxy)benzenesulfonate (3h)

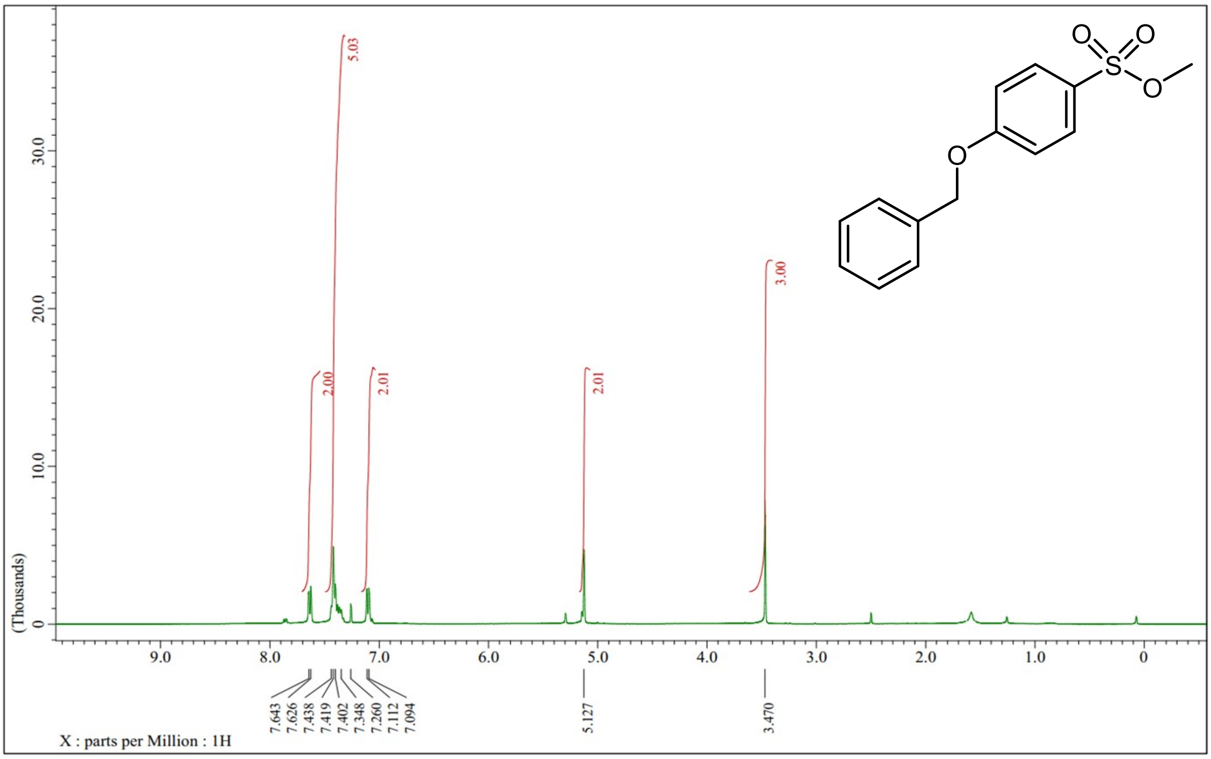

^{1}H NMR spectrum of methyl 4-(benzyloxy)benzenesulfonate (3h)

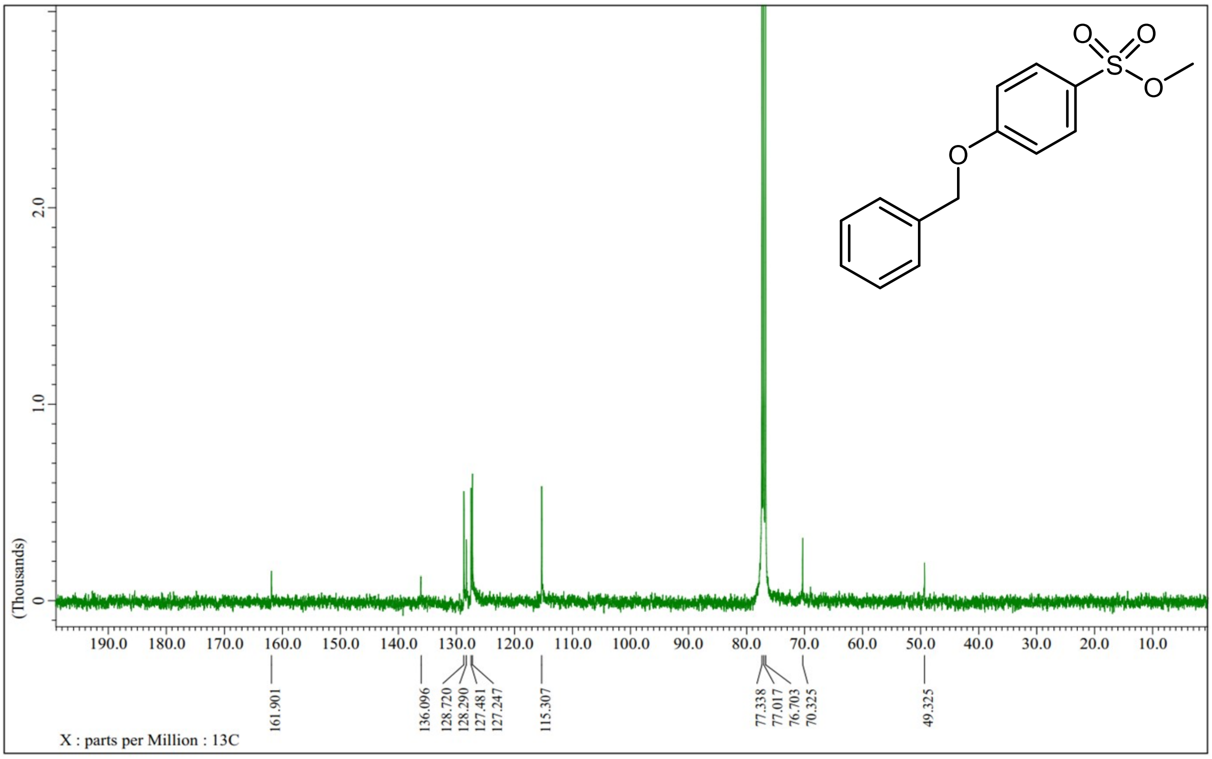

^{13}C NMR spectrum of methyl 4-(benzyloxy)benzenesulfonate (3h)

Methyl 3-chlorobenzenesulfonate (3i)

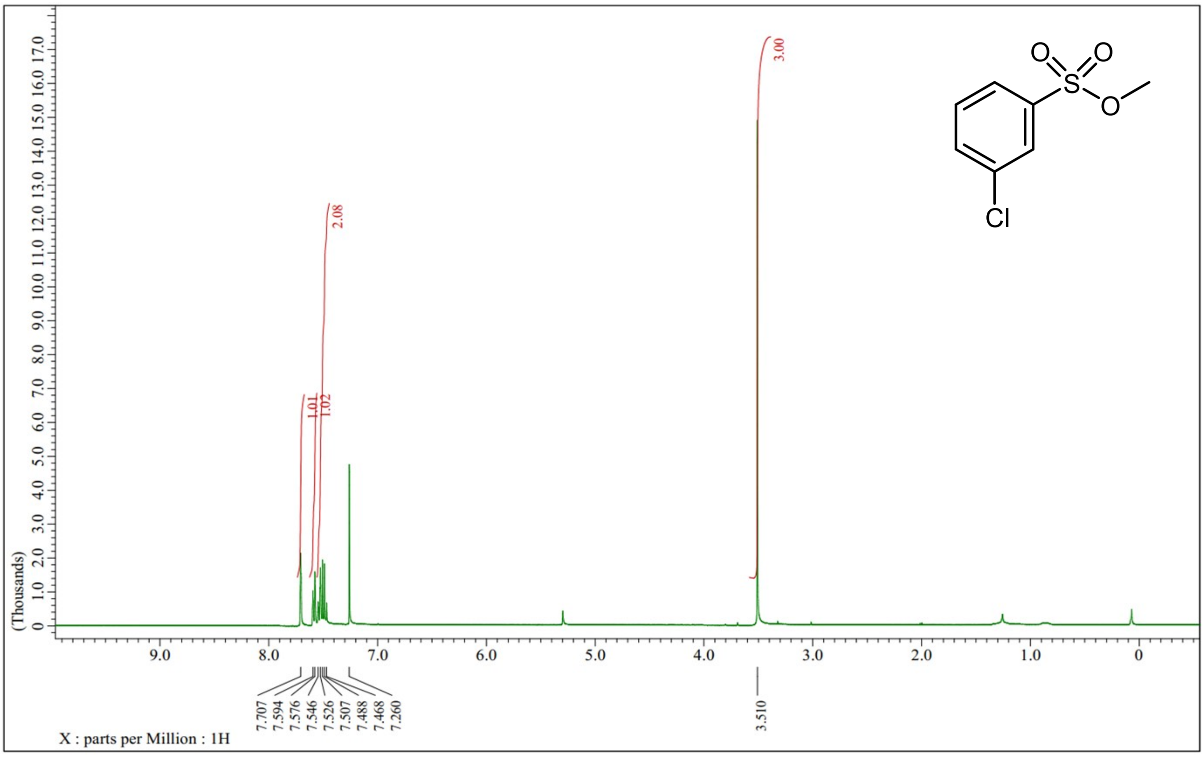

^{1}H NMR spectrum of methyl 3-chlorobenzenesulfonate (3i)

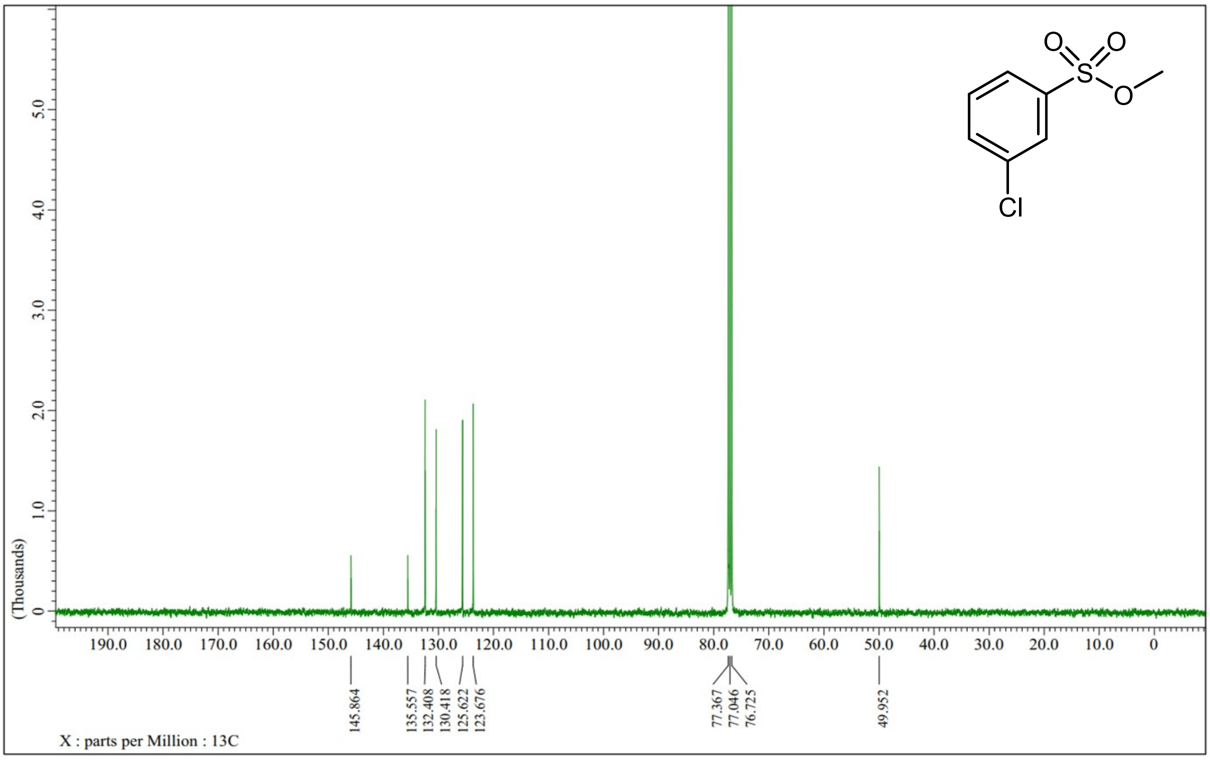

^{13}C NMR spectrum of methyl 3-chlorobenzenesulfonate (3i)

Methyl 3-methylbenzenesulfonate (3j)

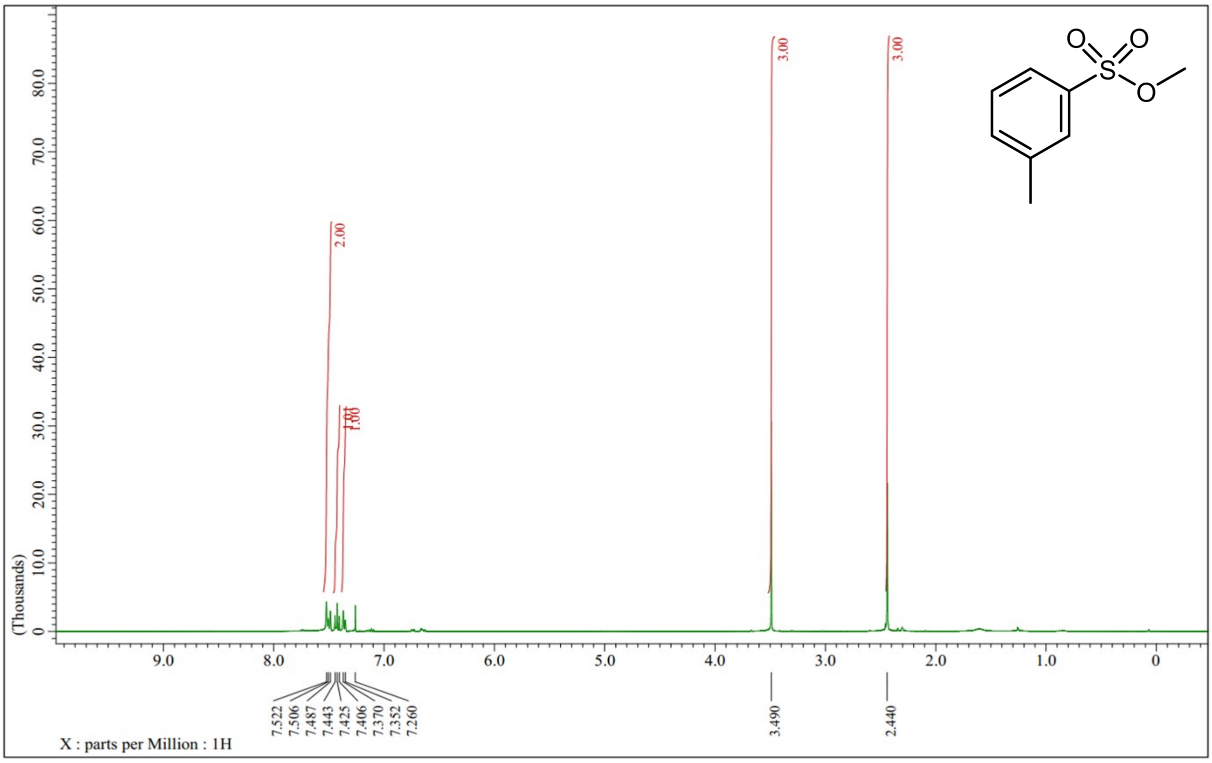

^{1}H NMR spectrum of methyl 3-methylbenzenesulfonate (3j)

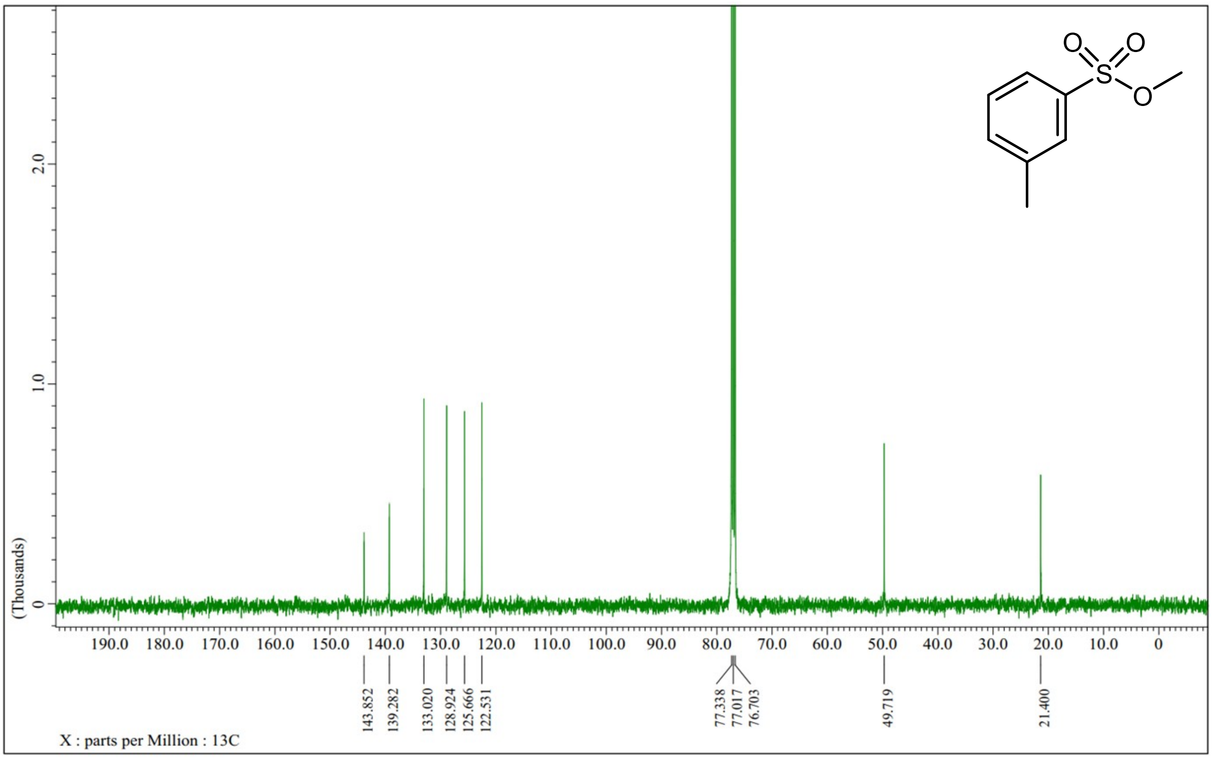

^{13}C NMR spectrum of methyl 3-methylbenzenesulfonate (3j)

Methyl 3,5-dichlorobenzenesulfonate (3k)

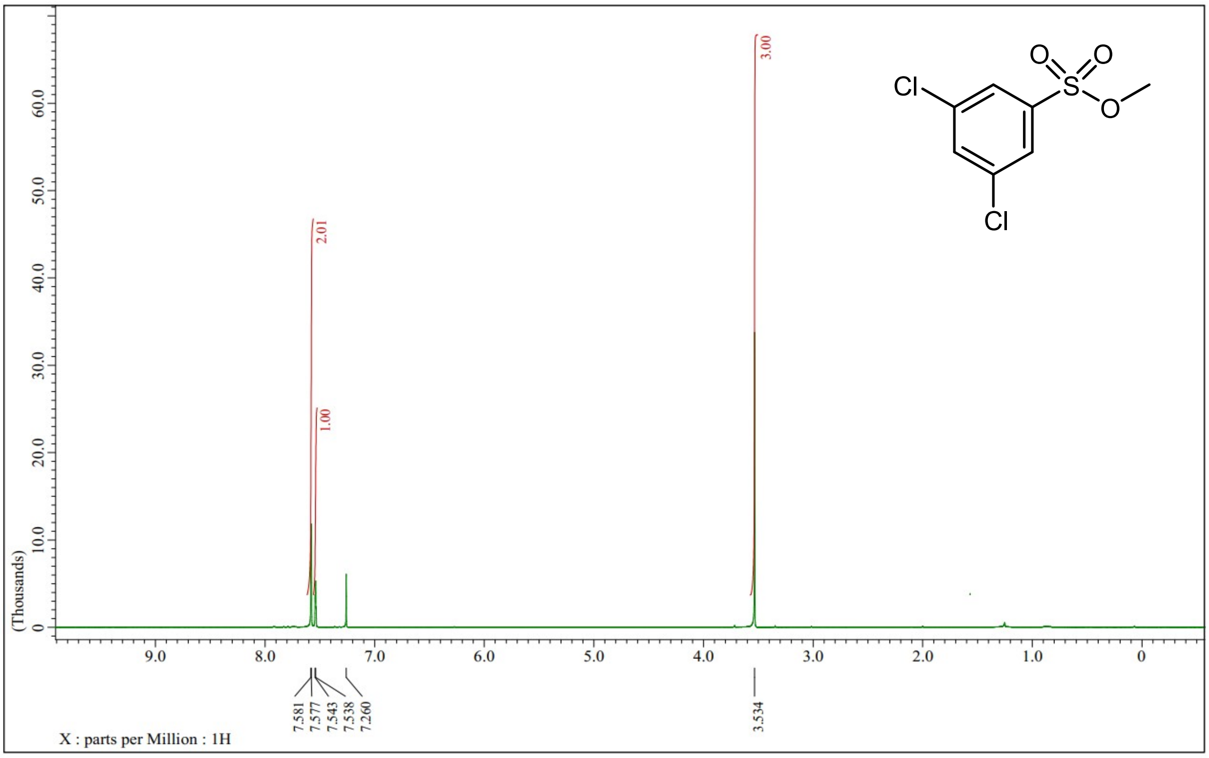

^{1}H NMR spectrum of methyl 3,5-dichlorobenzenesulfonate (3k)

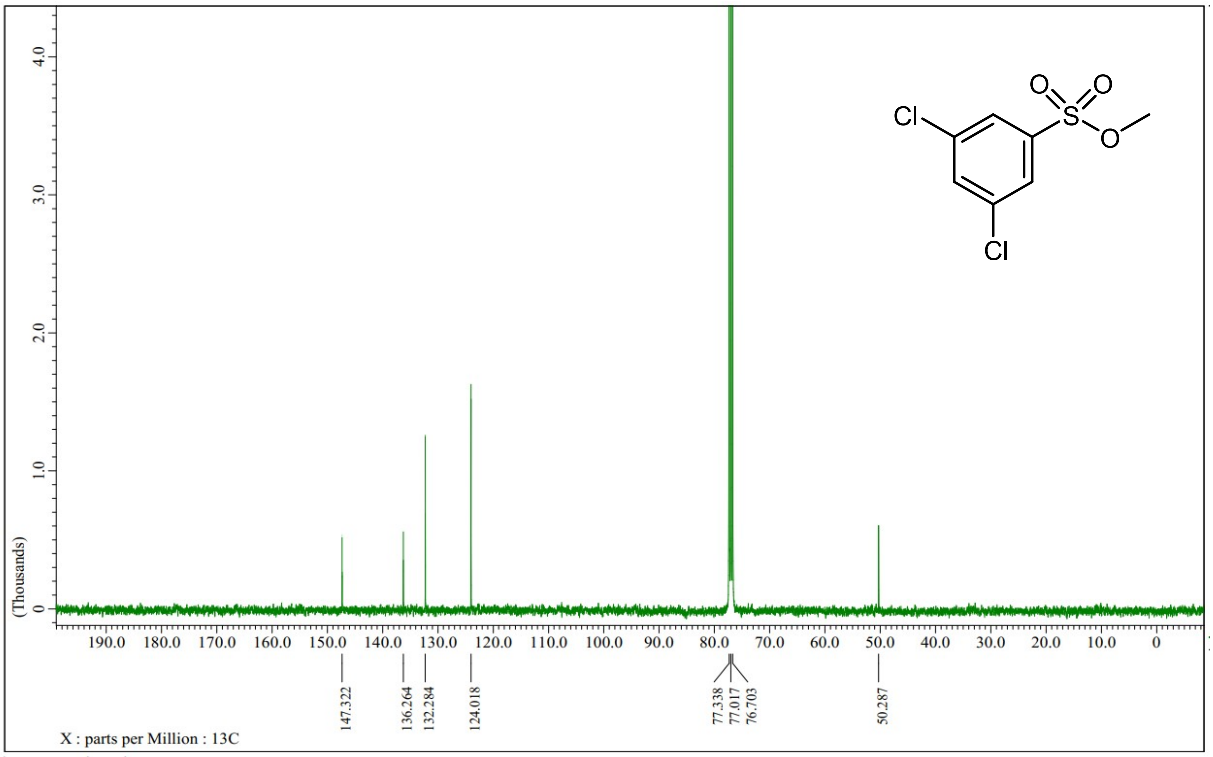

^{13}C NMR spectrum of methyl 3,5-dichlorobenzenesulfonate (3k)

Methyl 3,5-dimethylbenzenesulfonate (3l)

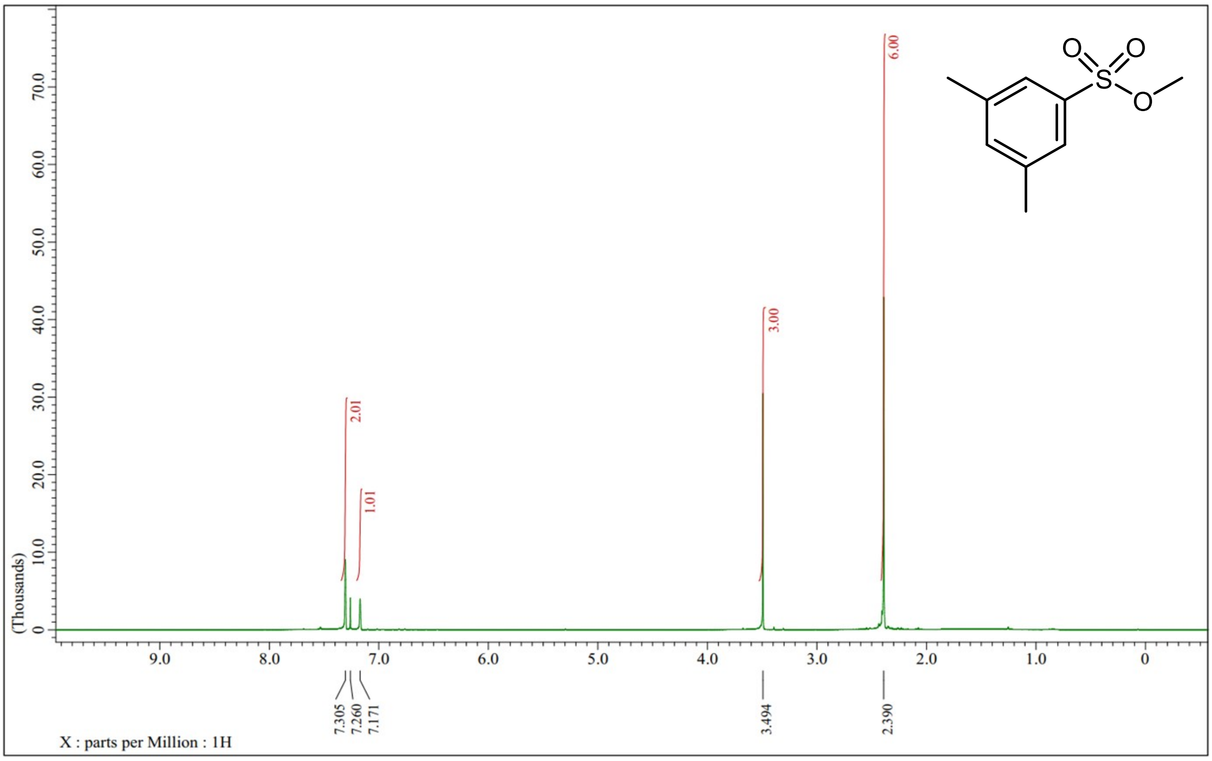

^{1}H NMR spectrum of methyl 3,5-dimethylbenzenesulfonate (3l)

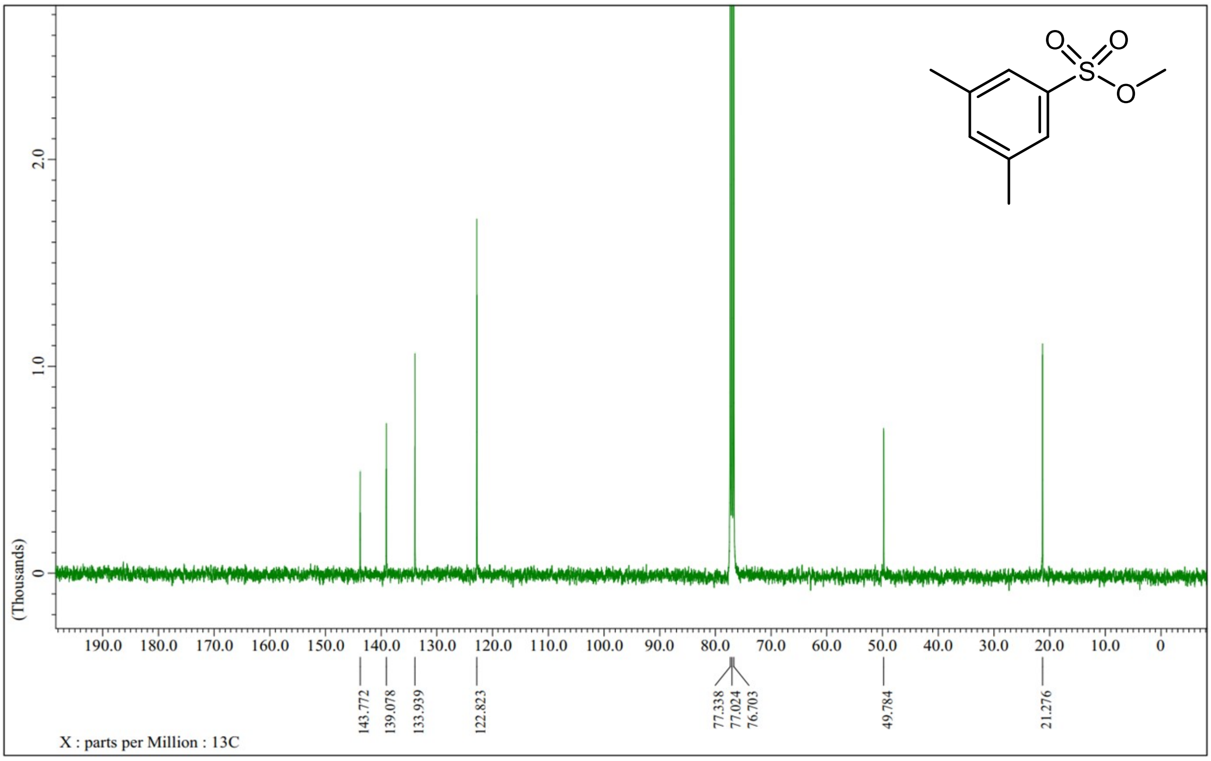

^{13}C NMR spectrum of methyl 3,5-dimethylbenzenesulfonate (3l)

Methyl 4-naphthalene-1-sulfonate (3m)

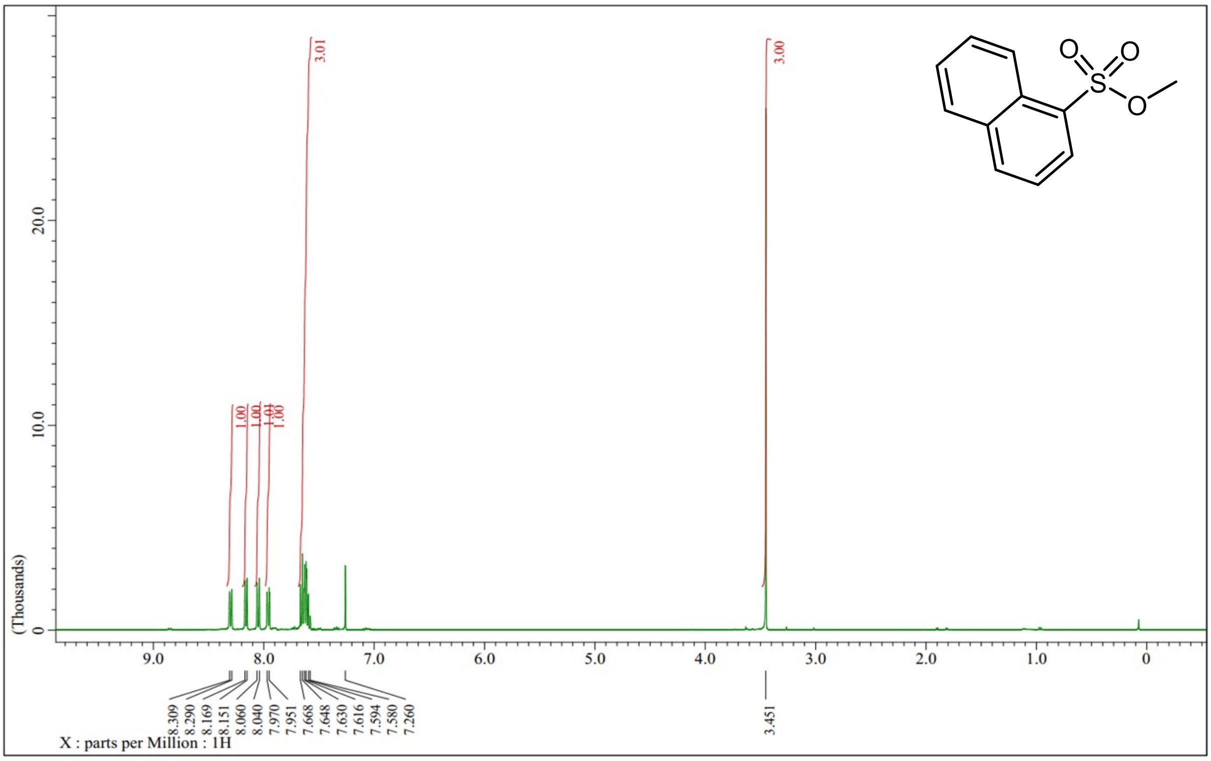

^{1}H NMR spectrum of methyl 4-naphthalene-1-sulfonate (3m)

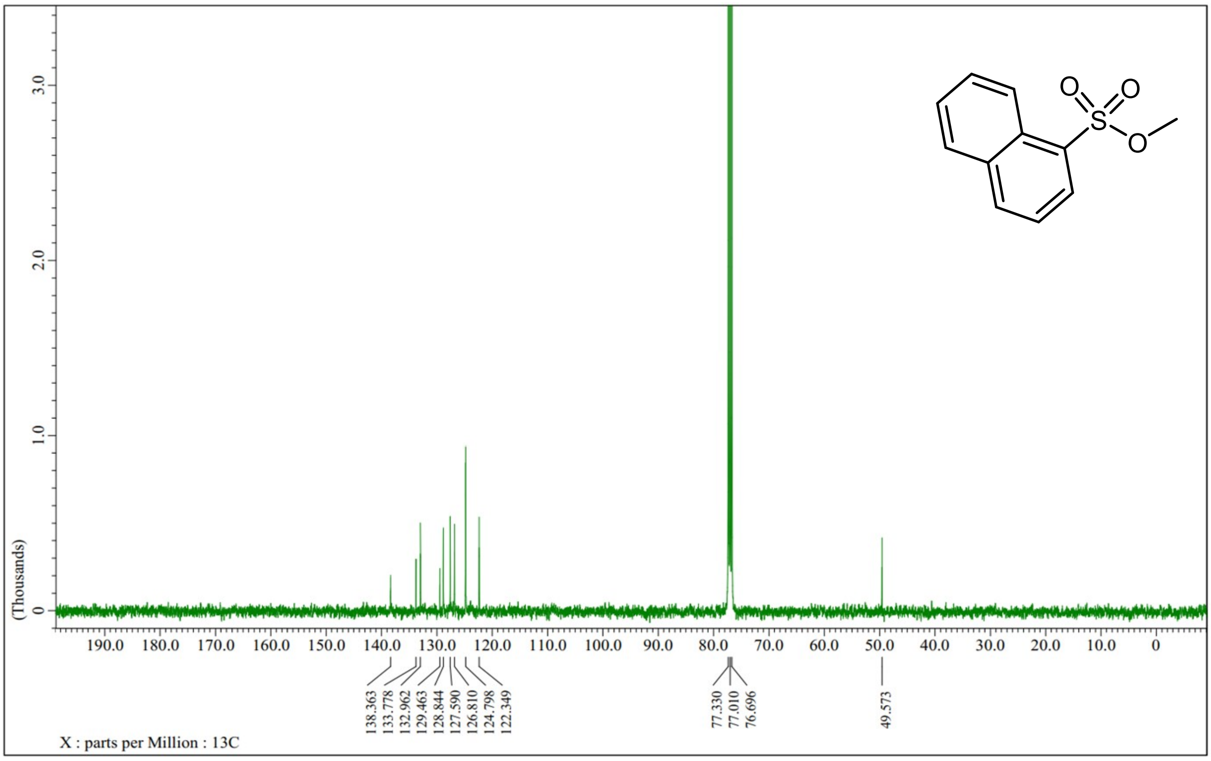

^{13}C NMR spectrum of methyl 4-naphthalene-1-sulfonate (3m)

Methyl benzo[*d*][1,3]dioxole-5-sulfonate (3n)

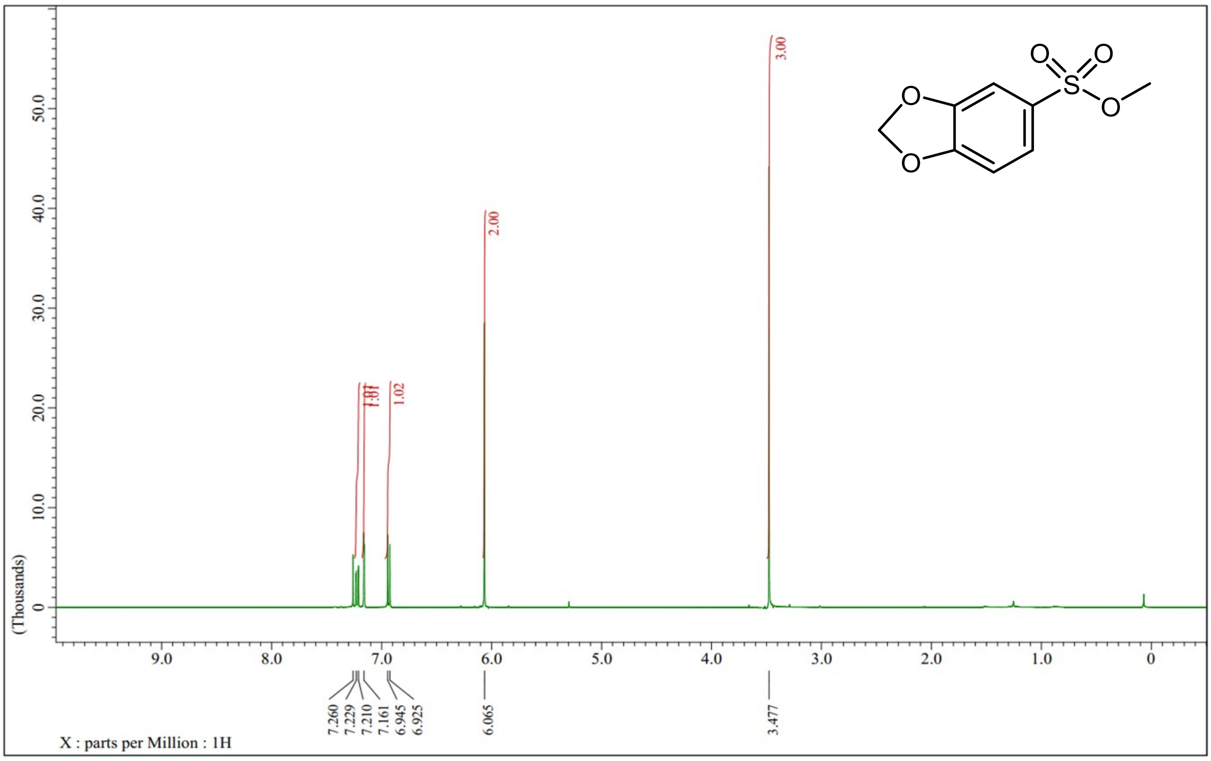

^{1}H NMR spectrum of methyl benzo[*d*][1,3]dioxole-5-sulfonate (3n)

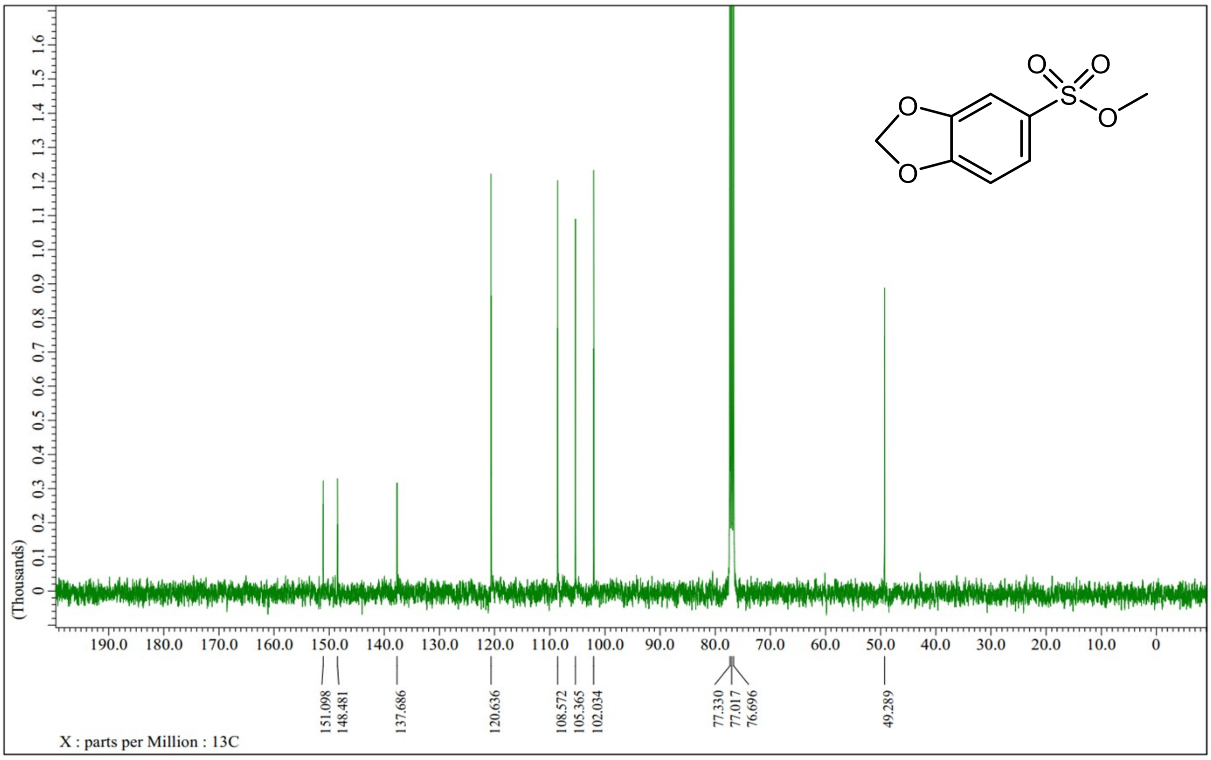

^{13}C NMR spectrum of methyl benzo[*d*][1,3]dioxole-5-sulfonate (3n)

Methyl 4-(N-(4,6-dimethylpyrimidin-2-yl)sulfamoyl)benzenesulfonate (3o)

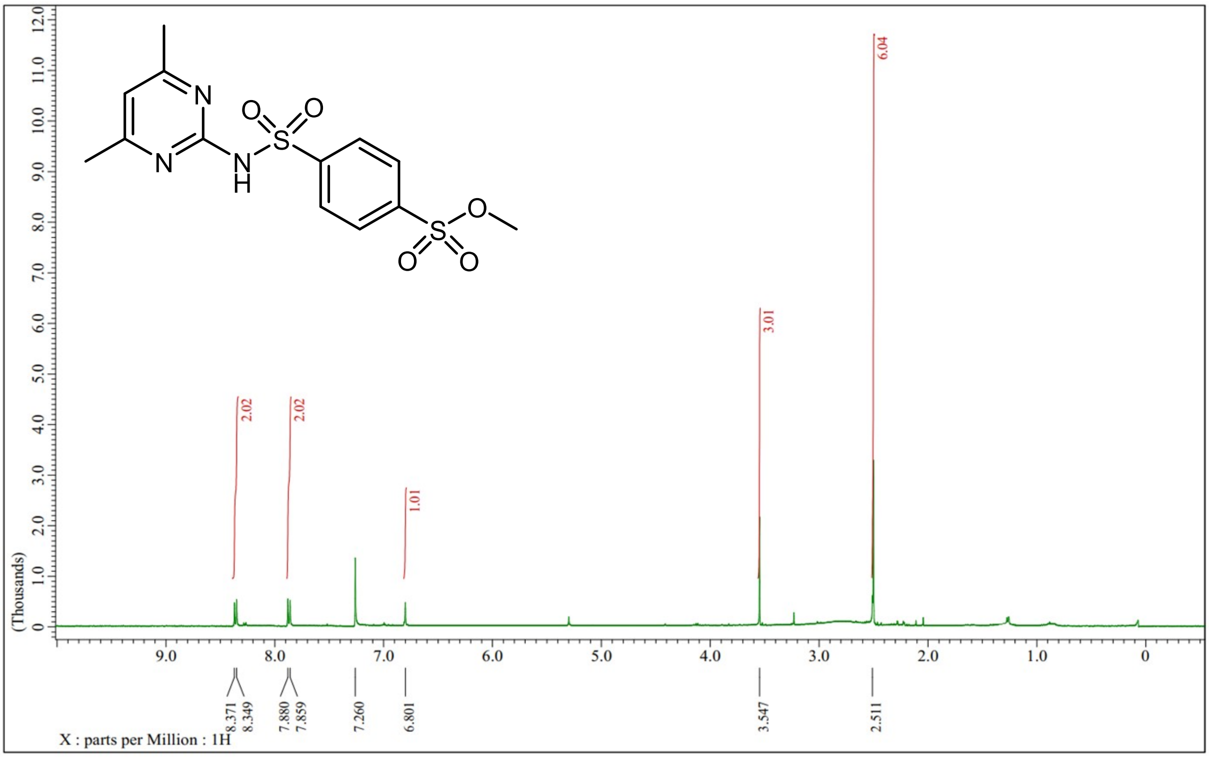

^{1}H NMR spectrum of methyl 4-(N-(4,6-dimethylpyrimidin-2-yl)sulfamoyl)benzenesulfonate (3o)

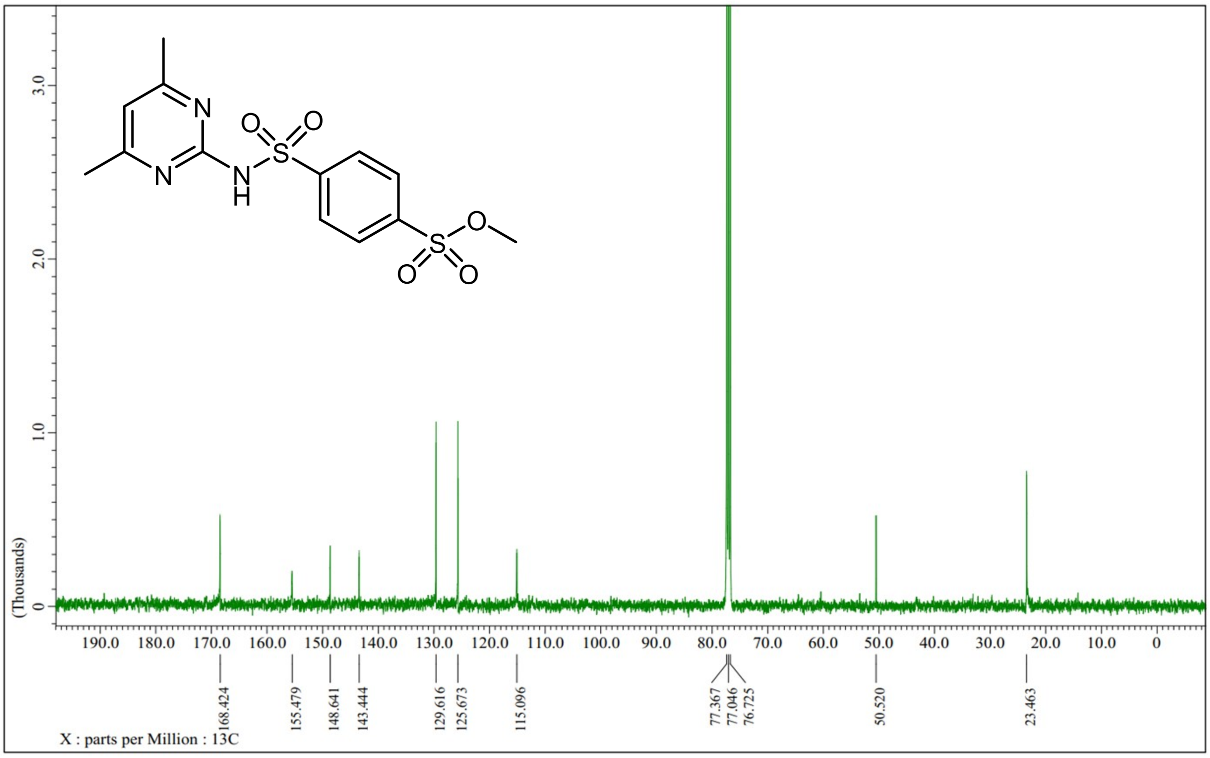

^{13}C NMR spectrum of methyl 4-(N-(4,6-dimethylpyrimidin-2-yl)sulfamoyl)benzenesulfonate (3o)

Ethyl benzenesulfonate (4a)

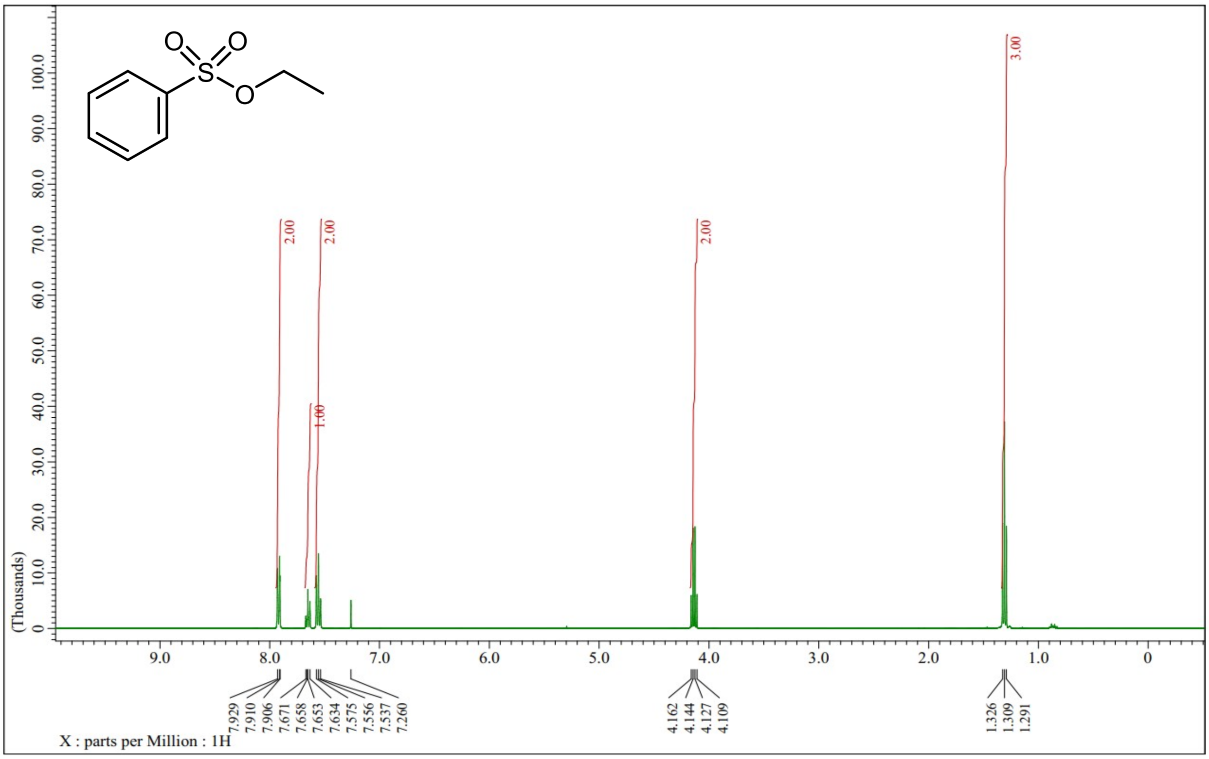

^{1}H NMR spectrum of ethyl benzenesulfonate (4a)

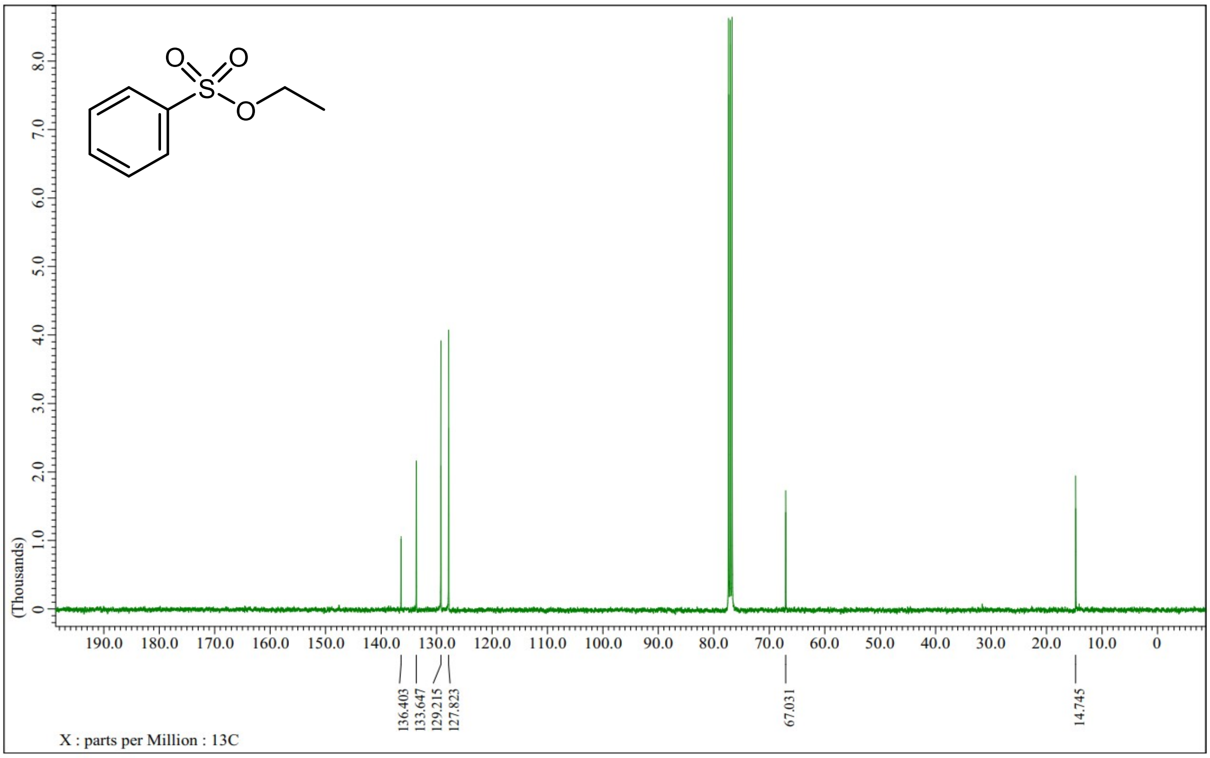

^{13}C NMR spectrum of ethyl benzenesulfonate (4a)

Butyl benzenesulfonate (4b)

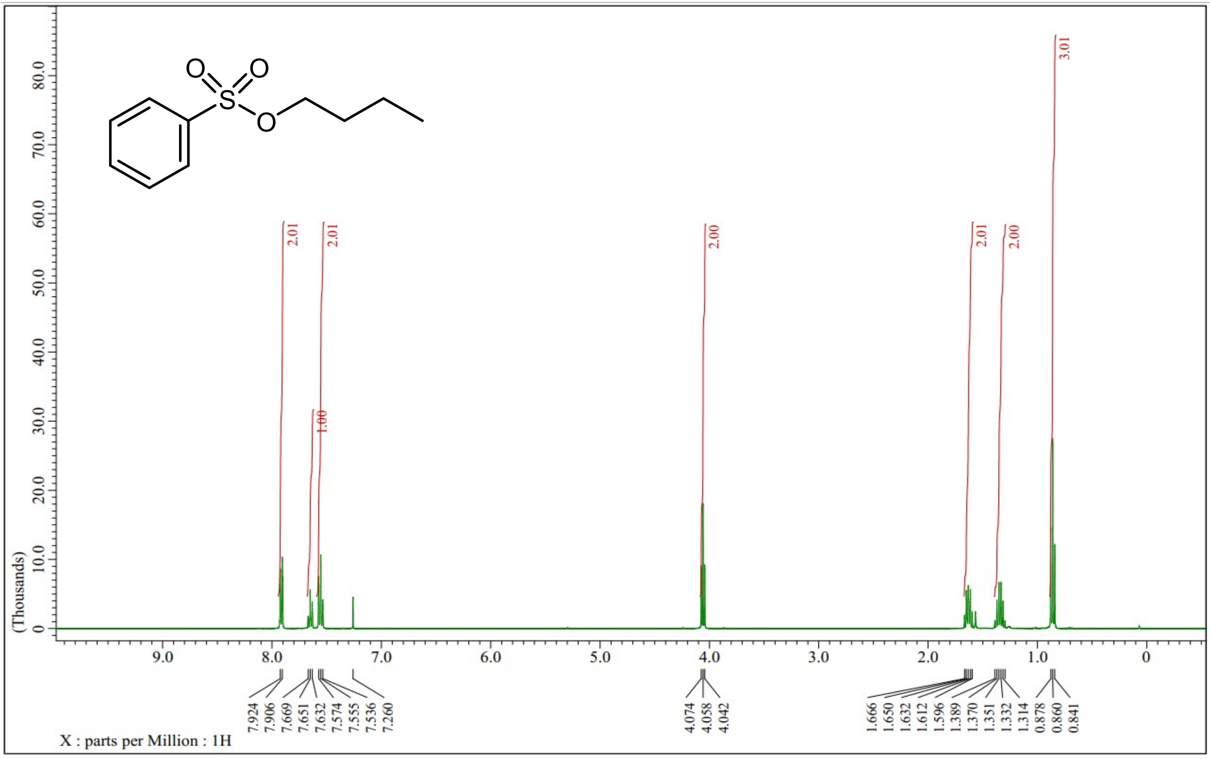

^{1}H NMR spectrum of butyl benzenesulfonate (4b)

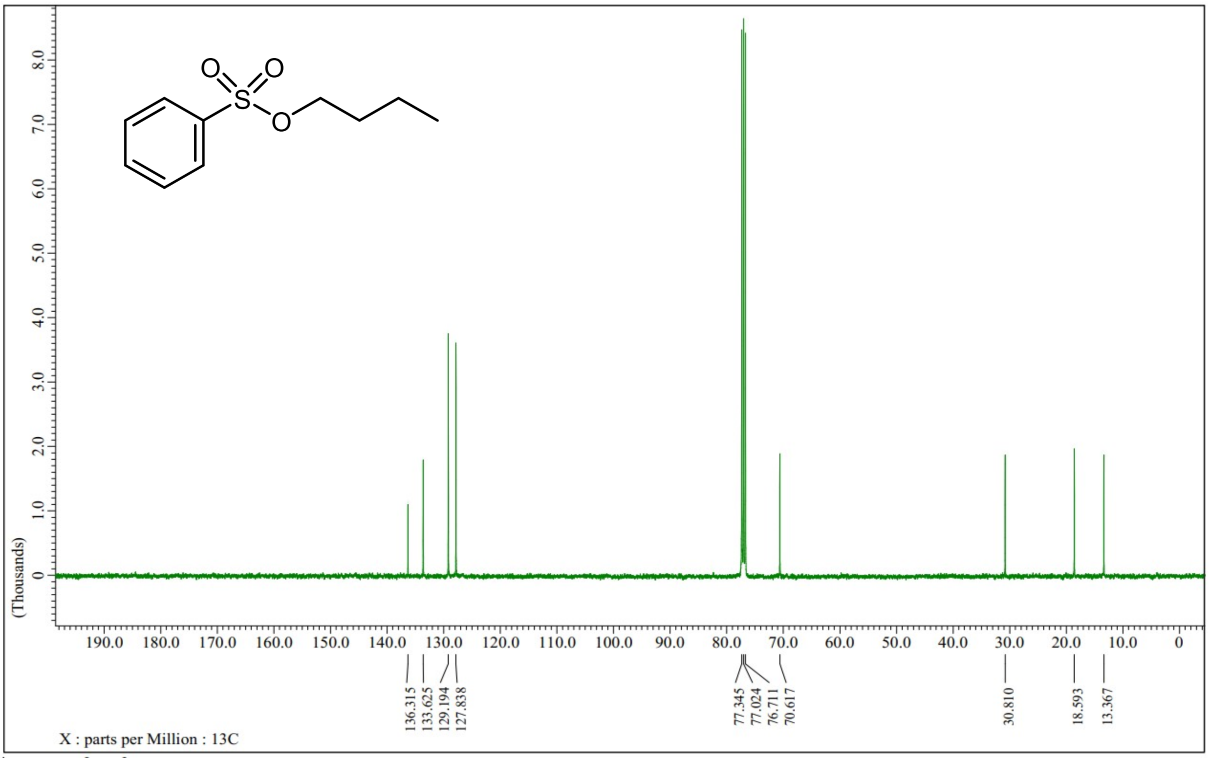

^{13}C NMR spectrum of butyl benzenesulfonate (4b)

Heptyl benzenesulfonate (4c)

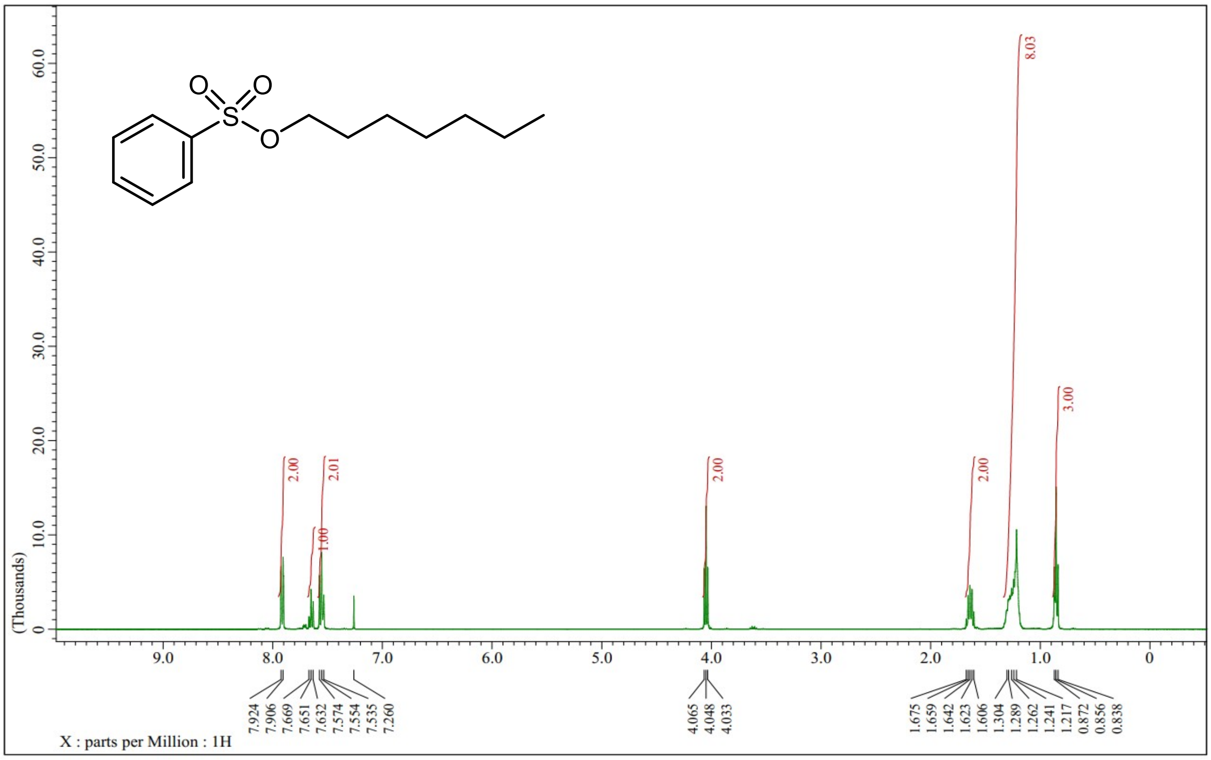

^{1}H NMR spectrum of heptyl benzenesulfonate (4c)

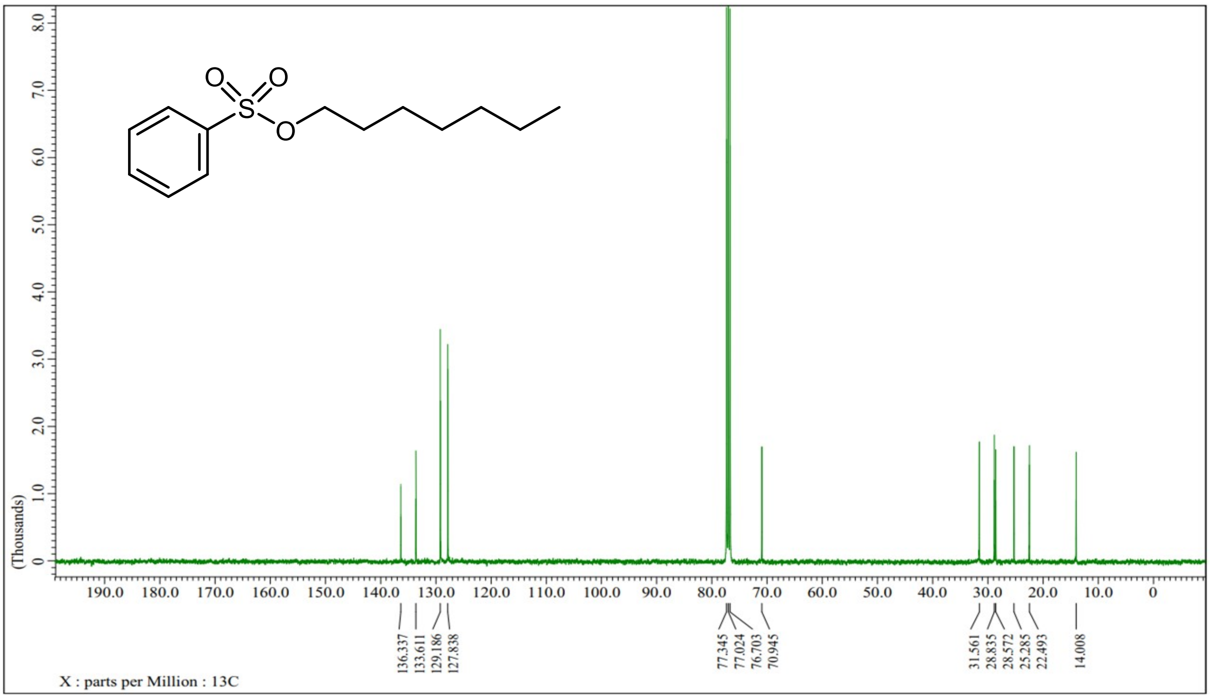

^{13}C NMR spectrum of heptyl benzenesulfonate (4c)

Isobutyl benzenesulfonate (4d)

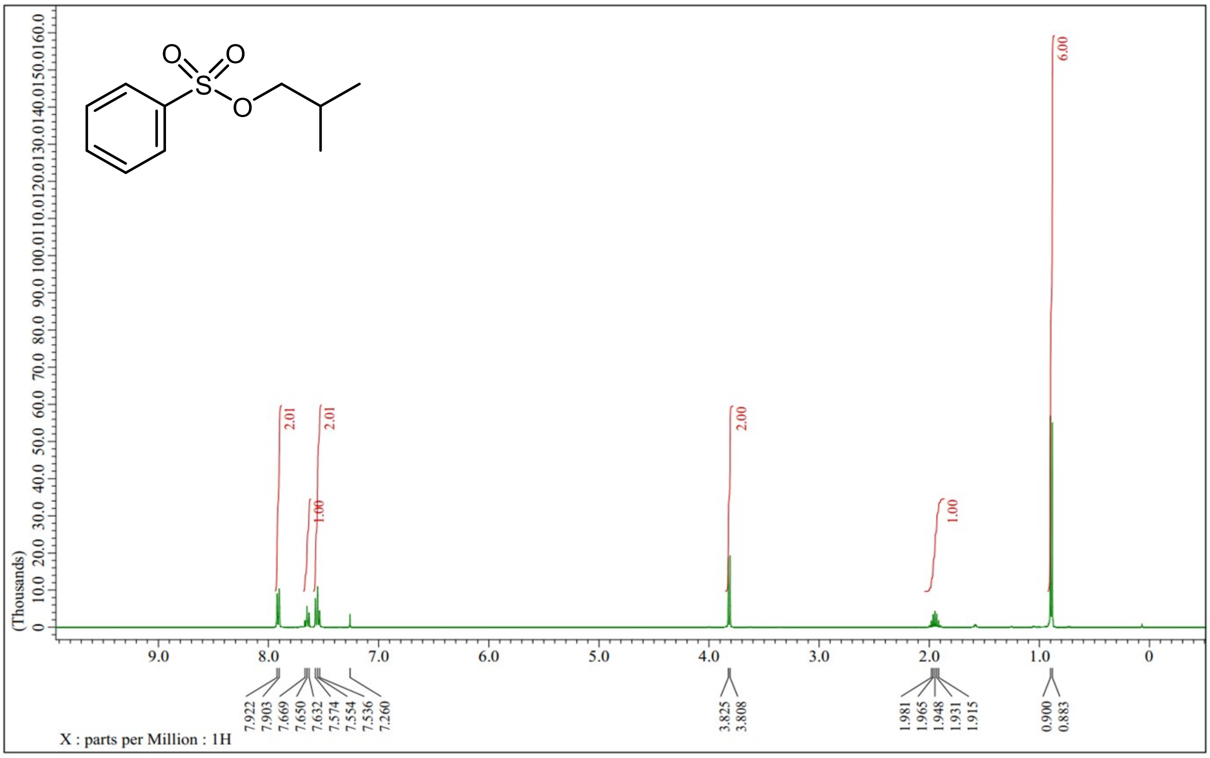

^{1}H NMR spectrum of methyl isobutyl benzenesulfonate (4d)

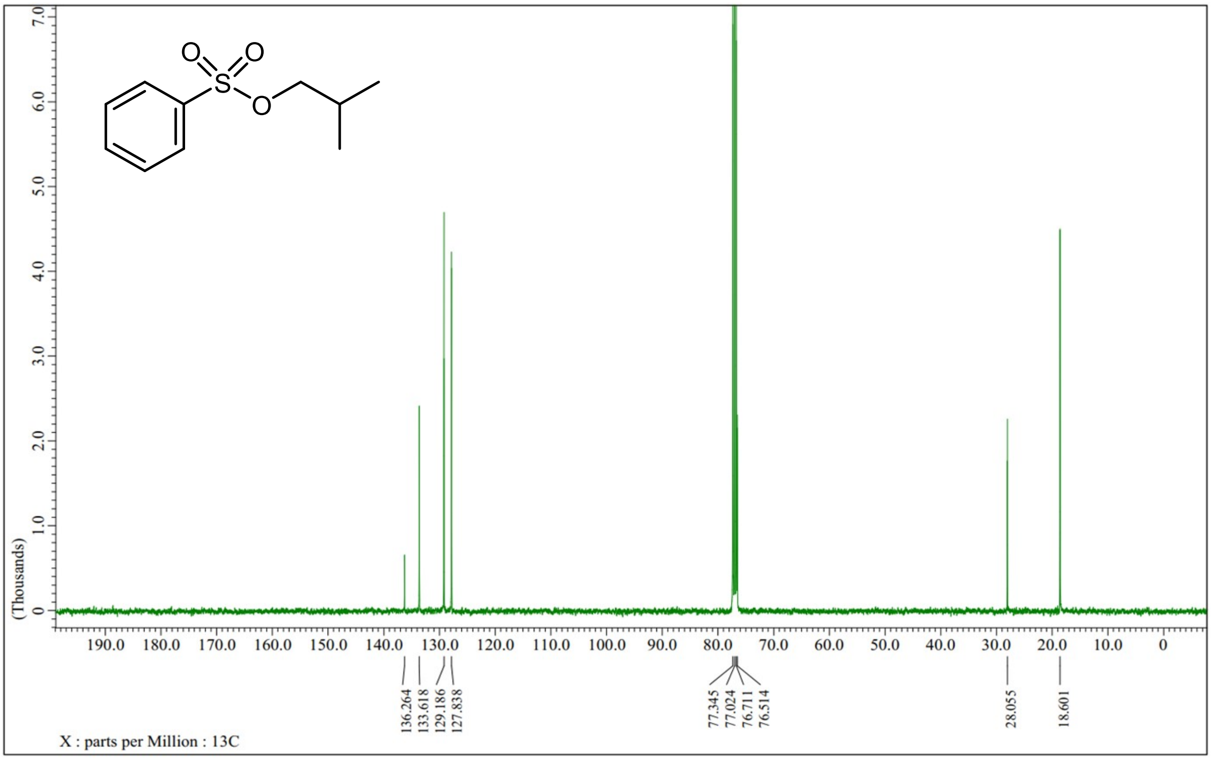

^{13}C NMR spectrum of isobutyl benzenesulfonate (4d)

Benzyl benzenesulfonate (4e)

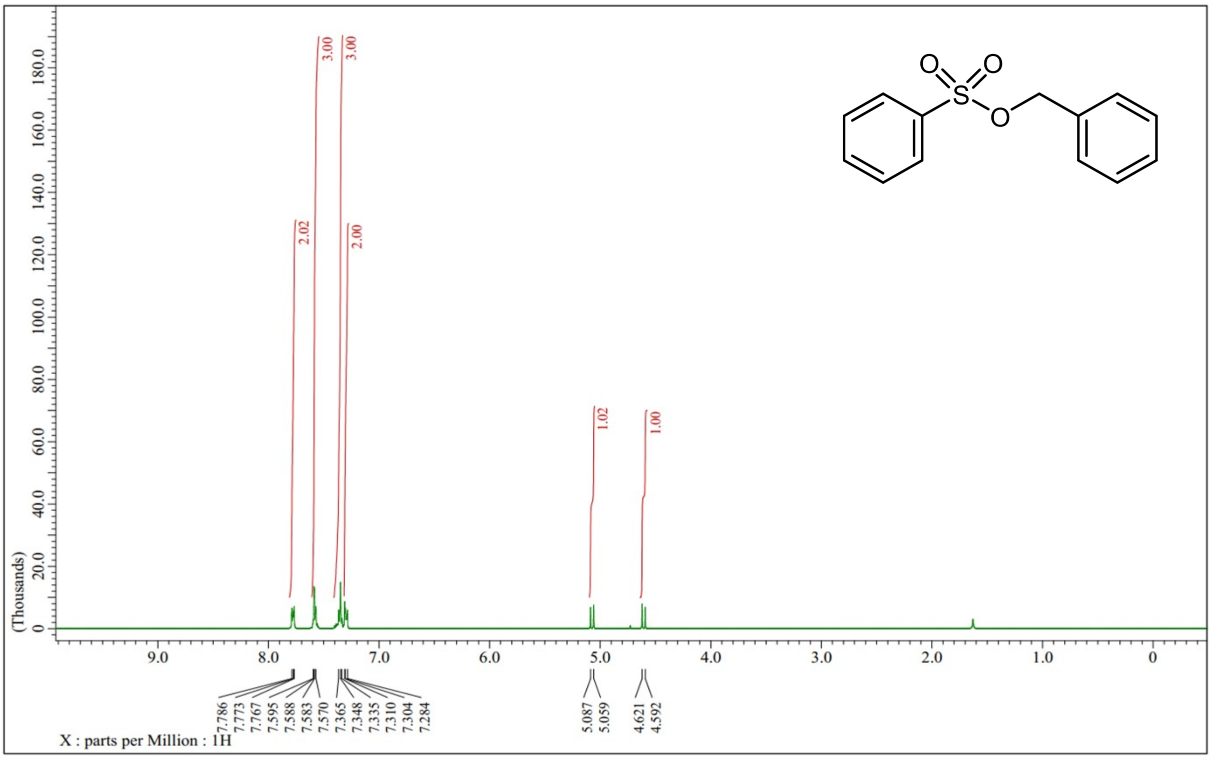

^{1}H NMR spectrum of benzyl benzenesulfonate (4e)

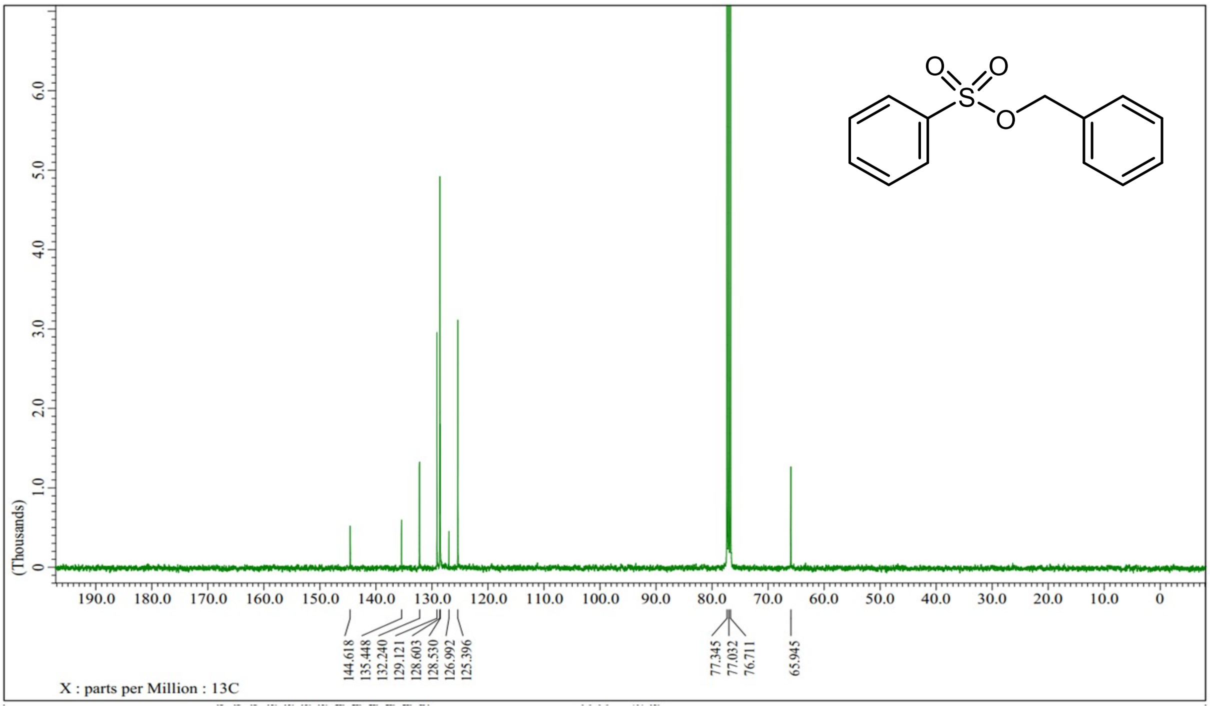

^{13}C NMR spectrum of benzyl benzenesulfonate (4e)

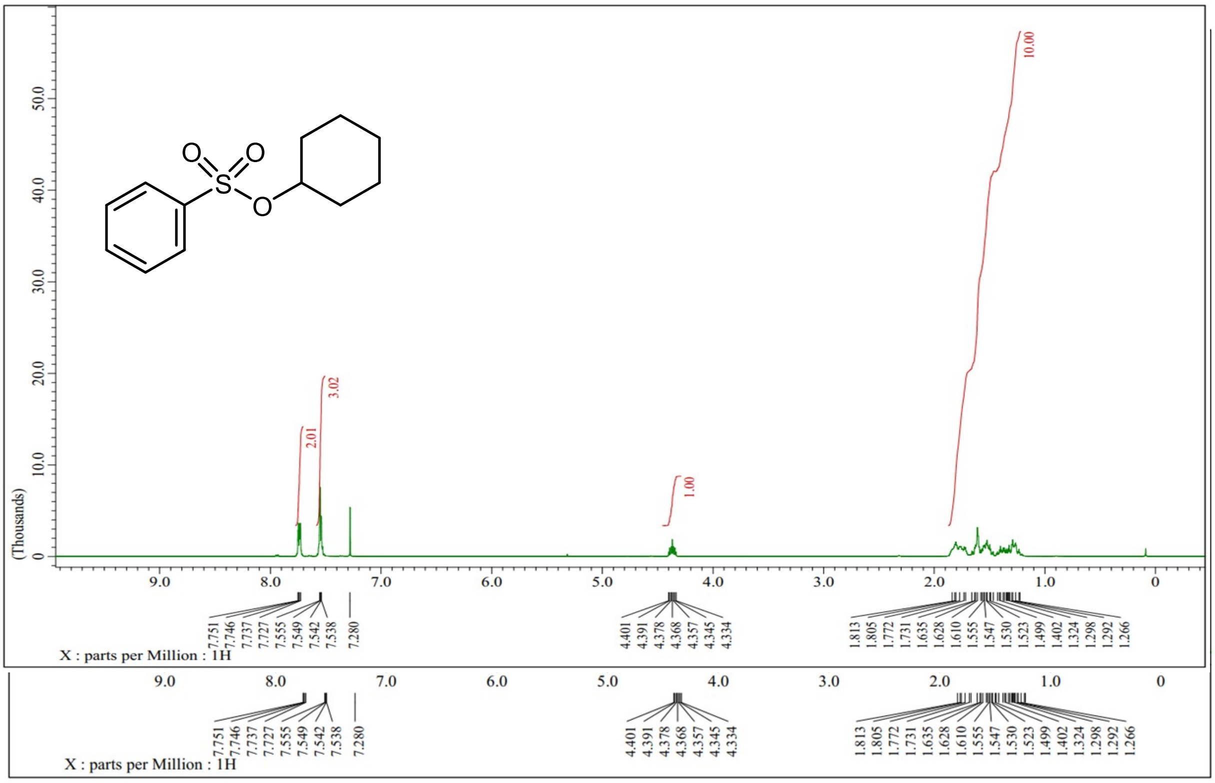

Cyclohexyl benzenesulfonate (4f)

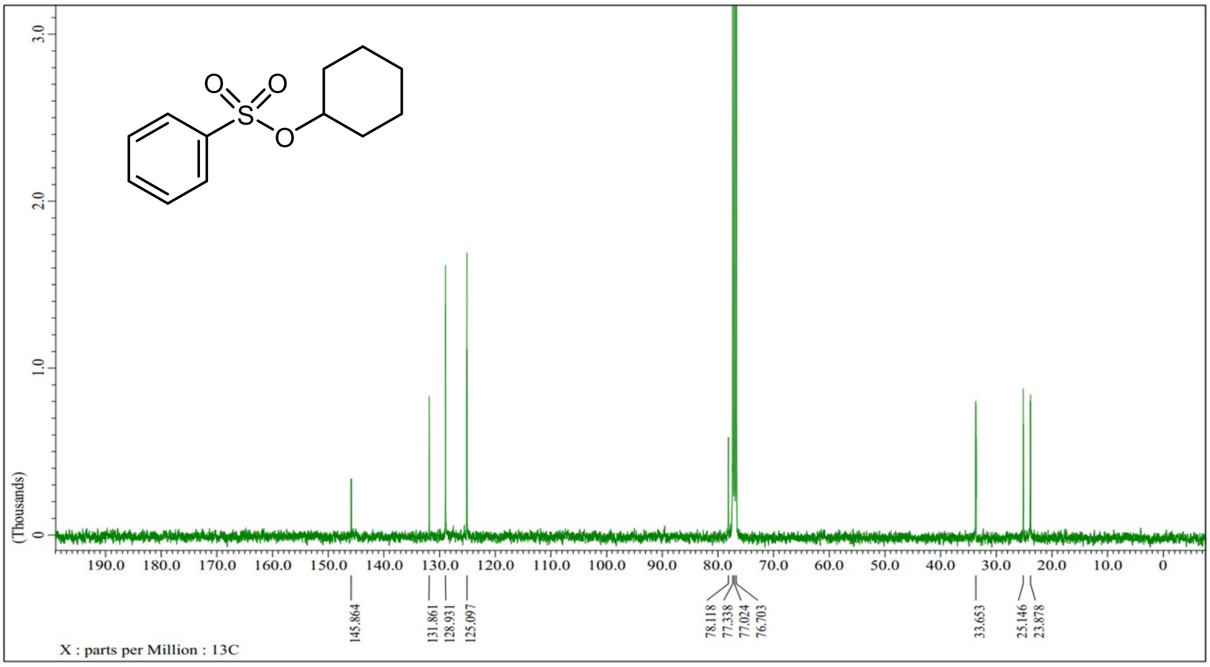

^{1}H NMR spectrum of methyl cyclohexyl benzenesulfonate (4f)

^{13}C NMR spectrum of cyclohexyl benzenesulfonate (4f)

Tert-butyl benzenesulfonate (4g)

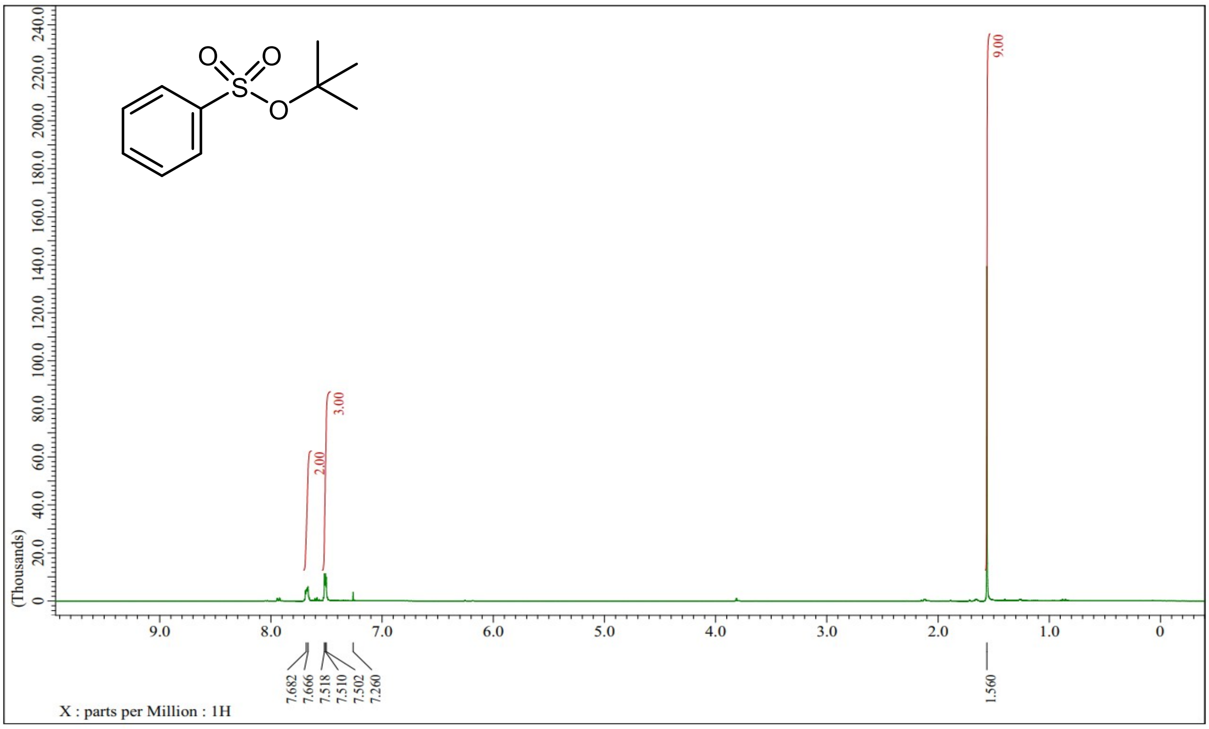

^{1}H NMR spectrum of tert-butyl benzenesulfonate (4g)

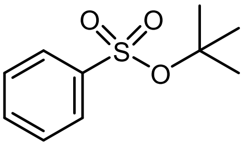

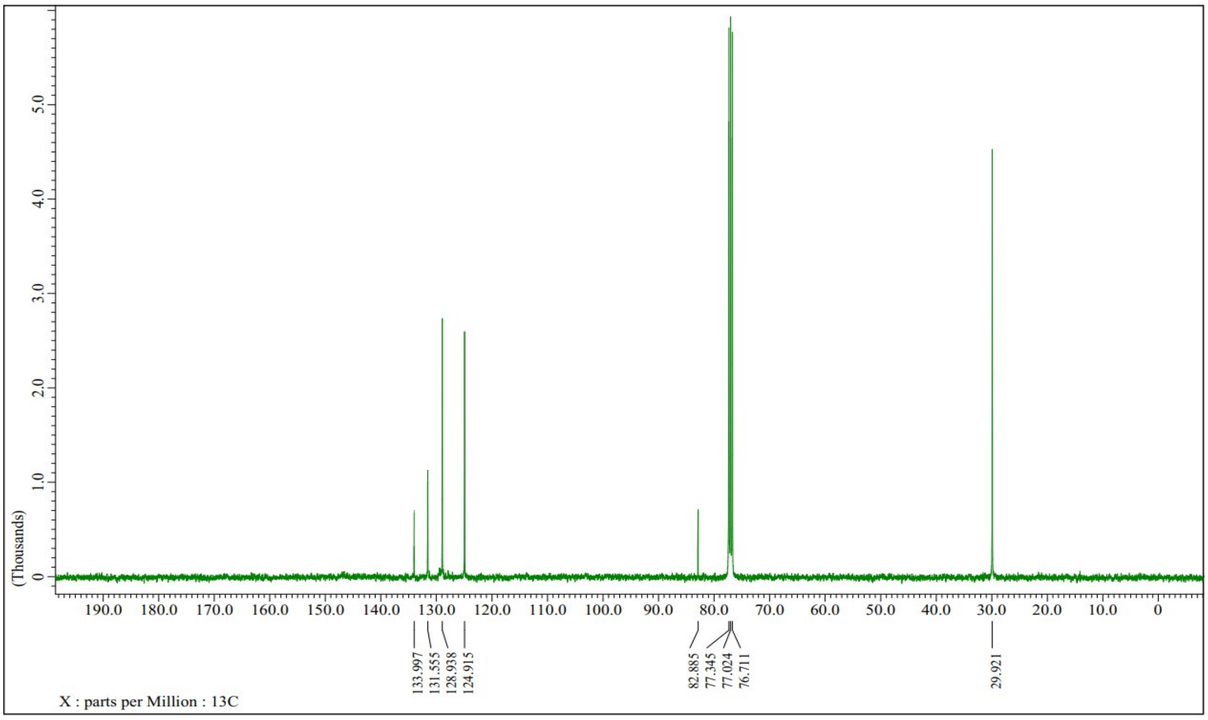

^{13}C NMR spectrum of tert-butyl benzenesulfonate (4g)

***Tert*-butyl 4-nitrobenzensulfonate (4h)**

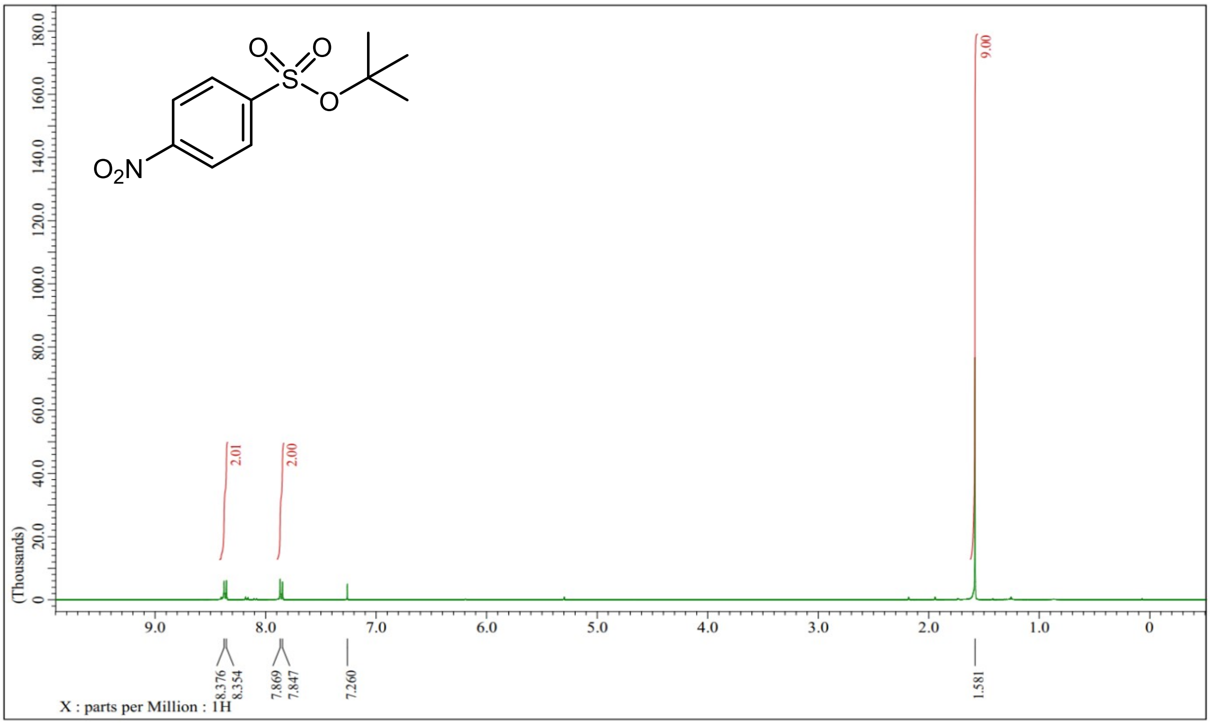

^{1}H NMR spectrum of *tert*-butyl 4-nitrobenzensulfonate (4h)

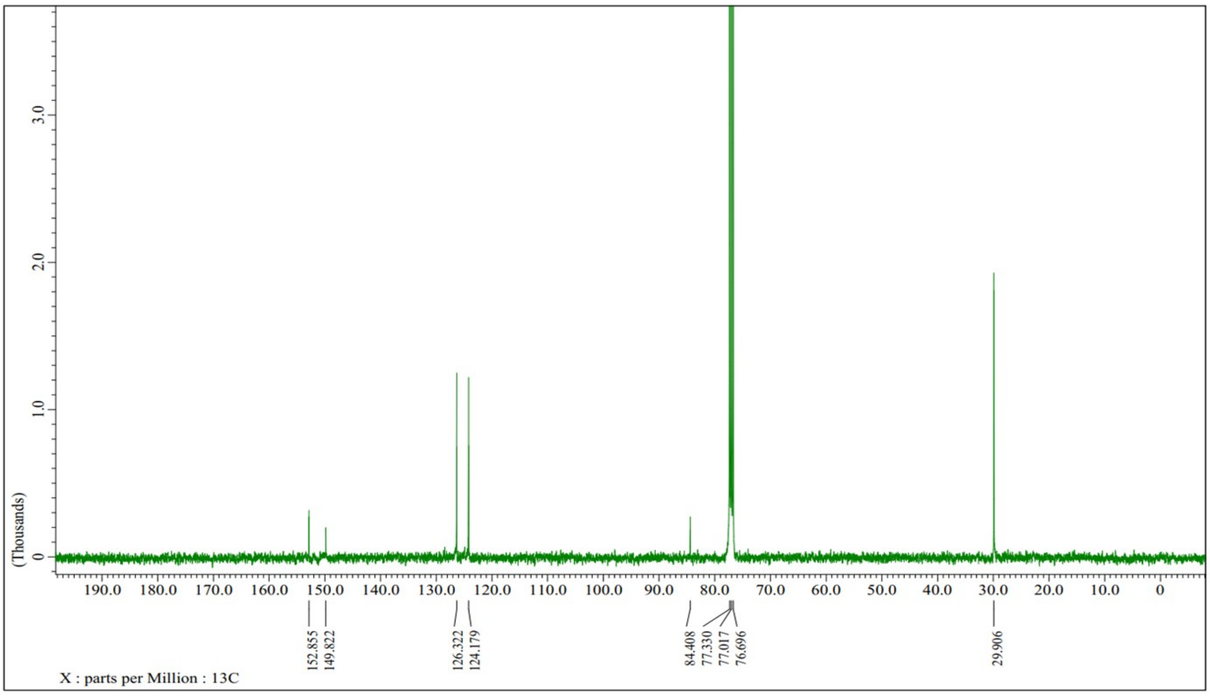

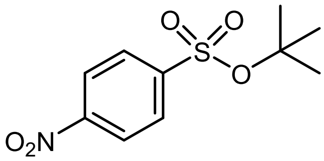

^{13}C NMR spectrum of ^{1}H NMR spectrum of *tert*-butyl 4-nitrobenzensulfonate (4h)

Tert-butyl 4-methylbenzenesulfonate (4i)

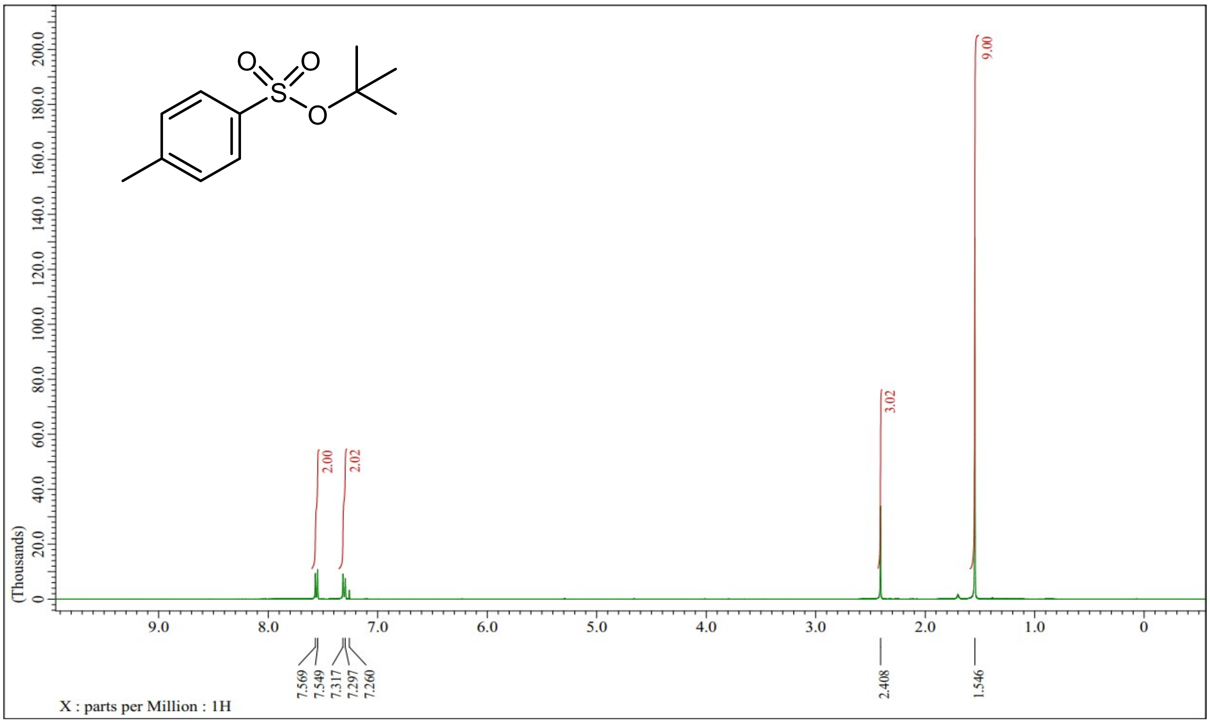

^{1}H NMR spectrum of tert-butyl 4-methylbenzenesulfonate (4i)

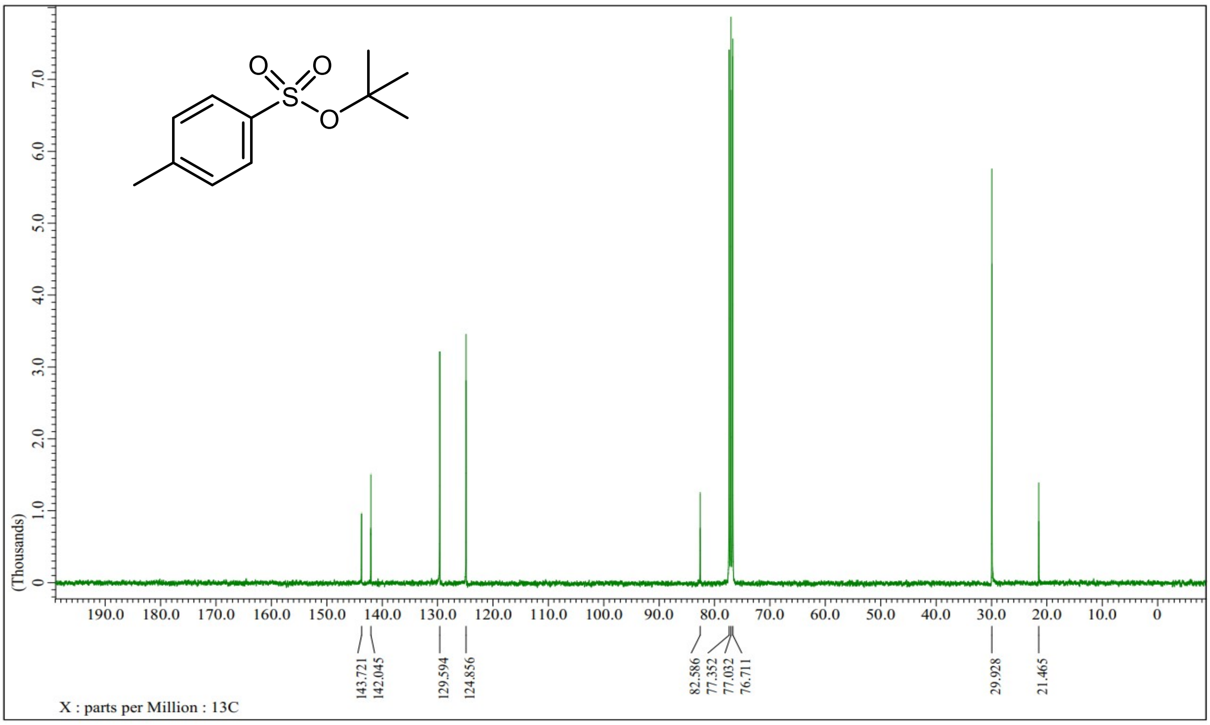

^{13}C NMR spectrum of tert-butyl 4-methylbenzenesulfonate (4i)

Tert-butyl 4-ethylbenzenesulfonate (4j)

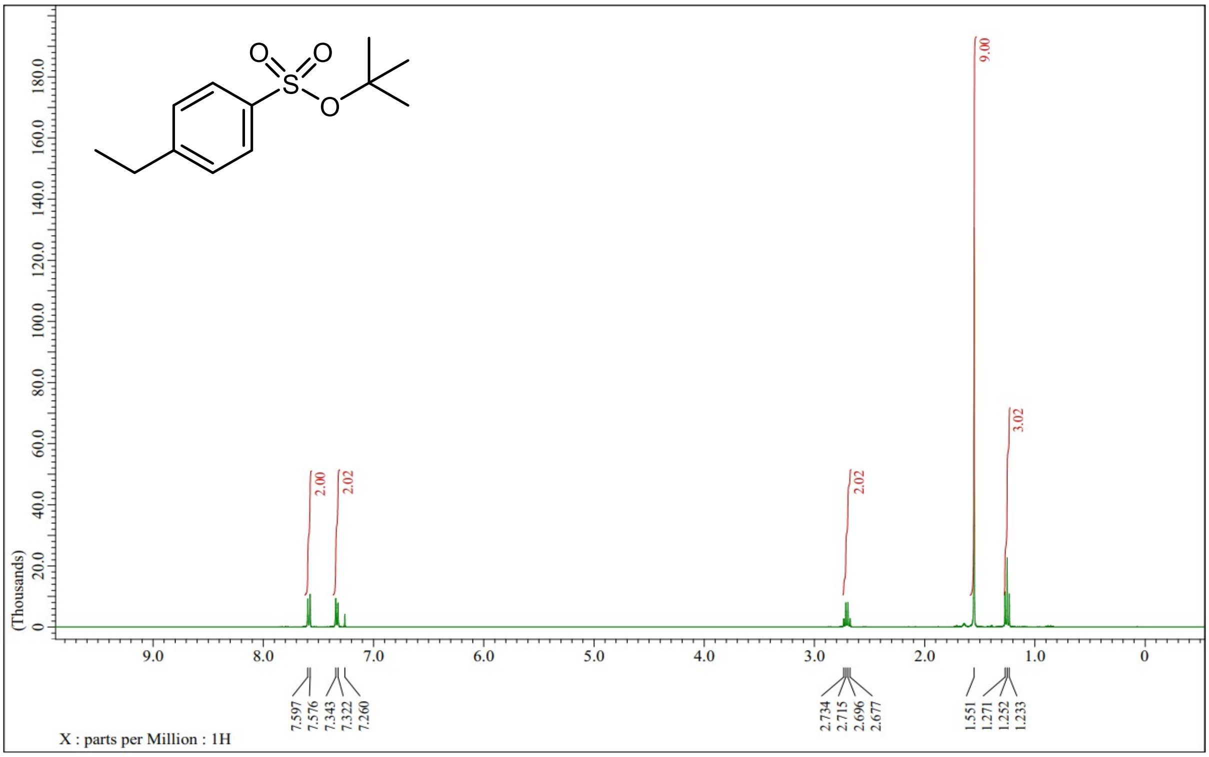

^{1}H NMR spectrum of tert-butyl 4-ethylbenzenesulfonate (4j)

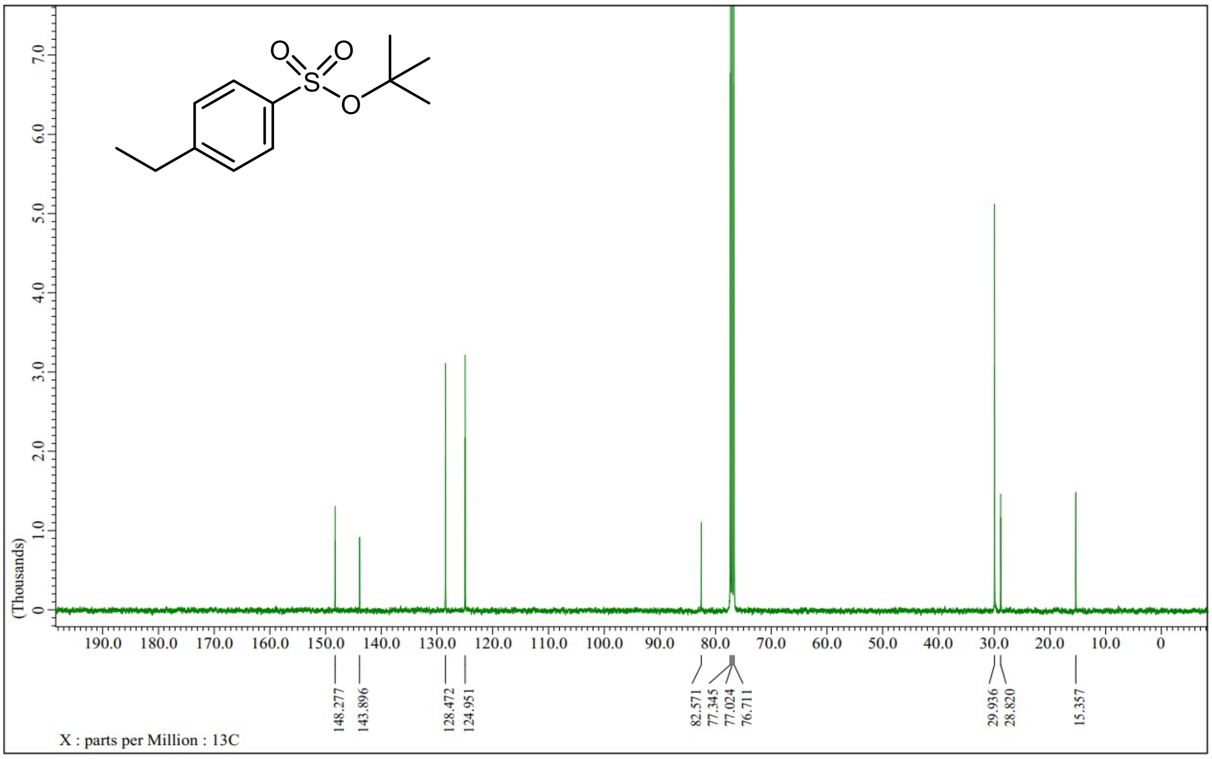

^{13}C NMR spectrum of tert-butyl 4-ethylbenzenesulfonate (4j)

Tert-butyl 4-methoxybenzenesulfonate (4k)

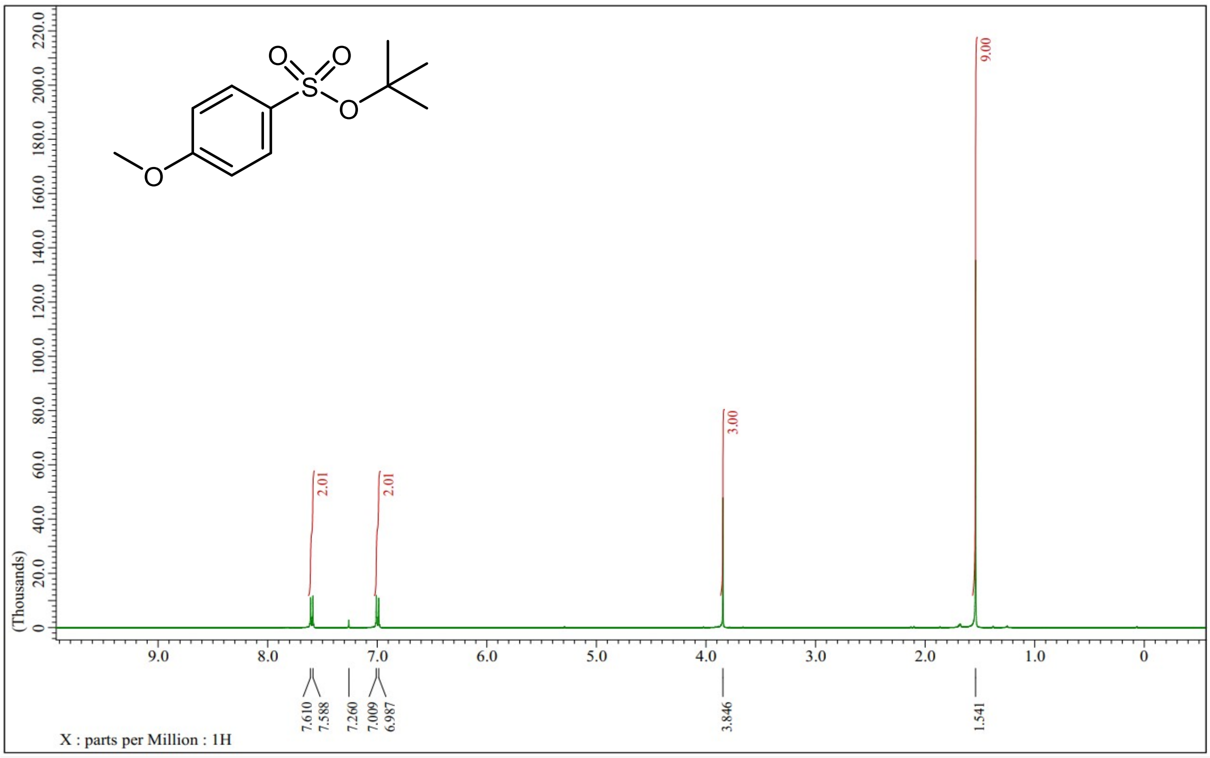

^{1}H NMR spectrum of tert-butyl 4-methoxybenzenesulfonate (4k)

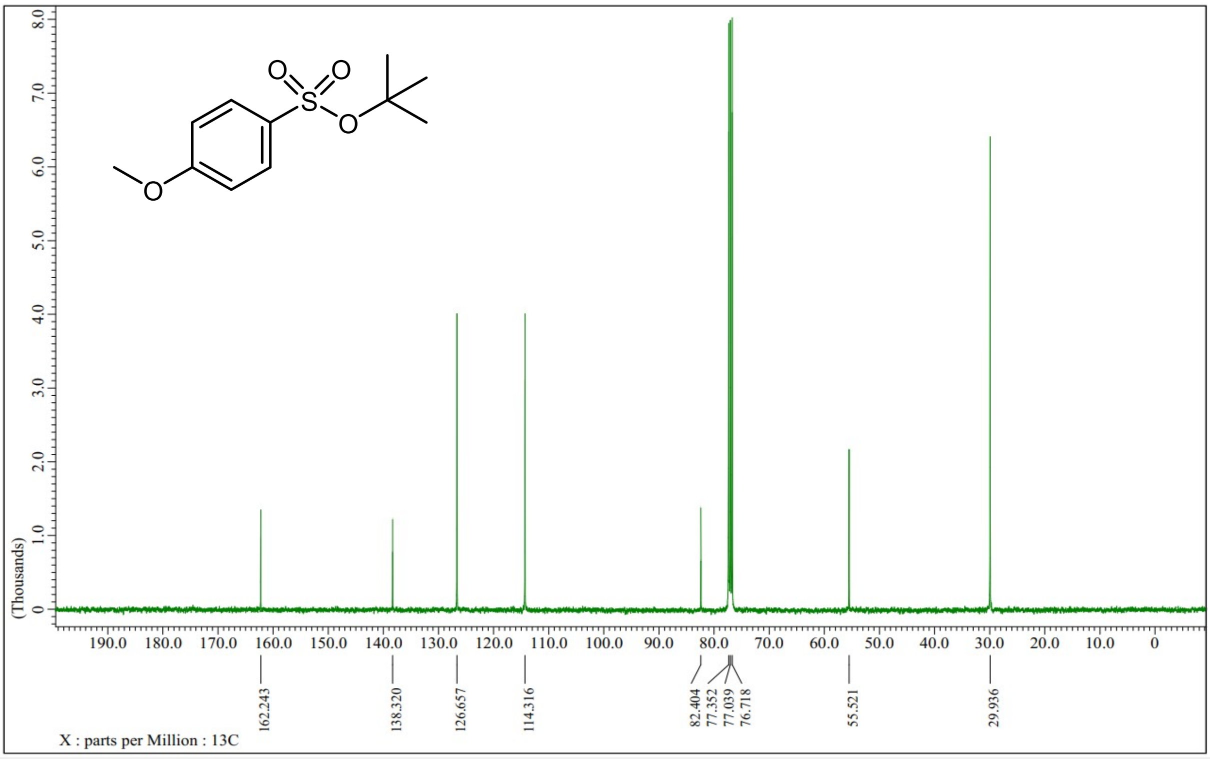

^{13}C NMR spectrum of tert-butyl 4-methoxybenzenesulfonate (4k)

Tert-butyl 4-phenethylbenzenesulfonate (4l)

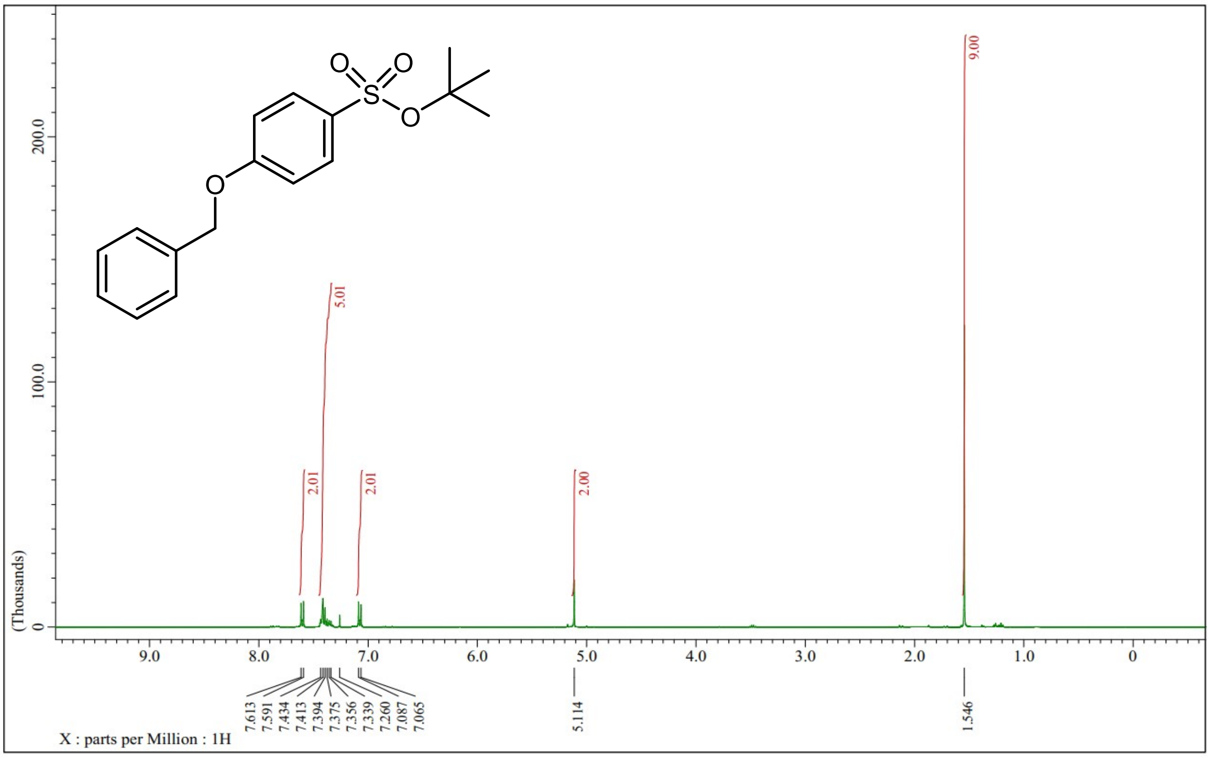

^{1}H NMR spectrum of tert-butyl 4-phenethylbenzenesulfonate (4l)

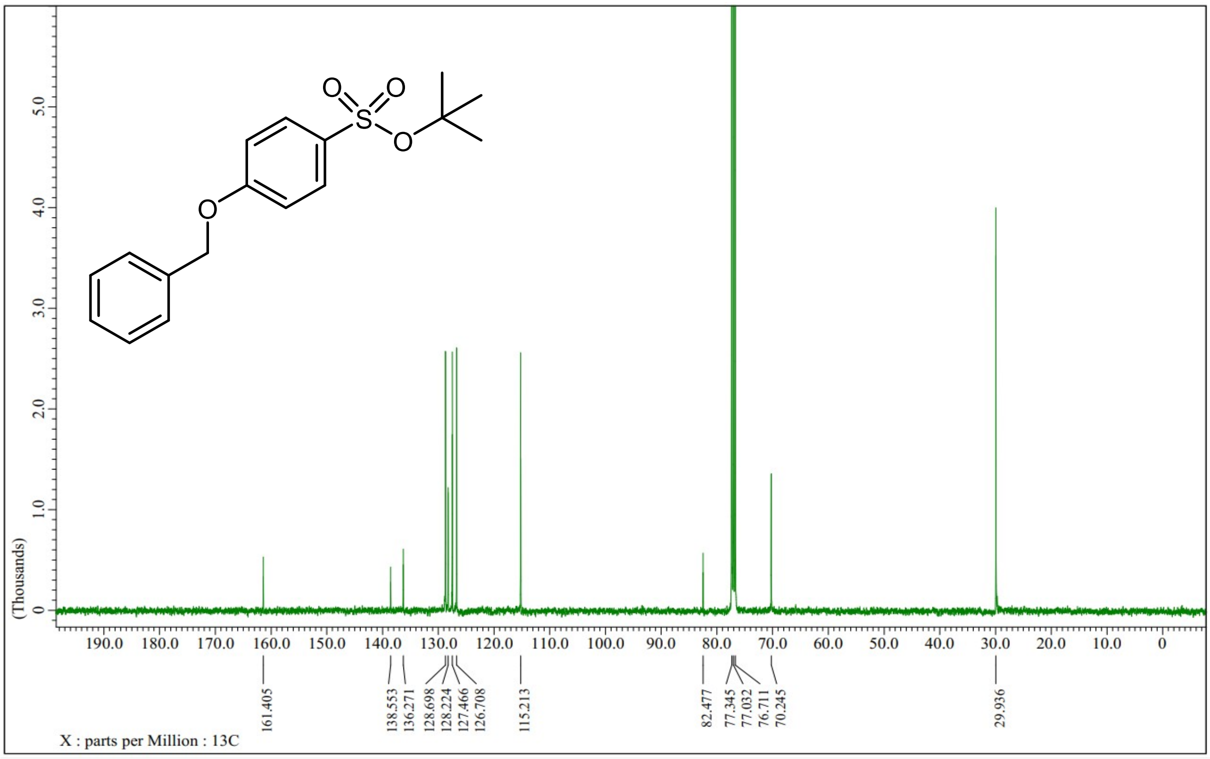

^{13}C NMR spectrum of tert-butyl 4-phenethylbenzenesulfonate (4l)
